# Synthesis of Chrysogeside B from Halotolerant Fungus Penicillium and Its Antimicrobial Activities Evaluation

**DOI:** 10.1038/srep45927

**Published:** 2017-04-11

**Authors:** Ruiquan Liu, Lei Wang, Qibo Li, Min Liao, Zhikun Yang, Yun Huang, Cong Lv, Bing Zheng, Jiangchun Zhong, Qinghua Bian, Min Wang, Shangzhong Liu

**Affiliations:** 1 Department of Applied Chemistry, China Agricultural University, No. 2 West Yuanmingyuan Road, Beijing 100193, P.R. China; 2 Nutrichem Company Limited, No. 27 Life Sciences Park Road, Changping District, Beijing 102206, P.R. China; 3 China Crop Protection Industry Association, Anhuili Siqu Building 16#, Chaoyang District, Beijing 100723, P.R. China

## Abstract

Chrysogeside B, a natural cerebroside, was efficiently synthesized from commercial feedstocks. The bioassays showed that compounds **4**, **5** and **6** exhibited enhanced biological activities compared Chrysogeside B. Further studies revealed that free hydroxyl groups and glycosidic bond have significant impact on the antimicrobial activities. The synthesis of Chrysogeside B and analogues designed to allow identification of the features of this glycolipid required for recognition by tested bacteria and Hela cells is described.

Marine organisms have received widespread attention in the pharmaceutical industry due to the discovery of natural compounds with noteworthy biological activities^1^,^2^, cell growth regulation^3^,^4^, and potential utility for treatment of Alzheimer’s disease^5^,^6^,^7^, etc. In 2011, Peng’s group reported that marine-derived halotolerant fungal strain *Penicillium chrysogenum* could produce Chrysogeside B at 10% salinity that showed antimicrobial activity against *Enterobacter aerogenes* with an MIC value of 1.72 μM^8^ and cytotoxicity against Hela cells.

The importance of Chrysogeside B inspired us to explore the structure activity relationship. Specifically, we are interested to understand how the stereochemistry of glycosidic bond impacts the biological activities. We therefore conducted the enantioselective total synthesis of Chrysogeside B and some variants (Fig. 1). The biological activities were then assayed via growth inhibition studies against *Enterobacter aerogenes*, Hela cells and *Escherichia coli*.

There are generally two strategies for synthesis of cerebrosides. The first one contains the process in which an azide group is incorporated before generation of 1-glycosylated-2-azidosphingosine from the substituted glycosidic ligand, and then azide group is reduced to generates the amine for condensation with α-hydroxyl-β,γ-unsaturated acid^9^,^10^,^11^,^12^. The second approach, used by Wu^13^, Huang^14^, Lim^15^, and Thakur^16^, doesn’t rely on azide group to introduce amino group in sphingosine fragment synthesis.

Our route toward Chrysogeside B entails preparation of the three fragments: glycosidic ligand, sphingosine and α-hydroxyl-β,γ-unsaturated acid. Finally, ceramide is synthesized by combining activated α-hydroxyl-β,γ-unsaturated acid and protected sphingosine followed by glycosylation to produce Chrysogeside B^17^.

## Results and Discussion

### Total synthesis

**Figure 2:**
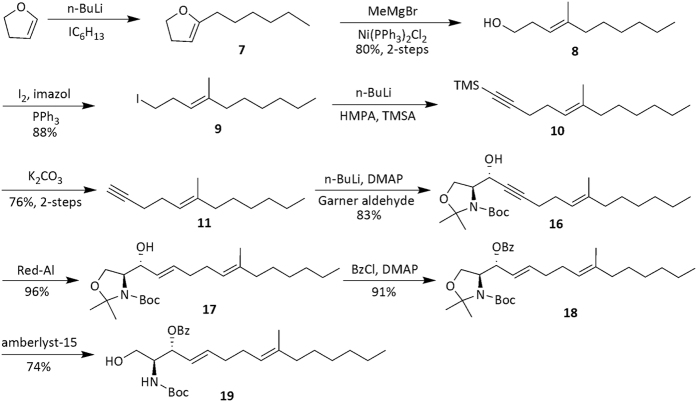
The Basic Skeleton Synthesis of Sphingosine Fragment.

Many syntheses of sphingosine and its analogues^18^,^19^,^20^,^21^ are based on serine or Garner aldehyde^22^,^23^,^24^,^25^,^26^. This chiral building block not only provides the C-2 stereocenter, but enables the introduction of the C-3 stereocenter upon addition of terminal alkynes to form fragment sphingosine^22^,^23^,^24^,^25^,^26^,^27^. We also employed the Garner aldehyde in our synthesis, as shown in Fig. 2. Initially, 2-hexyldihydrofuran **7** at very small amount was produced from dihydrofuran and *t*-BuLi at −78 °C followed by quenching with 1-iodohexane^28^. To scale up preparation of compound **7** at mild condition, we switched to *n*-BuLi at −78 °C, but found that reaction was too sluggish. After the addition of *n*-BuLi at −78 °C, however, the reaction mixture was warmed to 0 °C for 2 h and resulted in metallation of 2,3-dihydrofuran. Treatment of this solution with 1-iodohexane at −30 °C followed by warming to room temperature resulted in complete reaction after 12 h, as judged by TLC. After workup, the reaction product was subjected to a Kumada coupling^9^, providing (3*E*)-alcohol **8** in 80% yield over two steps, characteristic ^1^H NMR data of (3*E*)-alcohol **8**, ^1^H NMR (CDCl_3_, 300 MHz) δ 5.10 (t, *J* = 7.9 Hz, 1H), 3.61 (t, *J* = 6.4 Hz, 2H), 2.27 (q, *J* = 6.8 Hz, 2H). The Appel reaction^9^ was employed to convert the hydroxyl of **8** to iodide (3*E*)-**9** in 88% yield.

In the reaction of compound **9** with ethynyltrimethylsilane, the yield was very low at the beginning because plenty of byproduct terminal alkene formed from elimination of hydrogen iodide. We assumed that *n*-BuLi was not consumed completely during forming lithium salt of trimethylsilylethyne as described by Kenji Mori and Yuji Funaki^29^. Therefore, we tested fully forming lithium of ethynyltrimethylsilane by *n*-BuLi at −10 °C firstly, then adding compound **9** at −78 °C. Such a process could limit the byproduct below 10%. Subsequently, similar process was also employed for nucleophilic addition of Garner aldehyde with terminal alkyne to obtain the desired compound (4*S*,1′*R*,6′*E*)-**16** as a single optical isomer by ^1^H NMR and optical rotation analysis with 83% yield, [α]_D_ = −76.9 (*c* 0.56, CHCl_3_).

We next conducted reducing triple bond to double bond based on the study of Chaudhary Vinodand co-workers^30^, in which metal lithium in ethylamine was used as reductant, and cleanup was very complicated due to lithium is very hard to be accurately measured. Then, Red-Al^31^ was employed with 2.5 equiv to replace metal lithium. After reaction completed, 1 mL of saturated aqueous ammonium chloride was added, desired compound **17** was collected with 96% yield, [α]_D_ = −23.1 (*c* 0.65, CHCl_3_), and characterized by ^1^H NMR, HRMS and ^13^C NMR. The hydroxyl group of compound **17** were protected with benzoyl chloride^17^ with 91% yield, and the isopropylidene was removed by amberlyst-15^32^ to get (2*S*,3*R*,4*E*,8*E*)-sphingosine **19** at yield of 74%, [α]_D_ = −31.7 (*c* 0.87, CHCl_3_). (Refer supplementary information-pages 5–13).

**Figure 3:**
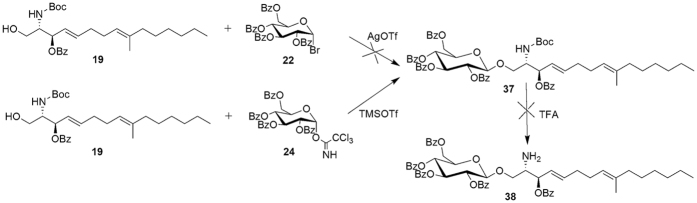
The Attempt of Build Glycosidic Bond.

**Figure 4:**
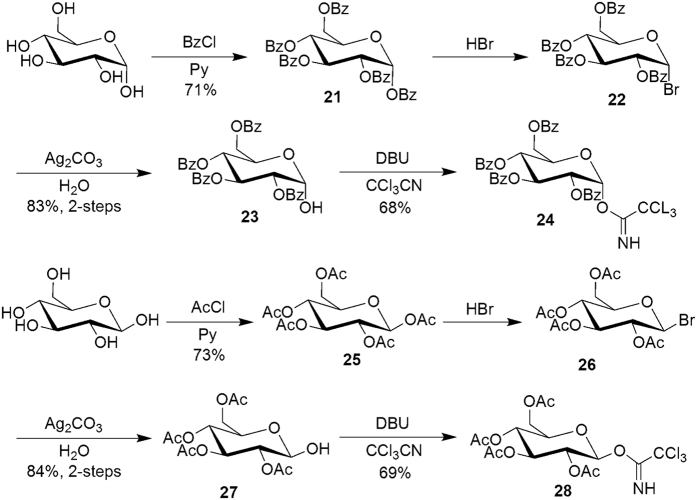
The Synthesis of Glucose Imidoester Compound.

Initially, the method of Murakami and co-workers^17^ was tried to synthesize compound **37** through forming glycosidic bond using tetrabenzoate α-D-Glucopyranosyl bromide **22** and **19** with catalyst AgOTf (Fig. 3). Unfortunately, the yield of product **37** was low, probably due to the fact that compound **19** was unreactive. Next we followed method of Pilgrim and Murphy^33^ to protect α-D-Glucose with benzoyl chloride to generate **21** ([α]_D_ = +142.9 (*c* 0.55, CHCl_3_)). Bromination at C1 with hydrogen bromide furnished 2,3,4,6-tetra-*O*-benzoyl-α-D-glucopyranosyl bromide **22**. Bromide **22** was hydrolyzed in the presence of silver carbonate to obtain 2,3,4,6-tetra-*O*-benzoyl-α-D-glucopyranose **23**, [α]_D_ = +111.4 (*c* 0.55, CHCl_3_). Compound **23** was treated with trichloroacetonitrile in the presence of DBU to generate 2,3,4,6-tetra-*O*-benzoyl-1-(2,2,2-trichloroethanimidate)-α-D-glucopyranoside **24** in 68% yield, [α]_D_ = +95.7 (*c* 0.59, CHCl_3_). 2,3,4,6-Tetra-*O*-acetyl- 1-(2,2,2-trichloroethanimidate)-β-D-glucopyranoside **28** was obtained with the same process for compound **24** in 67% yield, [α]_D_ = +7.9 (*c* 0.83, CHCl_3_) (Fig. 4). (Refer supplementary information-pages 13–17).

According to the method reported by Wu, Douglass and co-workers^14^, imidate **24** was combined with compound alcohol **19** in the presence of TMSOTf. Unfortunately, the glycosidic bond also was cleaved in the Boc deprotection with trifluoroacetic acid. Thus, synthetic pathway was modified to first synthesize ceramide followed by coupling of the ceramide with glycosidic ligand to form glycosidic bond.

**Figure 5:**
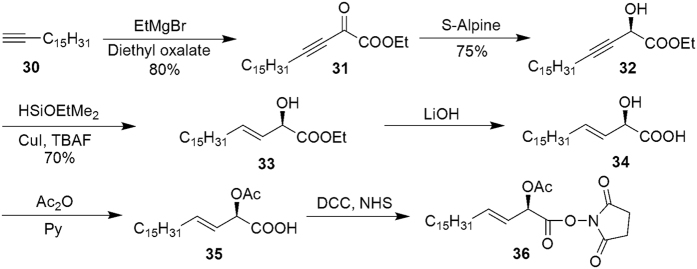
The Synthesis of α-Hydroxyl-β,γ-Unsaturated Acid.

To synthesize the α-hydroxyl-β,γ-unsaturated acid, terminal alkyne **30** was deprotonated with EtMgBr and added to diethyl oxalate. Selective reduction of α-keto-β,γ-acetylenic ester **31** by chiral borane^34^ provided enantiomerically enriched (2*R*)-**32** characterized by ^1^H NMR, [α]_D_ = −26.9 (*c* 0.54, CHCl_3_), 97% *ee*. Compound **32** was treated with HSi(OEt)Me_2_ and catalytic [Cp.Ru(MeCN)_3_]PF_6_ to generate *trans* addition product followed by removing dimethylethoxylsilyl group at low temperature in the presence of copper(I) iodide^34^,^35^ to obtain (2*R*,3*E*)-α-hydroxyl-β,γ-unsaturated ester **33** at yield of 70%, [α]_D_ = −46.7 (*c* 0.55, CHCl_3_). Hydrolysis of the ester and acetylation of the alcohol were conducted. Activation of the acid with N-hydroxylsuccinimide^17^ furnished corresponding activated fatty acid ester (2*R*,3*E*)-**36** (Fig. 5). (Refer supplementary information-pages 17–21).

**Figure 6:**
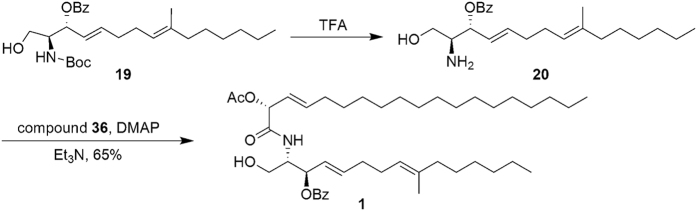
The Synthesis of Ceramide.

According to reported methods^13^,^17^, (2*S*,3*R*,4*E*,8*E*)-sphingosine **20** from compound **19** reacted smoothly with compound **36** in the presence of DMAP to get ceramide **1**, [α]_D_ = +6.5 (*c* 0.70, CHCl_3_), with 65% yield (Fig. 6).

**Figure 7:**
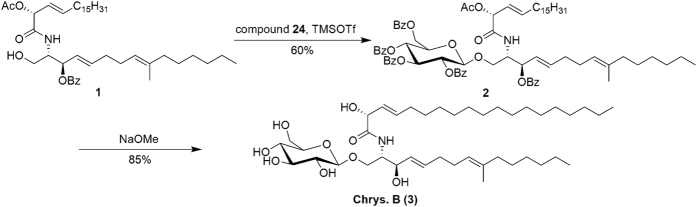
The Synthesis of Chrysogeside B.

It has been noted in the literature that glycoside bond formation to synthesize cerebrosides from ceramide can lead to inversion of the glycosidic bond and epimerization at C2^30^. These undesired isomerizations can be limited through optimization of reaction conditions^17^,^36^,^37^,^38^. Thus, we conducted a series of optimization experiments including solvents, temperature and catalyst loading, and found that when reactions were conducted under anhydrous conditions with diethyl ether/tetrahydrofuran (2:1, v/v) using 0.05 equiv TMSOTf as catalyst at −30 °C, no isomerization was found by NMR and the desired protected β-glucoside **2** was obtained with 60% yield, [α]_D_ = +15.2 (*c* 1.14, CHCl_3_). Finally, sodium methoxide was used in the deprotection, resulting in the target product Chrysogeside B (**3**) in 85% yield, characterized by NMR spectra and [α]_D_ = −8.1 (*c* 0.5, CH_3_OH) agreed well with lit.^8^ [α]_D_ = −8.0 (*c* 0.5, CH_3_OH) (Fig. 7). Compound **4** was synthesized with the same process for compound **2** from acetylated glycosyl donor **28** in 50% yield, and compounds **5** and **6** were prepared as the same as the process for Chrysogeside B with 80% and 89% yield. (Refer supplementary information-pages 21–25).

### Antimicrobial activities and cytotoxic assays

**Figure 8:**
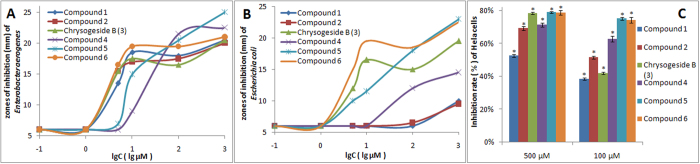
Antimicrobial activities and cytotoxic assays. (**A**,**B**) The antimicrobial activities with synthetic compounds **1**–**6** against *Enterobacter aerogenes* and *Escherichia coli*. Incubation after 24 h, and zones of inhibition (mm in diameter) were recorded. (**C**) The cytotoxic assays against Hela cells with synthetic compounds **1**–**6** at different concentrations by the MTT method. Data are expressed as means ± SD of the inhibition rate of Hela cells by synthetic compounds **1**–**6** at 100, 500 μM. *P < 0.01 vs control.

According to the report of Peng’s group^8^, the antimicrobial activities against *Enterobacter aerogenes* were evaluated by an agar dilution method (Fig. 8) (refer supplementary information-pages 67–71). As the results showed, the antimicrobial activities of compounds **1**, **2**, **4**–**6** were better than Chrysogeside B at 100 and 1,000 μM. The antimicrobial activities of analogues **1**, **2**, **6** were also better than Chrysogeside B at 5 and 10 μM, their MIC were less than 5 μM, the corresponding ceramide elicited better antimicrobial activity than Chrysogeside B. Replacing β-glycosidic bond in Chrysogeside B with α-glycosidic bond, the diastereoisomer of Chrysogeside B at higher concentrations displayed higher antimicrobial activities.

This result suggests that the glycosidic bond of chrysogesides exerts a greater influence on antimicrobial activities, and the fully protected compounds **2** and **4** also have weak antibacterial activity. The same result is shown in the antimicrobial activities of *Escherichia coli* and cytotoxic assays, especially at 100 μM (Fig. 8). Compounds **5**, **6** showed antimicrobial activities against *Escherichia coli* with MIC less than 5 μM, and cytotoxic effects against Hela cells with IC_50_ less than 100 μM.

## Conclusion

In conclusion, we presented a convergent synthetic approach to Chrysogeside B and five of its analogues based on the use of two chiral building blocks prepared by means of catalytic diastereoselective reactions. Based on results from assays, it was found that the free hydroxyl groups and glycosidic bond have significant impact on antimicrobial activities and cytotoxicities of cerebrosides and ceramide against *Enterobacter aerogenes* and Hela cells. These results are very helpful for optimizing glycolipid structures for *Enterobacter aerogenes* inhibitors.

## Methods

### General Information

^1^H NMR and ^13^C NMR spectra were recorded on a Bruker Avance DPX 300 MHz instrument (Bruker, Billerica, MA 01821-3991, USA), TMS as the internal standard. ^1^H NMR data are reported as follows: chemical shift, multiplicity (s = singlet; d = doublet; q = quartet; m = multiplet; br = broad), coupling constant (Hz), and integral. Data for ^13^C NMR spectra are reported in terms of chemical shift. Mass spectrometric data were obtained on Agilent Accurate-Mass-Q-TOF MS 6520 system equipped with an Electrospray ionization (ESI) source (Agilent, Santa Clara, CA 95051, USA). Specific rotations were obtained on a High Accurary Polarimeter Rudolph Autopl VI (Rudolph, Wilmington, Massachusetts 01887, USA). Toluene and DCM were freshly distilled after dried by calcium hydride under nitrogen, diethyl ether and THF were freshly distilled after dried by Lithium aluminum hydride. Unless otherwise stated, all reagents were commercially available and were used without purification. Organic solutions were concentrated under reduced pressure on a rotary evaporator or an oil pump. Reactions were monitored through thin layer chromatography (TLC) on silica gel-precoated glass plates (0.25 mm thickness, SiliCycle silica gel). Flash column chromatography was performed using Qingdao Haiyang flash silica gel (200–300 mesh).

### (2*R*,3*E*)-2-acetoxy-N-[(2*S*,3*R*,4*E*,8*E*)-1-hydroxy-3-benzoyloxy-9-methylpentadec-4,8-dien-2-yl]nonadec-3-enamide 1

Triethylamine (0.15 mL, 1.50 mmol) was added to a solution of compound **20** (447 mg, 1.20 mmol), compound **36** (541 mg, 1.20 mmol), DCM (30 mL) and 4-dimethylaminopyridine (10 mg) at room temperature, and the reaction mixture was stirred for overnight. After the reaction was completed by TLC detection, the solution was concentrated under vacuum. The residue was purified using silica gel chromatography (25% ethyl acetate in hexanes) to give compound **1** as a colorless amorphous solid 552 mg, yield: 65%. Analytical data for **1**: [α]_D_ = +6.5 (*c*0.70, CHCl_3_); ^1^H NMR (300 MHz,CDCl_3_) δ 8.05 (d, *J* = 7.9 Hz, 2H, Ar-H), 7.61 (t, *J* = 6.8 Hz, 1H, Ar-H), 7.48 (t, *J* = 7.6 Hz, 2H, Ar-H), 6.79 (d, *J* = 8.4 Hz, 1H, C=ONH), 6.03–5.00 (m, 7H, CH=CH, OAcCH, OBzCH), 4.33–4.13 (m, 1H, NHCH), 3.83–3.62 (m, 2H, CH_2_O), 2.26–1.56 (m, 11H, OAc, CH=CHCH_2_), 1.49 (s, 3H, CH=CHCH_3_), 1.42–1.14 (m, 34H, CH_2_), 0.87 (t, *J* = 6.6 Hz, 6H, CH_2_CH_3_); ^13^C NMR (75 MHz, CDCl_3_) δ169.4, 169.1, 166.7, 138.2, 137.3, 137.1, 133.7, 131.0, 123.0, 129.8, 128.7, 126.2, 125.5, 124.9, 123.1, 74.8, 74.7, 61.7, 54.0, 39.8, 32.7, 32.5, 32.1, 31.8, 30.3, 29.9, 29.8, 29.6, 29.5, 29.4, 29.3, 29.1, 28.9, 28.1, 28.0, 27.4, 27.3, 26.9, 22.8, 21.1, 16.2, 16.0, 14.2; HRMS (ESI): *m/z* [M+Na]^+^ calcd for C_44_H_71_NNaO_6_: 732.5174; found: 732.5178.

### (2*R*,3*E*)-2-acetoxy-N-[(2*S*,3*R*,4*E*,8*E*)-1-(2,3,4,6-tetrabenzoyloxy-l-β-D-glucopyranosyloxy)-3-benzoyloxy-9-methylpentadec-4,8-dien-2-yl]nonadec-3-enamide 2

Mixed solvents (anhydrous ethyl ether/THF = 2:1, 5 mL) was added to a solution of compound **1** (100 mg, 0.14 mmol), compound **24** (121 mg, 0.15 mmol), 4A molecular sieve (1 g) under the protection of nitrogen. The mixed solution was stirred at room temperature for 1 h, and was cooled to −30 °C, and then trimethylsilyl trifluoromethanesulfonate (0.65 uL) was dropped. After the reaction was completed by TLC detection, triethylamine (1 mL) was added to the solution. The mixture was filtered and the filtrate was concentrated under vacuum. The residue was purified using silica gel chromatography (20% ethyl acetate in hexanes) to give compound **2** as a colorless amorphous solid 108 mg, yield: 60%. Analytical data for **2**: [α]_D_ = +15.2 (*c*1.14, CHCl_3_); ^1^H NMR (300 MHz,CDCl_3_) δ 8.10 (d, *J* = 7.2 Hz, 2H, Ar-H), 8.00–7.81 (m, 6H, Ar-H), 7.72–7.30 (m, 17H, Ar-H), 6.44 (d, *J* = 9.2 Hz, 1H, C=ONH), 6.02–5.84 (m, 2H, BzOCHCH=CH, AcOCHCH=CH), 5.84–5.44 (m, 6H, CH_2_CH=CH, H-3, H-4, H-2), 6.02–5.21 (m, 2H, OAcCH, OBzCH), 4.79 (d, *J* = 8.0 Hz, 1H, H-1), 4.56–4.25 (m, 3H, NHCH, H-6), 4.12–4.00 (m, 1H, H-5), 3.69–3.56 (m, 1H, CH_a_H_b_O), 3.41–3.26 (m, 1H, CH_a_H_b_O), 2.10 (s, 3H, OAc), 2.04–1.65 (m, 8H, CH=CHCH_2_), 1.45 (s, 3H, CH=CHCH_3_), 1.39–1.15 (m, 34H, CH_2_), 0.88 (t, *J* = 6.1 Hz, 6H, CH_2_CH_3_); ^13^C NMR (75 MHz, CDCl_3_) δ69.4, 168.6, 166.1, 165.9, 165.3, 138.0, 136.1, 133.7, 133.6, 133.5, 133.4, 133.2, 133.1, 130.1, 123.0, 129.9, 129.8, 129.6, 129.5, 129.3, 129.2, 129.1, 128.9, 128.8, 128.7, 128.6, 128.5, 125.6, 122.9, 101.0, 72.4, 72.3, 69.7, 63.1, 50.9, 38.0, 32.4, 32.1, 31.7, 29.8, 29.6, 29.5, 29.4, 28.8, 27.1, 23.5, 22.8, 22.7, 20.8, 14.2, 14.1; HRMS (ESI): *m/z* [M+Na]^+^ calcd for C_78_H_97_NNaO_15_: 1310.6749; found: 1310.6742.

### Chrysogeside B (3)

Sodium methoxide solution (0.05 mL 0.5 *M* in methanol, 0.025 mmol) was added to the solution of compound **2** (90 mg, 0.09 mmol) and anhydrous methanol (5 mL) at 0 °C, and the solution was stirred at room temperature for 2 h. After the reaction was completed by TLC detection, ambrest 15 was added to adjust pH 6–7. After the mixture was filtered and filtrate was concentrated under vacuum. The residue was purified using silica gel chromatography (10% methanol /acetate in chloroform) to give compound **3** as a colorless amorphous solid 43 mg, yield: 85%. Analytical data for **3**: [α]_D_ = −8.1 (c 0.5, CH_3_OH); (lit.^9^ [α]_D_ = −8.0 (*c* 0.5, CH_3_OH)); ^1^H NMR (300 MHz, CD_3_OD) δ 5.96–5.80 (m, 1H, HOCHCH=CH), 5.80–5.67 (m, 1H, HOCHCH=CH), 5.66–5.26 (m, 3H, CH_2_CH=CH), 4.63–4.39 (m, 1H, O=CCHOH), 4.27 (d, *J* = 7.6 Hz, 1H, H-1), 4.23–4.05 (m, 3H, COCH_2_, NHCHCHOH), 4.04–3.92 (m, 1H, NHCHCHOH), 3.92–3.81 (m, 1H, H-3), 3.80–3.60 (m, 2H, H-4, H-2), 3.30–3.26 (m, 2H, H-6), 3.25–3.15 (m, 1H, H-5), 2.36–1.89 (m, 6H, CH=CHCH_2_), 1.73–1.48 (m, 2H, CH_2_(CH_2_)_4_CH_3_), 1.43 (s, 3H, CH=CHCH_3_), 1.29 (s, 34H, CH_2_), 0.90 (t, *J* = 6.6 Hz, 6H, CH_2_CH_3_); ^13^C NMR (75 MHz, CD_3_OD) δ 175.5, 135.6, 135.1, 134.8, 132.4, 130.9, 129.9, 129.0, 104.7, 78.0, 75.0, 74.1, 73.3, 72.9, 71.6, 69.6, 62.7, 55.0, 42.8, 34.0, 33.4, 33.1, 31.2, 30.8, 30.7, 30.4, 30.2, 26.9, 25.0, 24.6, 23.7, 19.5, 14.4; HRMS (ESI): *m/z* [M+Na]^+^ calcd for C_41_H_75_NNaO_9_: 748.5334; found: 748.5338.

### (2*R*,3*E*)-2-Acetoxy-N-[(2*S*,3*R*,4*E*,8*E*)-1-(2,3,4,6-tetraacetyloxy-l-α-D-glucopyranosyloxy)-3-benzoyloxy-9-methylpentadec-4,8-dien-2-yl]nonadec-3-enamide 4

With the same process for the synthesis of **2**, the compound **4** was obtained from compound **28** and compound **1** as a colorless amorphous solid 92 mg, yield: 50%. Analytical data for **4**: [α]_D_ = +19.3 (*c* 0.76, CHCl_3_); ^1^H NMR (300 MHz, CDCl_3_) δ 8.02 (d, *J* = 7.4 Hz, 2H, Ar-H), 7.57 (t, *J* = 7.2 Hz, 1H, Ar-H), 7.44 (t, *J* = 7.4 Hz, 2H, Ar-H), 6.47 (d, *J* = 9.2 Hz, 1H, C=ONH), 6.03–5.73 (m, 2H, BzOCHCH=CH, AcOCHCH=CH), 5.73–5.41 (m, 5H, CH_2_CH=CH, H-3, H-4), 5.41–5.02 (m, 2H, OAcCH, OBzCH), 4.99 (d, *J* = 3.6 Hz, 1H, H-1), 4.50–4.36 (m, 1H, H-2), 3.86–3.72 (m, 2H, H-5, H-6a), 4.19–4.01 (m, 2H,H-6b, NHCH), 3.86–3.72 (m, 1H CH_a_H_b_O), 3.66–3.45 (m, 1H, CH_a_H_b_O), 2.18–2.01 (m, 15H, OAc), 1.99–1.60 (m, 8H, CH=CHCH_2_), 1.47 (s, 3H, CH=CHCH_3_), 1.44–1.11 (m, 34H, CH_2_), 0.87 (t, *J* = 5.6 Hz, 6H CH_2_CH_3_); ^13^C NMR (75 MHz, CDCl_3_) δ 169.6, 169.5, 169.2, 168.5, 168.4, 168.3, 168.0, 137.1, 135.0, 132.6, 132.2, 128.8, 128.7, 127.6, 127.4, 124.5, 121.9, 95.9, 73.7, 73.6, 73.4, 71.5, 70.9, 67.3, 60.8, 60.4, 52.8, 36.9, 32.9, 31.3, 30.9, 30.6, 28.7, 28.6, 28.5, 28.3, 27.7, 27.6, 24.6, 23.9, 22.3, 21.7, 21.5, 19.8, 19.5, 18.2, 13.1, 13.0; HRMS (ESI): *m/z* [M+Na]^+^ calcd for C_58_H_89_NNaO_15_: 1062.6114; found: 1062.6107.

### (2*R*,3*E*)-2-Hydroxy-N-[(2*S*,3*R*,4*E*,8*E*)-l-α-D-glucopyranosyloxy-3-hydroxy-9-methylpentadec-4,8-dien-2-yl]nonadec-3-enamide 5

With the same process for the synthesis of **3**, the compound **5** was obtained from compound **4** and sodium methoxide solution (0.5 *M* in methanol) as a colorless amorphous solid 41 mg, yield: 80%. Analytical data for **5**: [α]_D_ = +5.4 (*c* 0.50, CH_3_OH); ^1^H NMR (300 MHz, CD_3_OD) δ 5.96–5.81 (m, 1H, HOCHCH=CH), 5.80–5.67 (m, 1H, HOCHCH=CH), 5.66–5.22 (m, 3H, CH_2_CH=CH), 4.72–4.44 (m, 2H, O=CCHOH, H-1), 4.32–4.06 (m, 3H, COCH_2_, NHCHCHOH), 4.06–3.93 (m, 1H, NHCHCHOH), 3.94–3.80 (m, 1H, H-3), 3.80–3.48 (m, 3H, H-4, H-2, H-5), 3.30–3.14 (m, 2H, H-6), 2.36–1.47 (m, 8H, CH=CCH_2_, CH=CHCH_2_), 1.45 (s, 3H, CH=CHCH_3_), 1.41–1.23 (m, 34H, CH_2_), 0.92 (t, *J* = 6.7 Hz, 6H, CH_2_CH_3_); ^13^C NMR (75 MHz, CD_3_OD) δ 175.6, 135.4, 134.6, 134.3, 100.3, 129.0, 103.3, 78.1, 76.2, 75.8, 74.1, 73.3, 71.8, 62.0, 56.4, 42.3, 34.0, 33.4, 33.0, 31.2, 30.8, 30.7, 30.6, 30.4, 30.2, 26.9, 25.0, 24.6, 23.7, 20.9, 14.4; HRMS (ESI): *m/z* [M+Na]^+^ calcd for C_41_H_75_NNaO_9_: 748.5334; found: 748.5335.

### (2*R*,3*E*)-2-Hydroxy-N-[(2*S*,3*R*,4*E*,8*E*)-1-hydroxy-3-hydroxy-9-methylpentadec-4,8-dien-2-yl]nonadec-3-enamide 6

With the same process for the synthesis of **3**, the compound **6** was obtained from compound **1** and sodium methoxide solution (0.5 *M* in methanol) as a colorless amorphous solid 71 mg, yield: 89%. Analytical data for **6**: [α]_D_ = −6.2 (*c* 0.65, CH_3_OH); ^1^H NMR (300 MHz,CDCl_3_) δ 6.09–5.67 (m, 2H, HOCHCH=CH), 6.67–5.03 (m, 3H, CH_2_CH=CH), 4.72–4.14 (m, 3H, CHNH, CHOH), 3.99–3.39 (m, 2H, CH_2_O), 2.35–1.49 (m, 8H, CH=CHCH_2_), 1.48–0.93 (m, 37H, CH=CHCH_3_,CH_2_), 0.88 (t, *J* = 5.8 Hz, 6H, CH_3_); ^13^C NMR (75 MHz, CD_3_OD) δ 175.7, 135.7, 134.7, 130.9, 129.0, 128.5, 127.7, 74.0, 73.2, 62.0, 56.6, 42.2, 34.0, 33.4, 33.0, 31.2, 30.8, 30.7, 30.6, 30.4, 30.3, 30.2, 28.9, 26.9, 25.0, 23.7, 14.4; HRMS (ESI): *m/z* [M+H]^+^ calcd for C_35_H_66_NO_4_: 564.4991; found: 564.4997.

### Bioassay Protocols

#### Antimicrobial Assays

The antimicrobial activities against *Enterobacter aerogenes* (ATCC51697) and *Escherichia coli* (ATCC13048) were evaluated by an agar dilution method. The tested strains were cultivated in Nutrient agar plates and Luria-Bertani agar plates for bacteria at 37 °C. Compounds **1**–**6** and positive controls were dissolved in methanol at different concentrations from 1,000 to 0.1 μM by the continuous 10-fold dilution methods and 2-fold dilution methods. A 5 μL quantity of test solution was absorbed by a paper disk (6 mm diameter) and placed on the assay plates. After 24 h incubation, zones of inhibition (mm in diameter) were recorded. Ciprofloxacin (5 μg/disk), and Gentamicin (10 μg/disk) and methanol (5 μL/disk) were used as positive control and blank control for *Enterobacter aerogenes* and *Escherichia coli* with zones of inhibition (mm in diameter) of 26.5, 24.0, 6.0, and 28.5, 21.5, 6.0 mm, respectively.

#### Cytotoxic Assays

Cytotoxicity was assayed by the MTT methods. Hela cells line was grown in DMEM supplemented with 10% FBS under a humidified atmosphere of 5% CO_2_ and 95% air at 37 °C. Cell suspension (100 μL, a density of 5 × 10^4^ cell mL^−1^) was plated in 96-well microtiter plates and incubated for 24 h. Then, 100 μL of the test solutions (in DMEM), which was at different concentrations between 500 and 100 μM by the dilution methods, were added to each well and further incubated for 72 h. The MTT solution (20 μL, 5 mg/mL in IPMI-1640 medium) was then added to each well and incubated for 4 h. Old medium containing MTT (150 μL) was then gently replaced by DMSO, and shaking was conducted to dissolve completely formazan crystals formed. Absorbance was then determined on a Spectra Max Plus plate reader at 570 nm.

## Additional Information

**How to cite this article:** Liu, R. *et al*. Synthesis of Chrysogeside B from Halotolerant Fungus Penicillium and Its Antimicrobial Activities Evaluation. *Sci. Rep.* **7**, 45927; doi: 10.1038/srep45927 (2017).

**Publisher's note:** Springer Nature remains neutral with regard to jurisdictional claims in published maps and institutional affiliations.

## Supplementary Material

Supplementary Information

## Figures and Tables

**Figure 1:**
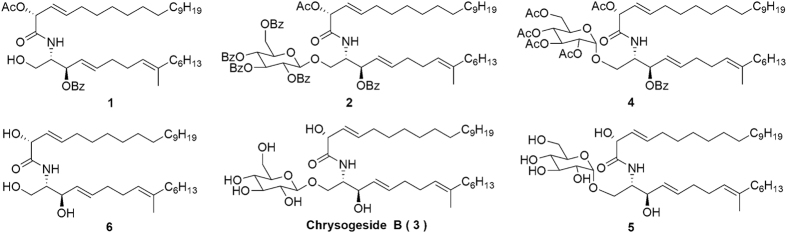
The Chrysogeside B and Some Variants.
