# Supplementary material for: Synthesis of Chrysogeside B from Halotolerant Fungus Penicillium and Its Antimicrobial Activities Evaluation
Source: Sci Rep. 2017 Apr 11;7:45927. doi: 10.1038/srep45927 (PMC5387731; doi:10.1038/srep45927)
Supplement: Supplementary Information [file srep45927-s1.pdf]

Supplementary Information

Synthesis of Chrysogeside B from Halotolerant Fungus Penicillium and Its Antimicrobial Activities Evaluation

Ruiquan Liu[1], Lei Wang[2], Qibo Li[1], Min Liao[1], Zhikun Yang[1], Yun Huang[1], Cong Lv[3], Bing Zheng[1], Jiangchun Zhong[1], Qinghua Bian[1], Min Wang[1] and Shangzhong Liu[1,*]

[1] Department of Applied Chemistry, China Agricultural University, 2 West Yuanmingyuan Road, Beijing 100193, P. R. China.

[2] Nutrichem Company Limited, No. 27 Life Sciences Park Road, Changping District, Beijing 102206, P. R. China.

[3] China Crop Protection Industry Association, Anhuili Siqu Building 16#, Chaoyang District, Beijing 100723, P. R. China.

[*] Corresponding author. E-mail:shangzho@cau.edu.cn

Contents

Bioassay Protocols 2

General Information 2-3

Total Synthetic Process 4

Preparation of Alkyne Contained Methyl Branched 5–7

Preparation of Garner Aldehyde 7–9

Preparation of Sphingosine Fragment 9–13

Preparation of Glucose Hydroxyl Compound 13–17

Preparation of α-Hydroxyl-β,γ-Unsaturated Acid 17–21

Preparation of Chrysogeside B and Analogues 21–25

^{1}H and ^{13}C NMR Spectra of All Products 26–51

HRMS Date and MS Date of All Products 52–65

HPLC Spectra of Compound **32** 66

Bioassay Protocols Data 67–71

References 72

Bioassay Protocols

Antimicrobial Assays. The antimicrobial activities against *Enterobacter aerogenes* (ATCC51697) and *Escherichia coli* (ATCC13048) were evaluated by an agar dilution method. The tested strains were cultivated in Nutrient agar plates and Luria-Bertani agar plates for bacteria at 37 °C. Compounds **1**-**6** and positive controls were dissolved in methanol at different concentrations from 1000 to 0.1 μM by the continuous 10-fold dilution methods and 2-fold dilution methods. A 5 μL quantity of test solution was absorbed by a paper disk (6 mm diameter) and placed on the assay plates. After 24 h incubation, zones of inhibition (mm in diameter) were recorded. Ciprofloxacin (5 μg/disk), Gentamicin (10 μg/disk) and blank control (5 μL methanol/disk) was used as positive control for *Enterobacter aerogenes* and *Escherichia coli* with zones of inhibition (mm in diameter) of 26.5, 24.0, 6.0, and 28.5, 21.5, 6.0 mm, respectively.

Cytotoxic Assays. Cytotoxicity was assayed by the MTT methods. In the MTT assay, Hela cells line was grown in DMEM supplemented with 10% FBS under a humidified atmosphere of 5% CO_2 and 95% air at 37 °C. Cell suspension (100 μL, a density of 5 × 10^4 cell mL^{-1}) was plated in 96-well microtiter plates and incubated for 24 h. Then, 100 μL of the test solutions (in DMEM), which was at different concentrations between 500 and 100 μM by the dilution methods, were added to each well and further incubated for 72 h. The MTT solution (20 μL, 5 mg/mL in IPMI-1640 medium) was then added to each well and incubated for 4 h. Old medium containing MTT (150 μL) was then gently replaced by DMSO, and shaking was conducted to dissolve completely formazan crystals formed Absorbance was then determined on a Spectra Max Plus plate reader at 570 nm.

General Information

^{1}H NMR and ^{13}C NMR spectra were recorded on a Bruker Avance DPX 300 MHz instrument (Bruker, Billerica, MA 01821-3991, USA), TMS as the internal standard. ^{1}H NMR data are reported as follows: chemical shift, multiplicity (s = singlet; d = doublet; q = quartet; m = multiplet; br = broad), coupling constant (Hz), and integral. Data for ^{13}C NMR spectra are reported in terms of chemical shift. Mass spectrometric data were obtained on Agilent Accurate-Mass-Q-TOF MS 6520 system equipped with an Electrospray ionization (ESI) source (Agilent, Santa Clara, CA 95051, USA). Specific rotations were obtained on a High Accurary Polarimeter Rudolph Autopl VI (Rudolph, Wilmington, Massachusetts 01887, USA). Toluene and DCM were freshly distilled after dried by calcium hydride under nitrogen,

diethyl ether and THF were freshly distilled after dried by Lithium aluminum hydride. Unless otherwise stated, all reagents were commercially available and were used without purification. Organic solutions were concentrated under reduced pressure on a rotary evaporator or an oil pump. Reactions were monitored through thin layer chromatography (TLC) on silica gel-precoated glass plates (0.25 mm thickness, SiliCycle silica gel). Flash column chromatography was performed using Qingdao Haiyang flash silica gel (200-300 mesh).

Total Synthetic Process

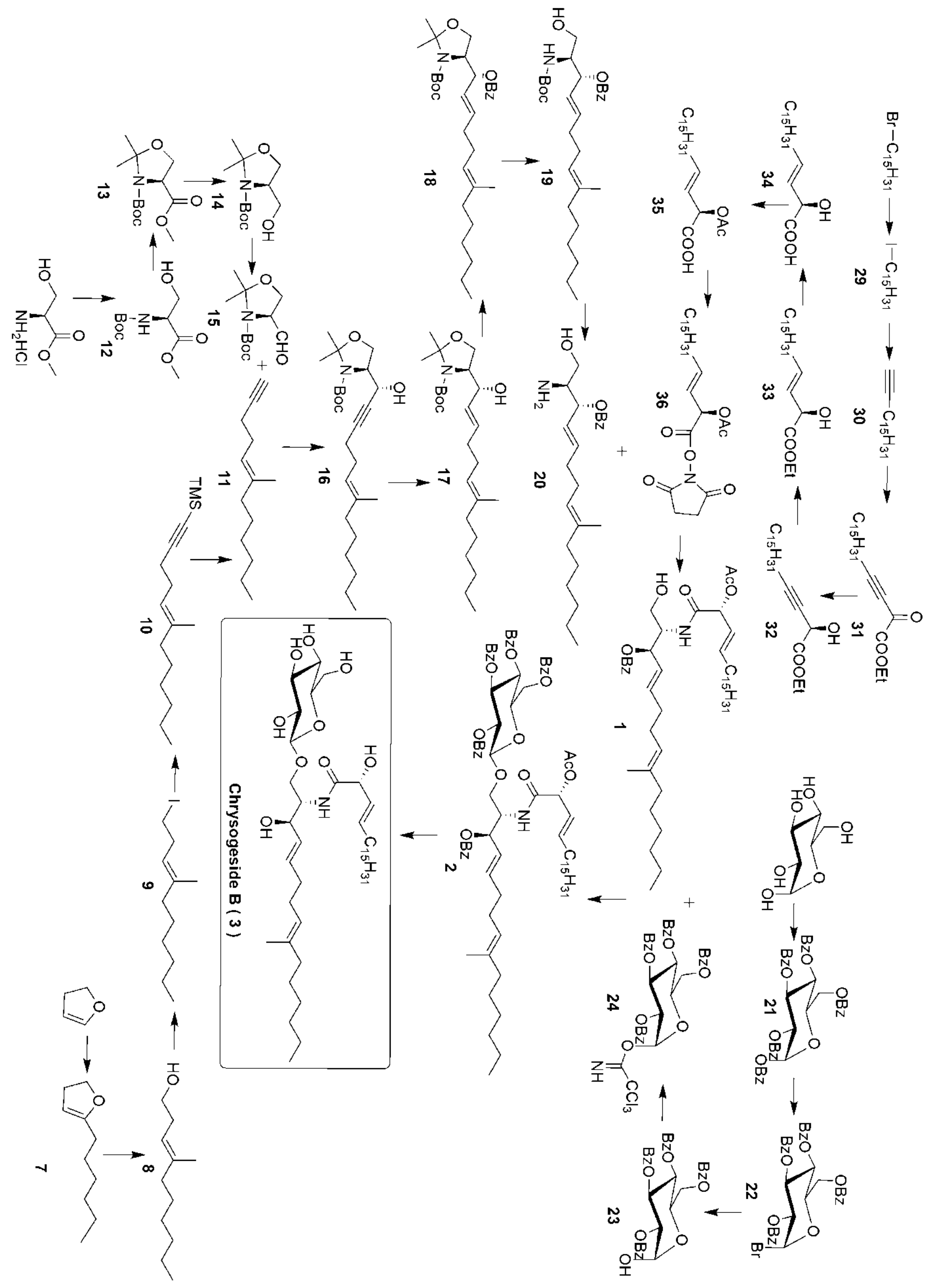

Preparation of Alkyne Contained Methyl Branched

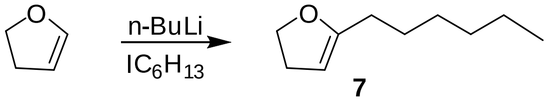

2-Hexyl-2,3-dihydrofuran 7

n-Butyllithium solution (54.00 mL of a 2.4 *M* solution in hexane, 129.60 mmol) was added dropwise to a solution of anhydrous THF and 2.3-dihydrofuran (9.69 g, 120.00 mmol) at -78 °C in the atmosphere of nitrogen. The solution was stirred for 30 min at -78 °C, and then warmed to 0 °C and maintained for 2 h. Then the reaction mixture was allowed to cool to -30 °C, 1-iodohexane (14.46 mL, 96.00 mmol) was added and stirred for 2 h. The resulting mixture was warmed to room temperature. After the reaction was completed by TLC detection, saturated aqueous ammonium chloride (50 mL) was added to quench the reaction, and the product was extracted with ethyl ether (3 × 50 mL). The combined organic phase was washed by saturated sodium chloride solution (3 × 100 mL), dried over sodium sulfate and concentrated under vacuum to get colorless transparent oil (14.80 g) as compound **7,** which was directly used in the next step.

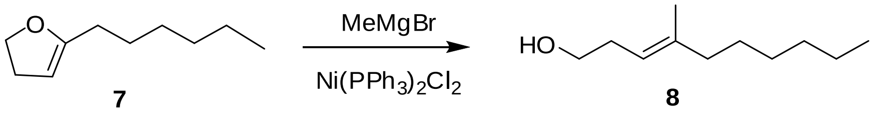

(*E*)-4-methyldec-3-en-1-ol 8

Methylmagnesium bromide solution (68.00 mL of 3 *M* in toluene, 204.00 mmol) was added to a mixed solution of bis(triphenylphosphine)nickel(II) dichloride (2.23 g, 3.40 mmol) and anhydrous toluene under the protection of nitrogen, and then the mixture was stirred at room temperature for 30 min. After compound **7** (14.80 g) was added, the solution was heated to reflux for 1 h.[1] When the reaction was completed based on TLC detection, saturated aqueous ammonium chloride solution (50 mL) was added to quench the reaction, and the product was extracted with ethyl ether (3 × 50 mL). The combined organic phase was washed by saturated sodium chloride solution (3 × 100 mL), dried over sodium sulfate and concentrated under vacuum. The residue was purified using silica gel chromatography (8% ethyl acetate in hexanes) to give a colorless transparent oil (13.00 g, 80% yield for two steps), which was confirmed as (*E*)-4-methyldec-3-en-1-ol **8** by ^{1}H NMR. The NMR spectra of obtained compound **8** matched completely with those for the known product.[1] ^{1}H NMR (300 MHz, $CDCl_3$) δ 5.12 (t, *J* = 7.9 Hz, 1H, C<u>H</u>=C), 3.62 (t, *J* = 6.4 Hz, 2H, -C<u>H2</u>OH), 2.29 (q, *J* = 6.8 Hz, 2H, C<u>H2</u>CH=C), 2.00 (t, *J* = 7.5 Hz, 2H, C<u>H2</u>$(CH_2)_4CH_3$), 1.63 (s, 3H, CH=CC<u>H3</u>), 1.61 (s, 1H, O<u>H</u>), 1.46 –

1.21 (m, 8H, $CH_2(C\underline{H_2})_{\underline{4}}CH_3$), 0.88 (t, *J* = 6.8 Hz, 3H, $CH_2(CH_2)_4C\underline{H_3}$). ^{13}C NMR (75 MHz, $CDCl_3$) δ 139.0, 119.5, 62.4, 39.8, 31.7, 31.5, 28.9, 27.9, 22.6, 16.0, 14.0. HRMS (ESI) $[M+H]^+$ calcd for $C_{11}H_{23}O$, 171.1743; found: 171.1743.

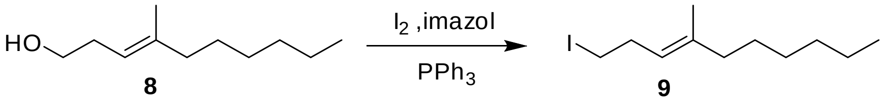

(*E*)-1-Iodo-4-methyldec-3-ene 9

Iodine (10.28 g, 40.50 mmol) was added in batches to a solution of imidazole (4.09 g, 60.00 mmol), triphenylphosphine (12.59 g, 48.00 mmol) indichloromethane (250 mL) at room temperature. Then the mixed solution was allowed to cool to 0 °C under stirring. The compound **8** (6.80g, 40.00 mmol in DCM 40mL) was dropped during 30 min. Then the reaction solution was warmed to room temperature, and stirred for 2 h.[1] After the reaction was completed by TLC detection, the resulting mixture was concentrated to 20 mL under vacuum, and the residue was purified using silica gel chromatography (10% ethyl acetate in hexanes) to give the dark red oil. Then the obtained dark red oil was dissolved in 50 mL of ethyl ether, washed by saturated sodium thiosulfate solution (3 × 50 mL), dried over sodium sulfate, and concentrated under vacuum to give (*E*)-1 -iodo-4-methyldec-3-ene **9** as a colorless transparent oil (9.86 g, 88% yield). The NMR spectra of obtained compound **9** matched completely with those for the known product.[1] ^{1}H NMR (300 MHz, $CDCl_3$) δ 5.09 (t, *J* = 7.1, Hz, 1H, $C\underline{H}=C$), 3.10 (t, *J* = 7.4 Hz, 2H, $C\underline{H_2}I$), 2.57 (q, *J* = 7.3 Hz, 2H, $C\underline{H_2}CH_2I$), 1.97 (t, *J* = 7.4 Hz, 2H, $C\underline{H_2}(CH_2)_4CH_3$), 1.59 (s, 3H, $CH=CC\underline{H_3}$), 1.45 – 1.21 (m, 8H, $CH_2(C\underline{H_2})_{\underline{4}}CH_3$), 0.88 (t, *J* = 6.7 Hz, 3H, $CH_2(CH_2)_4C\underline{H_3}$). ^{13}C NMR (75 MHz, $CDCl_3$) δ 138.3, 122.7, 39.6, 32.3, 31.7, 28.9, 27.7, 22.6, 16.1, 14.1, 5.9. HRMS (APCI) $[M+H]^+$ calcd for $C_{11}H_{22}I$, 281.0761; found: 281.0764.

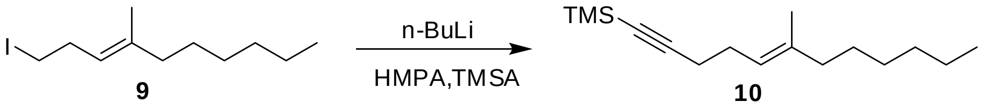

(*E*)-Trimethyl(6-methyldodec-5-en-1-ynyl)silane 10

n-Butyllithium solution (6.7 mL of a 2.4 *M* solution in hexane, 16.08 mmol) was added dropwise to a mixed solution of ethynyltrimethylsilane (1.57 g, 16.00 mmol) and hexamethylphosphoramide (2.86 g, 2.79 mL, 16.00 mmol) in anhydrous THF (15 mL) at -78 °C.[2] After addition, the solution was warmed to -10 °C and maintained for 2 h. Then the solution was allowed to cool to -78 °C again, the compound **9** (3.810 g, 13.6 mmol in THF 5 ml) was added, and the solution was warmed and kept at room temperature for 48 hours. After the reaction was completed by TLC detection, saturated

aqueous ammonium chloride (50 mL) was added to quench the reaction, and the product was extracted with ethyl ether (3 × 50 mL). The combined organic phase was washed by saturated sodium chloride solution (3 × 100 mL), dried over sodium sulfate and concentrated under vacuum to get colorless transparent oil (14.80 g) as compound **10**, which was directly used in the next step.

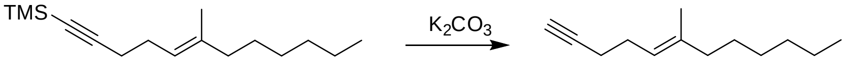

10 **11**

(*E*)-6-Methyldodec-5-en-1-yne 11

Anhydrous potassium carbonate (3.32 g, 24.00 mmol) was added to the solution of compound **10** (3.34 g) in anhydrous methanol (20 mL) and the solution was stirred at room temperature for 3 h. After the reaction was completed by TLC detection, saturated aqueous ammonium chloride solution (50 mL) was added, and the product was extracted with ethyl ether (3 × 50 mL). The combined organic phase was washed by saturated sodium chloride solution (3 × 100 mL), dried over sodium sulfate and concentrated under vacuum. The residue was purified using silica gel chromatography (hexanes) to give (*E*)-6-methyldodec-5-en-1-yne **11** as a colorless transparent oil (1.81 g, 76% yield for two steps). ^{1}H NMR (300 MHz, $CDCl_3$) δ 5.20 – 5.12 (m, 1H, CH=C), 2.28 – 2.13 (m, 4H, $CH(CH_2)_2$), 1.97 (t, *J* = 7.4 Hz, 2H, $CH_2(CH_2)_4CH_3$), 1.91 (t, *J* = 2.4 Hz, 1H, CH≡C), 1.60 (s, 3H, $CH{=}CCH_3$), 1.44 – 1.22 (m, 8H, $CH_2(CH_2)_4CH_3$), 0.88 (t, *J* = 6.8 Hz, 3H, $CH_2(CH_2)_4CH_3$). ^{13}C NMR ($CDCl_3$,75 MHz) δ 136.9, 122.2, 84.3, 68.0, 39.6, 31.8, 28.9, 27.8, 27.2, 22.6, 18.9, 15.9, 14.0. HRMS (APCI) calcd $[M+H]^+$ for $C_{13}H_{23}$, 179.1794; found: 179.1796.

Preparation of Garner Aldehyde

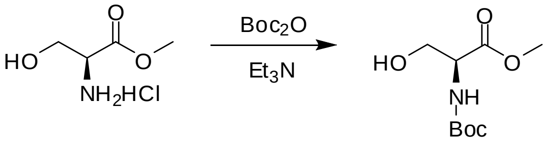

12

Methyl(2*S*)-2-(*tert*-butoxycarbonylamino)-3-hydroxypropanoate 12

Triethylamine (193.45 mL, 1386.00 mmol) was slowly added to a mixed solution of L-serine methyl ester hydrochloride (98.29 g, 630.00 mmol) in DCM (1 L) at -5 °C in 1 h, and di-*tert*-butyl dicarbonate (154.31 mL, 693.00 mmol) was dropped in 2 h, then mixed solution was warmed to room temperature for overnight. After the reaction was completed by TLC detection, the reaction

mixture was concentrated under vacuum, and the residue was diluted by ethyl acteate (300 mL). The organic phase was washed by saturated sodium bicarbonate solution (3 × 100 mL), dried over sodium sulfate and concentrated under vacuum to give methyl(*S*)-2-(*tert*-butoxycarbonylamino) -3-hydroxypropanoate **12** as a colorless transparent oil (131.67 g, 95% yield). $[\alpha]_D$ = +9.8 (*c* 0.60, $CHCl_3$). ^{1}H NMR (300 MHz, $CDCl_3$) δ 5.58 (d, *J* = 7.3 Hz, 1H, CHNHBoc), 4.41 – 4.27 (m, 1H, CHNHBoc), 3.88 (d, *J* = 16.7 Hz, 2H, CH_2OH), 3.74 (s, 3H, $COOCH_3$), 3.11 (s, 1H, CHOH), 1.41 (s, 9H, $O{=}COC(CH_3)_3$). ^{13}C NMR (75 MHz, $CDCl_3$) δ 171.4, 155.8, 80.2, 63.2, 55.7, 55.5, 28.2. HRMS (ESI) $[M+Na]^+$ calcd for $C_9H_{17}NNaO_5$, 242.0999; found: 242.1002.

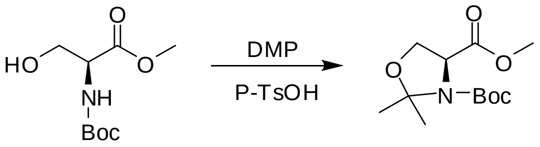

12 **13**

3-*tert*-Butyl 4-methyl(4*S*)-2,2-dimethyloxazolidine-3,4-dicarboxylate 13

2,2-Dimethoxypropane (78.35 mL, 639.00 mmol) was added to a solution of compound **12** (46.50 g, 213.00 mmol) in DCM (500 mL) at 0 °C in 1 h, and *p*-toluenesulfonic acid monohydrate (0.50 g) was added. Then the solution was allowed to warm to room temperature for overnight. After the reaction was completed by TLC detection, the resulting mixture was concentrated under vacuum, and was diluted by ethyl acetate (300 mL). The organic phase was washed by saturated sodium bicarbonate solution (3 × 100 mL), dried over sodium sulfate and concentrated under vacuum to get the colorless transparent oil (44.10 g), which was directly used in the next step.

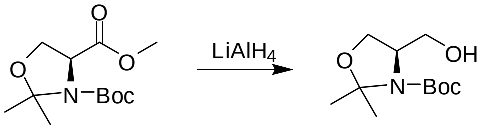

13 **14**

***tert*-Butyl (4*R*)-4-(hydroxymethyl)-2,2-dimethyloxazolidine-3-carboxylate 14**

Compound **13** (20.21 g, 77.00 mmol in anhydrous ethyl ether 150 mL) was slowly dropped into a solution of lithium aluminum hydride (8.83g, 230 mmol) in anhydrous ethyl ether (300 mL) at -5 °C and stirred for 3 h. Then the mixed solution was warmed to room temperature, and stirred for 5 h. After the reaction was completed by TLC detection, water (23 mL), 50% sodium hydroxide solution (23 mL), water (60 mL) were in order added to the solution at -5 °C, and stirred for 1 h. After the solution was filtered, the organic phase was washed by saturated sodium chloride solution (3 × 100

mL), dried over sodium sulfate and concentrated under vacuum. The residue was purified using silica gel chromatography (10% ethyl acetate in hexanes) to give *tert*-butyl(*R*)-4-(hydroxymethyl) -2,2-dimethyloxazolidine-3-carboxylate **14** as a colorless transparent oil (11.74 g, 66% yield). $[\alpha]_D$ = -23.2 (*c* 0.73, $CHCl_3$). ^{1}H NMR (300 MHz, $CDCl_3$) δ 4.07-3.97 (m, 2H, CHNBoc, CH_aH_bO), 3.87 – 3.67 (m, 2H, CH_aH_bO,CH_aH_bOH), 3.66 – 3.52 (m, 1H,CH_aH_bOH), 1.62 – 1.41 (m, 15H, C $(CH_3)_2$,O=COC $(CH_3)_3$). ^{13}C NMR (75 MHz, $CDCl_3$) δ 153.9, 94.0, 81.0, 65.2, 64.8, 59.4, 28.3, 27.1, 24.5. HRMS (ESI) $[M+Na]^+$ calcd for $C_{11}H_{21}NNaO_4$, 254.1363; found: 254.1364.

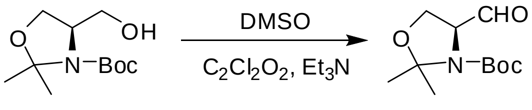

14 **15**

tert-Butyl (4*S*)-4-formyl-2,2-dimethyloxazolidine-3-carboxylate (Garner aldehyde) 15

Dimethyl sulfoxide (1.23 mL,17.40 mmol) was slowly dropped into a solution of oxalyl chloride (1.00 mL, 10.44 mmol) in DCM (20 mL) at -78 °C, and stirred for 5 min. Compound **14** (2.00 g, 8.70 mmol in DCM 5 mL) was added, and stirred for 15 min. Then, triethylamine (4.85 mL, 34.80 mmol) was added, and the solution was warmed to room temperature in 1 h. After the reaction was completed by TLC detection, saturated sodium bicarbonate solution (50 mL) was added to quench the reaction, and the product was extracted with ethyl ether (3 × 20 mL). The combined organic phase was to washed in order by sodium hydrogen sulfate (3 × 50 mL), saturated sodium bicarbonate solution (3 × 50 mL), water (3 × 50 mL), dried over sodium sulfate and concentrated under vacuum to obtain colorless transparent oil (1.63 g), which was directly used in the next step.

Preparation of Sphingosine Fragment

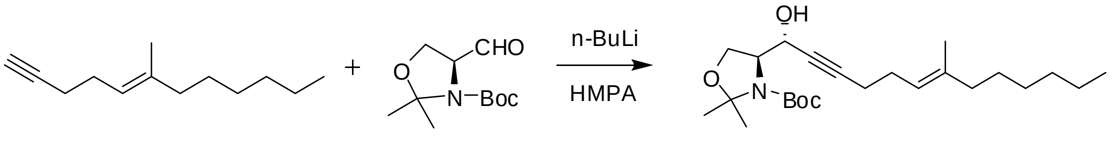

11 **15** **16**

tert-Butyl

(4*S*)-4-((1*R*,6*E*)-1-hydroxy-7-methyltridec-6-en-2-ynyl)-2,2-dimethyloxazolidine-3-carboxylate 16

n-Butyllithium solution (8.30 mL of a 2.4 *M* solution in hexane, 19.92 mmol) was added slowly to a mixed solution of compound **11** (3.50 g, 20.00 mmol) in anhydrous THF (80 mL) at -78 °C,[3] and the solution was warmed to -30 °C and stirred for 2 h. The solution was cooled again to -78 °C, and

hexamethylphosphoramide (3.59 g, 3.48 mL, 20.00 mmol) and the compound **15** (2.70 g, 12.00 mmol in THF 10 ml)was added and stirred for 10 min, and then the solution was allowed to warmed to-30 °C and maintained for 2 h. After the reaction was completed by TLC detection, saturated aqueous ammonium chloride (50 mL) was added to quench the reaction, and the product was extracted with ethyl ether (3 × 50 mL).The combined organic phase was washed by saturated sodium chloride solution (3 × 100 mL), dried over sodium sulfate and concentrated under vacuum. The residue was purified using silica gel chromatography (8% ethyl acetate in hexanes) to give *tert*-butyl (*4S*)-4-((*1R,6E*)-1-hydroxy-7-methyltridec-6-en-2-ynyl)-2,2-dimethyloxazolidine-3-carboxylate **16** as a colorless transparent oil (4.10 g, 83% yield). $[\alpha]_D$ = -76.4 (*c* 0.56, $CHCl_3$). ^1H NMR (300 MHz, $CDCl_3$) δ 5.18 – 5.07 (m, 1H, $C\underline{H}{=}CCH_3$), 4.77 – 4.45 (m, 2H, $C\underline{H}NBoc$, $C\underline{H_a}H_bO$), 4.19 – 3.85 (m, 3H, $CH_a\underline{H_b}O$, $C\underline{H}O\underline{H}$), 2.31 – 2.10 (m, 4H, $C{\equiv}CC\underline{H_2}C\underline{H_2}CHCH_3$), 1.96 (t, *J* = 7.4 Hz, 2H, $CH{=}CCH_3C\underline{H_2}$), 1.59 (s, 6H, $C(C\underline{H_3})_2$), 1.50 (s, 12H, $CHC{=}C\underline{H_3}CH_2$, $O{=}COC(C\underline{H_3})_3$), 1.46 – 1.17 (m, 8H, $CH_3(C\underline{H_2})_4CH_3$), 0.88 (t, *J* = 6.7 Hz, 3H, $(CH_2)_4C\underline{H_3}$). ^{13}C NMR (75 MHz, $CDCl_3$) δ 154.1, 137.0, 122.3, 94.9, 86.5, 81.1, 65.0, 64.0, 62.8, 39.6, 31.7, 29.7, 29.0, 28.4, 27.9, 27.3, 25.8, 25.4, 22.6, 19.3, 16.0, 14.1. HRMS (ESI) $[M+Na]^+$ calcd for $C_{24}H_{41}NNaO_4$, 430.2928; found: 430.2926.

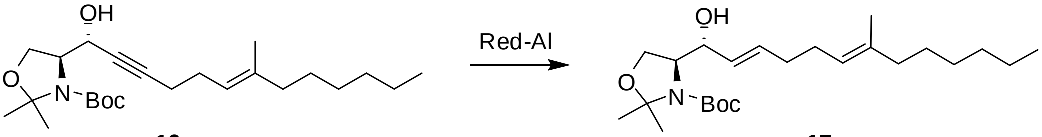

16 **17**

***tert*-Butyl (4*S*)-4-((1*R*,2*E*,6*E*)-1-hydroxy-7-methyltrideca-2,6-dienyl)-2,2-dimethyloxazolidine-3-carboxylate 17**

Bis(2-methoxyethoxy)aluminum hydride solution (2.42 mL,7.98 mmol, 70% in toluene) was slowly added to a solution of compound **16** (1.31 g, 3.19 mmol) and anhydrous ethyl ether (30 mL) at 0 °C under the protection of nitrogen, and the solution was held for 10 min followed by be warmed to room temperature for overnight. After the reaction was completed by TLC detection, saturated aqueous ammonium chloride solution (1 mL) was added to quench the reaction, and the product was extracted with ethyl ether (3 × 50 mL).The combined organic phase was washed by saturated sodium chloride solution (3 × 100 mL), dried over sodium sulfate and concentrated under vacuum.

The residue was purified using silica gel chromatography (6% ethyl acetate in hexanes) to give *tert*-butyl(4*S*)-4-((1*R,*2*E,*6*E*)-1-hydroxy-7-methyltrideca-2,6-dienyl)-2,2-dimethyloxazolidine-3-carbo xylate **17** as a colorless transparent oil (1.25 g, 96% yield). $[\alpha]_D$ = -23.1 (*c* 0.65, $CHCl_3$). ^1H NMR (300 MHz, $CDCl_3$) δ 5.83 – 6.67 (m, 1H, CHOHCH=$CHCH_2$), 5.53 – 5.39 (m, 1H,CHOHCH=$CHCH_2$), 5.16 – 5.07 (m, 1H, CH=CCH_3), 4.31 – 3.78 (m, 5H, CHNBoc, CH_2O, CHOH), 2.16 – 2.00 (m, 4H, CH=$CHCH_2CH_2CH$=CCH_3), 1.95 (t, *J* = 7.4 Hz, 2H, CH=CCH_3CH_2), 1.63 – 1.45 (m, 18H, $C(CH_3)_2$, CH=CCH_3CH_2, O=$COC(CH_3)_3$), 1.39 – 1.20 (m, 8H, $(CH_2)_4CH_3$), 0.88 (t, *J* = 6.8 Hz, 3H, $(CH_2)_4CH_3$).;^{13}C NMR (75 MHz, $CDCl_3$) δ 154.1, 135.8, 132.9, 128.7, 123.3, 94.4, 81.1, 65.2, 63.8, 62.2, 39.6, 32.6, 31.7, 29.6, 28.9, 28.3, 27.9, 27.6, 26.2, 22.6, 19.2, 15.9, 14.0. HRMS (ESI) $[M+Na]^+$ calcd for $C_{24}H_{43}NNaO_4$, 432.3084; found: 432.3087.

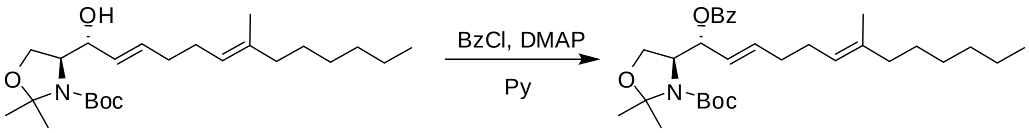

17 **18**

tert-Butyl (4*S*)-4-((1*R,*2*E,*6*E*)-1-(benzoyloxy)-7-methyltrideca-2,6-dienyl)-2,2-dimethyloxazo-lidine-3-carboxy late 18

Benzoyl chloride (0.47 mL, 4.00 mmol) was slowly dropped into a solution of compound **17** (0.82 g, 2.00 mmol), anhydrous pyridine (10 mL) and 4-(dimethylamino)pyridine (0.01 g) at 0 °C. Then the solution was warmed to room temperature, and stirred for overnight.[4] After the reaction was completed by TLC detection, ice water (100 mL) was added to quench the reaction, and the product was extracted with ethyl ether (3 × 50 mL). The combined organic phase was washed by ice hydrochloric acid (3 × 50 mL) and saturated sodium chloride solution (3 × 100 mL), dried over sodium sulfate and concentrated under vacuum. The residue was purified using silica gel chromatography (2% ethyl acetate in hexanes) to give *tert*-butyl(4*S*)-4-((1*R,*2*E,*6*E*)-1-(benzoyloxy) -7-methyltrideca-2,6-dienyl)-2,2-dimethyl-oxazolidine-3-carboxylate **18** as a colorless transparent oil (0.93 g, 91% yield). $[\alpha]_D$ = -28.1 (*c* 0.67, $CHCl_3$). ^1H NMR (300 MHz, $CDCl_3$) δ 8.09 (d, *J* = 7.1 Hz, 2H, Ar-H), 7.54 (t, *J* = 7.4 Hz, 1H, Ar-H), 7.42 (t, *J* = 7.5 Hz, 2H, Ar-H), 5.78 – 5.88 (m, 2H, CHOBzCH=$CHCH_2$), 5.55 – 5.37 (m, 1H, CHOBzCH=$CHCH_2$), 5.12 – 5.00 (m, 1H, CH=CCH_3), 4.26 – 3.94 (m, 3H CHNBoc, CH_2O), 2.15 – 2.00 (m, 4H,CH=$CHCH_2CH_2CHCH_3$), 1.90 (t, *J* = 7.3 Hz, 2H,

$CH{=}CCH_3C\underline{H_2}$), 1.83 – 1.37 (m, 18H, $C(C\underline{H_3})_2$, $CH{=}CC\underline{H_3}CH_2$, $O{=}COC(C\underline{H_3})_3$), 1.36 – 1.13 (m, 8H, $(C\underline{H_2})_4CH_3$), 0.86 (t, J = 6.8 Hz, 3H, $(CH_2)_4C\underline{H_3}$). ^{13}C NMR (75 MHz, $CDCl_3$) δ 165.5, 151.8, 136.2, 135.1, 132.9, 129.9, 128.3, 125.4, 122.9, 94.6, 80.2, 74.1, 63.7, 60.0, 39.6, 32.6, 31.7, 29.0, 28.4, 27.9, 27.3, 27.1, 26.2, 24.4, 23.0, 22.6, 16.0, 14.1. HRMS (ESI) $[M+Na]^+$ calcd for $C_{31}H_{47}NNaO_5$, 536.3346; found: 536.3351.

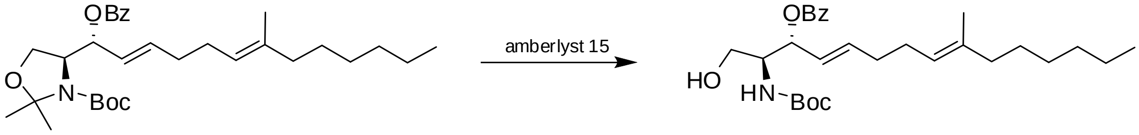

18 **19**

(2*S*,3*R*,4*E*,8*E*)-2-(*tert*-butoxycarbonylamino)-1-hydroxy-9-methylpentadeca-4,8-dien-3-yl benzoate 19

Amberlyst 15 (1.00 g) was added to a solution of compound **18** (0.77 g, 1.50 mmol) and anhydrous methanol at room temperature and stirred for 72 h.[5] After the reaction was completed by TLC detection, the solid was removed through filtration, and the filtrate was concentrated under vacuum. The residue was purified using silica gel chromatography (20% ethyl acetate in hexanes) to give (2*S*,3*R*,4*E*,8*E*)-2-(*tert*-butoxycarbonylamino)-1-hydroxy-9-methylpentadeca-4,8-dien-3-ylbenzoate **19** as a colorless transparent oil (0.53 g, 74% yield). $[\alpha]_D$ = -31.7 (c 0.87, $CHCl_3$). ^1H NMR (300 MHz, $CDCl_3$) δ 8.04 (d, J = 7.3 Hz, 2H, Ar-$\underline{H}$), 7.56 (t, J = 7.4 Hz, 1H, Ar-$\underline{H}$), 7.43 (t, J = 7.5 Hz, 2H, Ar-$\underline{H}$), 5.98 – 5.78 (m, 1H, $CHOBzC\underline{H}{=}CHCH_2$), 5.69 – 5.50 (m, 2H, $C\underline{H}OBzCH{=}C\underline{H}CH_2$), 5.22 – 5.00 (m, 2H, $C\underline{H}{=}CCH_3$, $CH_2O\underline{H}$), 4.04– 3.88 (m, 1H, $C\underline{H}NBoc$), 3.78 – 3.61 (m, 2H, $C\underline{H_2}O$), 2.14 – 2.01 (m, 4H, $CH{=}CHC\underline{H_2}C\underline{H_2}CHCH_3$), 1.96 – 1.88 (m, 2H, $CHCCH_3C\underline{H_2}$), 1.59 – 1.38 (m, 12H, $CH{=}CC\underline{H_3}CH_2$, $O{=}COC(C\underline{H_3})_3$), 1.36 – 1.19 (m, 8H, $(C\underline{H_2})_4CH_3$), 0.87 (t, J = 6.8 Hz, 3H, $(CH_2)_4C\underline{H_3}$). ^{13}C NMR (75 MHz, $CDCl_3$) δ 166.0, 155.8, 137.0, 136.5, 136.1, 133.1, 129.7, 128.3, 125.1, 124.8, 123.0, 79.6, 74.7, 61.7, 54.6, 39.6, 32.5, 31.7, 31.5, 29.4, 28.9, 28.3, 27.8, 27.2, 27.1, 22.6, 15.9, 14.0. HRMS (ESI) $[M+Na]^+$ calcd for $C_{28}H_{43}NNaO_5$, 496.3033; found: 496.3030.

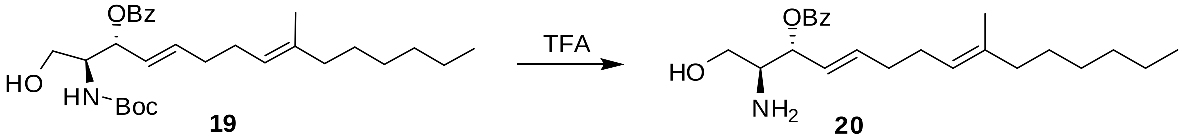

19 **20**

(2*S*,3*R*,4*E*,8*E*)-2-Amino-1-hydroxy-9-methylpentadeca-4,8-dien-3-yl benzoate 20

Trifluoroacetic acid (10.00 mL) was added dropwise to a solution of compound **19** (473 mg, 1.00 mmol) and anhydrous DCM (10 mL) at 0 °C and the solution was stirred at 0 °C for 2 h. After the

reaction was completed by TLC detection, anhydrous toluene (10 mL) was added into the reaction solution, and concentrated under vacuum to get colorless transparent oil, which was directly used in the next step.

Preparation of Glucose Hydroxyl Compound

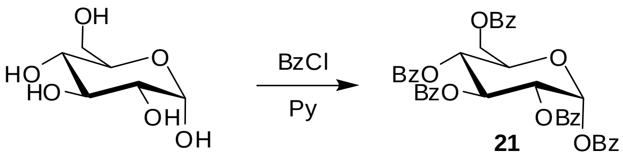

α-D-Glucopyranose pentabenzoate21

α-D-Glucose (2.00 g, 11.12 mmol) in pyridine (24.00 mL) was cooled to 0 °C, benzoyl chloride (4.00 mL, 69.00 mmol) was added dropwise in 10 min, and then the solution was warmed to room temperature and was stirred over 16 h. After the reaction was completed by TLC detection, the solution mixture was diluted with ethyl acetate, washed with hydrochloric acid (1 *M*) (3 × 50 mL), water (3 × 50 mL), dried over sodium sulfate, and concentrated under vacuum.[6] Recrystallizing the residue with solvents acetone and water gave the compound **21** as a white solid (5.55 g, 71% yield). $[\alpha]_D$ = +142.9 (*c* 0.55, $CHCl_3$). ^{1}H NMR (300 MHz, $CDCl_3$) δ 8.21 – 8.14 (m, 2H, Ar-H), 8.07 – 8.00 (m, 2H, Ar-H), 7.98 – 7.83 (m, 6H, Ar-H), 7.67 (t, *J* = 7.4 Hz, 1H, Ar-H), 7.61 – 7.27 (m, 14H, Ar-H), 6.85 (d, *J* = 3.8 Hz, 1H, H-1), 6.32 (t, *J* = 10.0 Hz, 1H, H-3), 5.87 (t, *J* = 9.8 Hz, 1H, H-4), 5.68 (dd, *J* = 10.3, 3.8 Hz, 1H, H-2), 4.67-4.58 (m, 2H, H-5,H-6a), 4.48 (dd, *J* = 13.0, 5.1 Hz, 1H, H-6b). ^{13}C NMR (75 MHz, $CDCl_3$) δ 165.8, 165.7, 165.1, 165.0, 164.2, 133.7, 133.3, 133.1, 132.9, 129.9, 129.8, 129.6, 129.5, 129.3, 128.8, 128.6, 128.5, 128.4, 128.2, 128.1, 89.9, 70.4, 68.8, 62.3.HRMS (ESI) $[M+NH_4]^+$ calcd for $C_{41}H_{36}NO_{12}$, 718.2883; found: 718.2890.

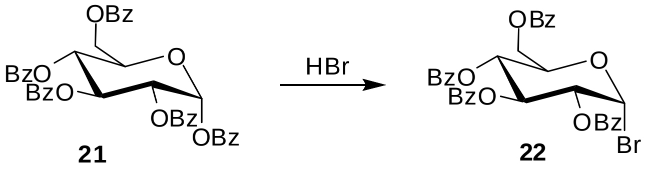

2,3,4,6-Tetra-*O*- benzoyl-α-D-glucopyranosyl bromide 22

Compound **21** (4.00 g, 5.71 mmol) was dissolved in DCM (20 mL) and cooled to 0 °C. To this solution hydrobromic acid (10 mL, 33% in acetic acid) was added, and was stirred at room temperature for 6 h. After the reaction was completed by TLC detection, the mixed solution was diluted with diethyl

ether, washed with water (3 × 50 mL), saturated sodium bicarbonate solution (3 × 50 mL), water (3 × 50 mL), dried over sodium sulfate, and concentrated under vacuum to obtain colorless transparent oil,[6] which was directly used in the next step.

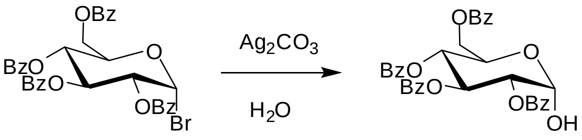

22 23

2,3,4,6-Tetra-*O*-benzoyl-α-D-glucopyranose 23

Compound **22** was dissolved in acetone (100 mL) and water (2 mL), to this solution silver carbonate (0.79 g, 2.86 mmol) was added and the solution was stirred for 16 h at room temperature. After the reaction was completed by TLC detection, the mixture solution was filtered through Celite, and the solid was rinsed with DCM. Organic phase was combined together, dried over sodium sulfate and concentrated under vacuum.[6] The residue was purified using silica gel chromatography (25% ethyl acetate in hexanes) to give the compound **23** as a white solid (2.83 g, 83% yield for two steps). $[\alpha]_D$ = +111.4 (c 0.55, $CHCl_3$). ^{1}H NMR (300 MHz, $CDCl_3$) δ 8.14 – 7.84 (m, 8H,Ar-H), 7.63 – 7.29 (m, 12H,Ar-H), 6.28 (t, *J* = 9.9 Hz, 1H, H-3), 5.79 – 5.78 (m, 1H, H-4), 5.34 (dd, *J* = 10.2, 3.3 Hz, 1H, H-2), 4.78 – 4.59 (m, 2H, H-5, H-6a), 4.46 (dd, *J* = 12.4, 4.5 Hz, 1H, H-6b), 3.62 (d, *J* = 3.4 Hz, 1H, H-1), 1.72 (s, 1H, OH). ^{13}C NMR (75 MHz, $CDCl_3$) δ 166.5, 166.0, 165.9, 165.3, 133.4, 133.1, 129.8, 129.7, 128.4, 128.3, 90.4, 72.3, 70.3, 69.6, 67.6, 62.9. HRMS (ESI) $[M+NH_4]^+$ calcd for $C_{34}H_{32}NO_{10}$, 614.2537; found: 614.2544.

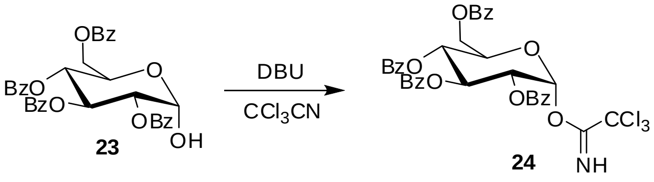

23 24

2,3,4,6-Tetra-*O*-benzoyl-1-(2,2,2-trichloroethanimidate)-α-D-glucopyranoside 24

Compound **23** (2.22 g, 3.73 mmol) was dissolved in DCM (80 mL) and cooled to 0 °C. Trichloroacetonitrile (3.74 mL, 37.30 mmol) and 1,8-diazabicyclo[5.4.0]undec-7-ene (5 drops) were added, and the solution was stirred for 4 h at 0 °C. After the reaction was completed by TLC detection, the solution was concentrated under vacuum to 4 mL.[6] The residue was purified using silica gel chromatography (20% ethyl acetate in hexanes) to give compound **24** as a colorless oil (1.88 g, 68% yield). $[\alpha]_D$ = +95.7 (*c* 0.59, $CHCl_3$). ^{1}H NMR (300 MHz, $CDCl_3$) δ 8.65 (s, 1H, NH), 8.09 –

8.03 (m, 2H, Ar-H), 8.01 – 7.93 (m, 4H, Ar-H), 7.93 – 7.86 (m, 2H, Ar-H), 7.63 – 7.29 (m, 12H, Ar-H), 6.86 (d, *J* = 3.7 Hz, 1H, H-1), 6.30 (t, *J* = 10.0 Hz, 1H, H-3), 5.84 (t, *J* = 9.8 Hz, 1H, H-4), 5.64 (dd, *J* = 10.2, 3.7 Hz, 1H, H-2), 4.73 – 4.60 (m, 2H, H-5, H-6a), 4.54 – 4.46 (m, 1H, H-6b). ^{13}C NMR (75 MHz, $CDCl_3$) δ 166.0, 165.6, 165.3, 165.2, 160.4, 133.5, 133.3, 133.1, 139.9, 129.7, 129.6, 128.4, 128.3, 93.1, 70.7, 70.2, 68.7, 62.5. HRMS (ESI) $[M+Na]^+$ calcd for $C_{36}H_{28}Cl_3NNaO_{10}$, 764.0642; found: 764.0641.

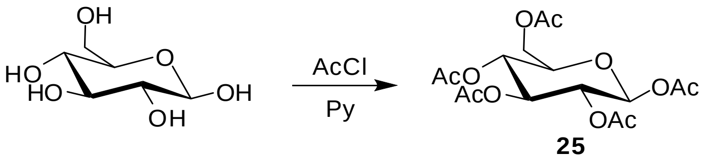

25

β-D-Glucopyranose pentaacetate 25

β-D-Glucose 2.00 g (11.12 mmol) in pyridine (24.00 mL) was cooled to 0 °C, to this solution acetyl chloride (3.26mL,69.00 mmol) was added dropwise in 10 min, and the solution was warmed to room temperature and stirred over 16 h. After the reaction was completed by TLC detection, the solution mixture was diluted with ethyl acetate, washed with hydrochloric acid (1 *M*) (3 × 50 mL), water (3 × 50 mL), dried over sodium sulfate and concentrated under vacuum.[6] Recrystallizing residue with acetone/water gave the compound **25** as a white solid (3.09 g, 73% yield). $[\alpha]_D$ = +5.1 (*c* 0.74, $CHCl_3$). ^{1}H NMR (300 MHz, $CDCl_3$) δ 5.72 (d, *J* = 8.2 Hz, 1H, H-1), 5.31 – 5.21 (m, 1H, H-3), 5.19 – 5.08 (m, 2H, H-4, H-2), 4.30 (dd, *J* = 12.5, 4.5 Hz, 1H, H-6a), 4.11 (dd, *J* = 12.5, 2.2 Hz, 1H, H-6b), 3.84 (ddd, *J* = 9.8, 4.5, 2.2 Hz, 1H, H-5), 2.16 – 1.99 (m, 15H,OC=OCH3). ^{13}C NMR (75 MHz, $CDCl_3$) δ 170.5, 170.0, 169.3, 169.2, 168.9, 91.7, 72.8, 72.7, 70.2, 67.8, 61.5, 20.8, 20.7, 20.5.HRMS (ESI) $[M+Na]^+$ calcd for $C_{16}H_{22}NaO_{11}$, 413.1054; found: 413.1052.

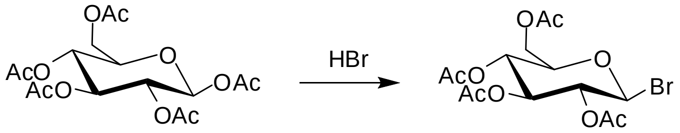

25 26

2,3,4,6-Tetra-*O*-acetyl- β-D-glucopyranosyl bromide 26

Compound **25** (2.24 g, 5.71 mmol) was dissolved in DCM (20 mL) and cooled to 0 °C. To this solution hydrobromic acid (10 mL, 33% in acetic acid) was added and the solution was stirred at room temperature for 6 h. After the reaction was completed by TLC detection, the mixed solution was diluted with diethyl ether, and washed in order with water (3 × 50 mL), saturated sodium

bicarbonate solution (3 × 50 mL) and water (3 × 50 mL). The obtained organic phase was dried over sodium sulfate and concentrated under vacuum[6] to obtain colorless transparent oil, which was directly used in the next step.

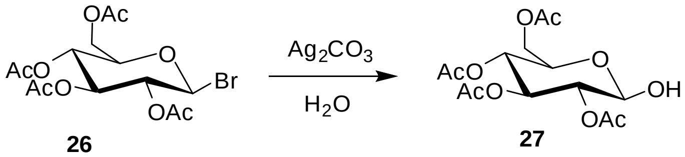

2,3,4,6-Tetra-*O*-acetyl-β-D-glucopyranose 27

Compound **26** was dissolved in acetone (100 mL) and water (2 mL), to this solution silver carbonate (0.79 g, 2.86 mmol) was added and then the mixture was stirred for 16 h at room temperature. After the reaction was completed by TLC detection, the reaction mixture was filtered through Celite, and solid was rinsed with DCM. Organic phase was combined together, dried over sodium sulfate and concentrated under vacuum.[6] The residue was purified using silica gel chromatography (25% ethyl acetate in hexanes) to give the compound **27** as a white solid (1.65 g, 84% yield for two steps). $[\alpha]_D$ = +9.2 (*c* 0.60, $CHCl_3$). ^1H NMR (300 MHz, $CDCl_3$) δ 5.25 (t, *J* = 9.5 Hz, 1H, H-3), 5.09 (t, *J* = 9.7 Hz, 1H, H-4), 4.91 (dd, *J* = 9.6, 8.1 Hz, 1H, H-2), 4.76 (t, *J* = 8.4 Hz, 1H, H-1), 4.29 – 4.06 (m, 3H, H-6, OH), 3.77 (ddd, *J* = 10.1, 4.8, 2.4 Hz, 1H, H-5), 2.15 – 1.96 (m, 12H,OC=OCH3). ^{13}C NMR (75 MHz, $CDCl_3$) δ 170.8, 170.6, 170.1, 169.5, 95.4, 73.1, 72.3, 72.0, 68.4, 62.0, 20.6, 20.5. HRMS (ESI) $[M+Na]^+$ calcd for $C_{14}H_{20}NaO_{10}$, 371.0949; found: 371.0952.

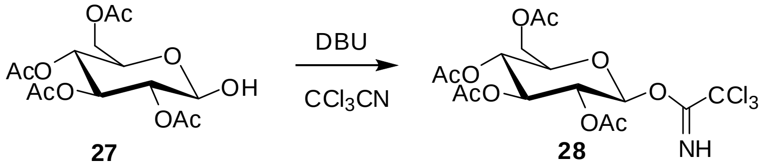

2,3,4,6-Tetra-*O*-acetyl- 1-(2,2,2-trichloroethanimidate)-β-D-glucopyranoside 28

Compound **27** (1.30 g, 3.73 mmol) was dissolved in DCM (80 mL) and cooled to 0 °C. Trichloroacetonitrile (3.74 mL, 37.3 mmol) and 1,8-diazabicyclo[5.4.0]undec-7-ene (5 drops) were added, and the mixture was stirred for 4 h at 0 °C. After the reaction was completed by TLC detection, the solution was concentrated under vacuum to 4 mL.[6] The residue was purified using silica gel chromatography (20% ethyl acetate in hexanes) to give compound **28** as a colorless oil (1.25 g, 67% yield). $[\alpha]_D$ = +7.9 (*c* 0.83, $CHCl_3$). ^1H NMR (300 MHz, $CDCl_3$) δ 8.72 (s, 1H, NH), 5.86 (d, *J* = 7.9 Hz, 1H,H-1), 5.37 – 5.13 (m, 3H, H-3, H-4, H-2), 4.30 (dd, *J* = 12.5, 4.4 Hz, 1H, H-6a), 4.14 (dd, *J*

= 12.5, 2.4 Hz, 1H, H-6b), 3.89 (ddd, *J* = 9.5, 4.3, 2.4 Hz, 1H, H-5), 2.20 – 1.90 (m, 12H,OC=OCH_3). ^{13}C NMR (75 MHz, CDCl$_3$) δ 170.5, 170.0, 169.3, 168.9, 160.8, 95.5, 72.6, 72.5, 70.1, 67.8, 61.5, 20.6, 20.5, 20.4. HRMS (ESI) [M+Na]$^+$ calcd for $C_{16}H_{20}Cl_3NNaO_{10}$, 514.0045; found: 514.0050.

Preparation of α-Hydroxyl-β,γ-Unsaturated Acid

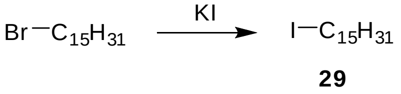

1-Iodopentadecane 29

Potassium iodide (25.21 g, 150.33 mmol) was slowly added to a solution of 1-bromopentadecane (15.20 g, 50.11 mmol) in acetone (500 mL) at room temperature, and the solution was heated to reflux for 48 h. After the reaction was completed by TLC detection, saturated aqueous ammonium chloride (50 mL) was added to quench the reaction, and the product was extracted with ethyl ether (3 × 50 mL).The combined organic phase was washed by saturated sodium chloride solution (3 × 100 mL), dried over sodium sulfate and concentrated under vacuum. The residue was purified using silica gel chromatography (hexanes) to give 1-iodopentadecane **29** as a colorless transparent oil (16.60 g, 98% yield). ^{1}H NMR (300 MHz, CDCl$_3$) δ 3.15 (t, *J* = 7.1 Hz, 2H, CH_2I), 1.91 – 1.73 (m, 2H, CH_2CH$_2$I), 1.50 – 1.14 (m, 24H, (CH_2)$_{12}$), 0.88 (t, *J* = 6.6 Hz, 3H, CH_3). ^{13}C NMR (75 MHz, CDCl$_3$) δ 33.6, 31.9, 30.5, 29.7, 29.6, 29.6, 29.5, 29.4, 29.3, 28.5, 22.6, 14.1, 6.4. GC-MS (ESI) [M]$^+$ calcd for $C_{15}H_{31}I$, 338; found: 338.

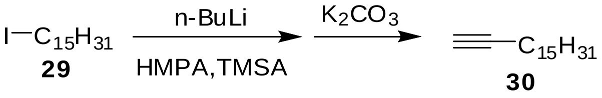

Heptadec-1-yne 30

n-Butyllithium solution (26.15 mL of a 2.4 *M* solution in hexane, 62.76 mmol) was slowly dropped to a mixed solution of ethynyltrimethylsilane (9.05 mL, 62.76 mmol), anhydrous THF (50 mL) andhexamethylphosphoramide (10.92 mL, 62.76 mmol) at -78 °C. After dropping, the solution was warmed to -10 °C and maintained for 2 h. Then the solution was allowed to cool again to -78 °C, and the compound **29** (16.3 g, 56.21 mmol in THF 10 ml) was added. The solution was warmed to room temperature and maintained for 48 hours. After the reaction was completed by TLC detection, saturated aqueous ammonium chloride (50 mL) was added to quench the reaction, and the product was extracted with ethyl ether (3 × 50 mL).The combined organic phase was washed by saturated

sodium chloride solution (3 × 100 mL), dried over sodium sulfate and concentrated under vacuum to get colorless transparent oil (17.31 g).

The above colorless transparent oil was dissolved in anhydrous methanol (50 mL), and anhydrous potassium carbonate (23.31 g, 168.89 mmol) was added, and the mixture was stirred at room temperature for 3 h. After the reaction was completed by TLC detection, saturated aqueous ammonium chloride solution (50 mL) was added to quench the reaction, and the product was extracted with ethyl ether (3 × 50 mL). The combined organic phase was washed by saturated sodium chloride solution (3 × 100 mL), dried over sodium sulfate and concentrated under vacuum. The residue was purified using silica gel chromatography (hexanes) to give heptadec-1-yne **30** as a colorless transparent oil (13.00 g, 98% yield for two steps). ^{1}H NMR (300 MHz, $CDCl_3$) δ 2.18 (td, *J* = 7.0, 2.6 Hz, 2H, $CH_2C{\equiv}CH$), 1.93 (t, *J* = 2.6 Hz, 1H, $C{\equiv}CH$), 1.61 – 1.46 (m, 2H, CH_2CH_2CCH), 1.46 – 1.16 (m, 24H, $(CH_2)_{12}$), 0.88 (t, *J* = 6.7 Hz, 3H, CH_3). ^{13}C NMR (75 MHz, $CDCl_3$) δ 84.8, 68.0, 31.9, 29.7, 29.6, 29.5, 29.4, 29.1, 28.8, 28.5, 22.7, 18.4, 14.1. GC-MS (ESI) $[M]^+$ calcd for $C_{17}H_{32}$, 236; found: 236.

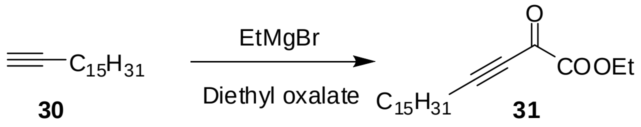

Ethyl 2-oxononadec-3-ynoate 31

Ethylmagnesium bromide solution (6.10 mL of 3 *M* in toluene, 18.30 mmol) was added to a mixed solution of the compound **30** (3.90 g, 16.5 mmol) and anhydrous ethyl ether (50 mL), and the solution was heated to reflux for 6 h. Diethyl oxalate (2.68 mL, 18.30 mmol) in anhydrous ethyl ether (20 mL) was slowly added to this reaction solution at -30 °C, and the solution was stirred for 2 h after addition. When the reaction was completed by TLC detection, saturated aqueous ammonium chloride solution (50 mL) was added to quench the reaction, and the product was extracted with ethyl ether (3 × 50 mL). The combined organic phase was washed by saturated sodium chloride solution (3 × 100 mL), dried over sodium sulfate and concentrated under vacuum. The residue was purified using silica gel chromatography (5% ethyl acetate in hexanes) to give ethyl 2-oxononadec-3-ynoate **31** as a colorless transparent oil (4.44 g, 80% yield). ^{1}H NMR (300 MHz, $CDCl_3$) δ 4.34 (q, *J* = 7.1 Hz, 2H, CH_3CH_2O), 2.46 (t, *J* = 7.1 Hz, 2H, $C{\equiv}CCH_2CH_2$), 1.68 – 1.55 (m, 2H, CH_2CH_2), 1.40 – 1.21 (m, 27H, CH_3CH_2O, $(CH_2)_{12}$), 0.86 (t, *J* = 6.7 Hz, 3H, CH_3). ^{13}C NMR (75 MHz,

$CDCl_3$) δ 169.6, 159.3, 102.4, 79.7, 63.0, 31.9, 29.6, 29.5, 29.3, 28.9, 28.8, 27.3, 22.6, 19.4, 14.0, 13.9, 7.7. HRMS (ESI) $[M+Na]^+$ calcd for $C_{21}H_{36}NaO_3$, 359.2557; found: 359.2555.

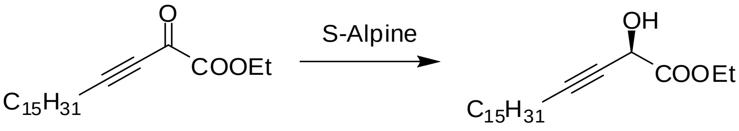

31 **32**

Ethyl(*R*)-2-hydroxynonadec-3-ynoate 32

(*S*)-Alpine borane (8.00 mL, 4.00 mmol, 0.5 *M* in THF) was added to a solution of compound **31** (0.28 g, 2.00 mmol) under the protection of nitrogen at 0 °C,[7] and the solvent was evacuated at room temperature under vacuum. After the reaction was completed by TLC detection, ethyl ether (10 mL) and silica gel (1.00 g) was added to quench the reaction, and the solution was stirred for 1 h. Then the solution was filtered and the filtrate was concentrated under vacuum.[7] The residue was purified using silica gel chromatography (10% ethyl acetate in hexanes) to give ethyl (*R*)-2-hydroxynonadec-3-ynoate **32** as a colorless transparent oil (0.51 g, 75% yield). $[\alpha]_D$ = -26.9 (*c* 0.54, $CHCl_3$). ^1H NMR (300 MHz, $CDCl_3$) δ 4.81 (d, *J* = 7.3 Hz, 1H, C≡C<u>H</u>OH), 4.31 (q, *J* = 7.2 Hz, 2H, $CH_3C\underline{H_2}O$), 3.00 (d, *J* = 7.4 Hz, 1H, CHO<u>H</u>), 2.21 (td, *J* = 7.1, 2.1 Hz, 2H, $C{\equiv}C\underline{H_2}CH_2$), 1.56 – 1.44 (m, 2H, $C\underline{H_2}CH_2$), 1.40 – 1.17 (m, 27H, $C\underline{H_3}CH_2O$, $(C\underline{H_2})_{\underline{12}}$), 0.88 (t, *J* = 6.7 Hz, 3H, $C\underline{H_3}$). ^{13}C NMR (75 MHz, $CDCl_3$) δ 170.8, 86.8, 75.6, 62.6, 61.7, 31.9, 29.7, 29.7, 29.6, 29.6, 29.5, 29.3, 29.1, 28.8, 28.3, 26.2, 22.7, 22.0, 18.7, 14.1, 14.0. HRMS (ESI) $[M+Na]^+$ calcd for $C_{21}H_{38}NaO_3$, 361.2713; found: 361.2710. HPLC: 97% *ee*. (Determined by chiral HPLC with column AD-H, chromatographic condition: isopropyl alcohol/hexane =2:98, v/v, flow rate: 1.0 mL/min, detection wavelength 220 nm, rention time: tmajor = 8.50 min, tminor = 9.60 min.)

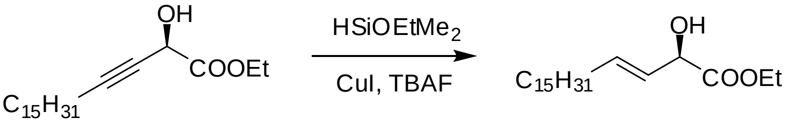

32 **33**

Ethyl(*R*)-2-acetoxynonadec-3-ynoate 33

Diethoxydimethylsilane (207 mg, 1.98 mmol) and $[CP{\cdot}Ru(MeCN)_3]PF_6$ (15.4 mg, 0.03 mmol in DCM 5 mL) was added to a solution of compound **32** (341 mg, 1.00 mmol) and anhydrous DCM at 0 °C under the protection of nitrogen. The solution was stirred for 30 min at 0 °C, and warmed to room temperature and stirred for 1 h. After the reaction was completed by TLC detection, the solution

was filtered and the filtrate was concentrated under vacuum to obtain a red brown oil.

Tetrabutylammonium fluoride solution (3 mL, 1 *M* in THF, 3mmol) was added to a mixed solution of the above obtained red brown oil, copper(I) iodide (570 mg, 3.00 mmol) and anhydrous THF (20 mL) at -30 °C, and the solution was kept under stirring for 16 h. After the reaction was completed by TLC detection, saturated aqueous ammonium chloride solution (50 mL) was added to quench the reaction, and the product was extracted with ethyl ether (3 × 50 mL).The combined organic phase was washed by saturated sodium chloride solution (3 × 100 mL), dried over sodium sulfate and concentrated under vacuum.[8] The residue was purified using silica gel chromatography (8% ethyl acetate in hexanes) to give ethyl(*R*)-2-acetoxynonadec-3-ynoate **33** as a colorless transparent oil (238 mg, 70% yield for two steps). $[\alpha]_D$ = -46.7 (*c* 0.55, $CHCl_3$). ^{1}H NMR (300 MHz, $CDCl_3$) δ 5.95 – 5.82 (m, 1H, HOCHCH=CH), 5.55 – 5.45 (m, 1H, HOCHCH=CH), 4.58 (t, *J* = 5.8 Hz, 1H, HOCHCH=CH), 4.33 – 4.14 (m, 2H, CH_3CH_2O), 2.88 (d, *J* = 6.0 Hz, 1H, CHOH), 2.06 (dd, *J* = 14.2, 6.9 Hz, 2H,CH=$CHCH_2CH_2$), 1.41 – 1.22 (m, 29H,CH_3CH_2O, $(CH_2)_{13}$), 0.88 (t, *J* = 6.6 Hz, 3H, CH_3). ^{13}C NMR (75 MHz, $CDCl_3$) δ 173.5, 134.1, 126.1, 71.2, 61.4, 31.7, 29.5, 29.4, 29.3, 29.1, 28.9, 28.7, 27.0, 25.4, 24.5, 22.5, 13.9, 13.8. HRMS (ESI) $[M+Na]^+$ calcd for $C_{21}H_{40}NaO_3$, 363.2870; found: 363.2869.

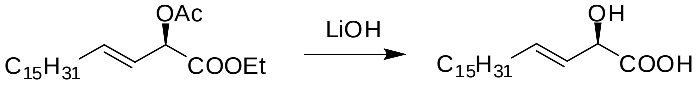

33 **34**

(2*R*,3*E*)-2-Hydroxynonadec-3-enoic acid 34

Lithium hydroxide (1.72 g, 103 mmol) and compound **33**(1.38 g, 4.05 mmol, in THF 32 mL)in order was add to a solution of methanol/water (10 mL/20 mL) at 0 °C. Then the solution was warmed to room temperature, and stirred for 4 h. After the reaction was completed by TLC detection, hydrochloric acid (1 *M*) was added to adjust pH 1-2, and the product was extracted with ethyl ether (3 × 50 mL).The combined organic phase was washed by saturated sodium chloride solution (3 × 100 mL), dried over sodium sulfate and concentrated under vacuum to get colorless transparent oil (1.10 g), which was directly used in the next step.

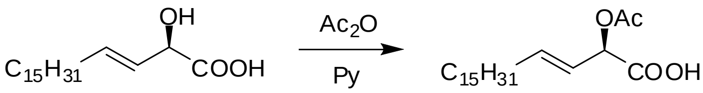

34 **35**

(2*R*,3*E*)-2-Acetoxynonadec-3-enoic acid 35

Acetic anhydride (1.00 mL) was added to a mixed solution of compound **34** (1.10 g, 3.00 mmol), DCM (30 mL), pyridine (7.5 mL) at room temperature and stirred for overnight. After the reaction was completed by TLC detection, hydrochloric acid (1 *M*) was added to adjust pH 1-2, and the product was extracted with DCM (3 × 50 mL).The combined organic phase was washed by water (3 × 100 mL), dried over sodium sulfate and concentrated under vacuum to get colorless transparent oil (1.05 g), which was directly used in the next step.

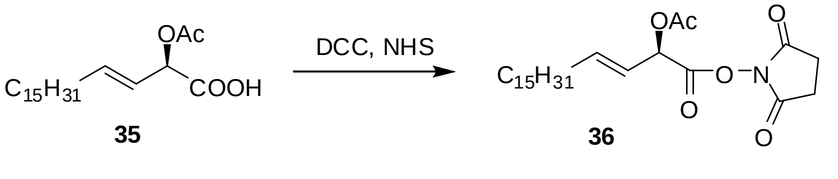

2,5-Dioxopyrrolidin-1-yl (2*R*,3*E*)-2-acetoxynonadec-3-enoate 36

Compound **35** (0.36 g, 1.00 mmol) was added to a solution of DCM (30 mL), N-hydroxysuccinimide (0.13 g, 1.20 mmol), dicyclohexylcarbodiimide (0.26 g, 1.20 mmol) at room temperature and stirred for overnight. After the reaction was completed by TLC detection, the solution was filtered, and the filtrate was concentrated under vacuum to obtain colorless transparent oil (1.05 g), which was directly used in the next step.

Preparation of Chrysogeside B and Analogues

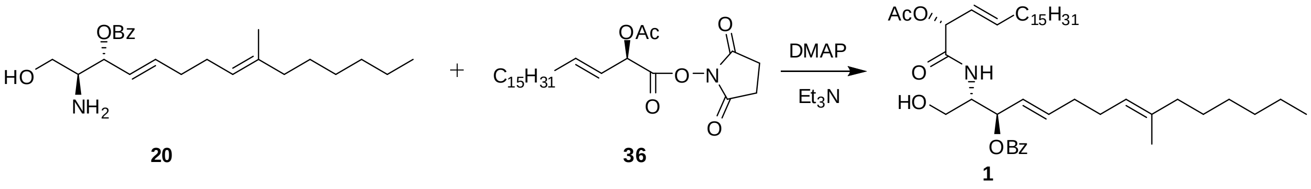

(2*R*,3*E*)-2-acetoxy-N-[(2*S*,3*R*,4*E*,8*E*)-1-hydroxy-3-benzoyloxy-9-methylpentadec-4,8-dien-2-yl]nonadec-3-enamide 1

Triethylamine (0.15 mL, 1.50 mmol) was added to a solution of the above obtained compound **20**, compound **36** (541 mg, 1.20 mmol), DCM (30 mL) and 4-dimethylamino pyridine (10 mg) at room temperature, and the reaction mixture was stirred for overnight. After the reaction was completed by TLC detection, the solution was concentrated under vacuum.[4] The residue was purified using silica gel chromatography (25% ethyl acetate in hexanes) to give compound **1** as a colorless amorphous solid (552 mg, 65% yield). $[\alpha]_D$ = +6.5 (*c* 0.70, $CHCl_3$). ^1H NMR (300 MHz, $CDCl_3$) δ 8.05 (d,

J = 7.9 Hz, 2H, Ar-H), 7.61 (t, *J* = 6.8 Hz, 1H, Ar-H), 7.48 (t, *J* = 7.6 Hz, 2H, Ar-H), 6.79 (d, *J* = 8.4 Hz, 1H, C=ONH), 6.03 – 5.00 (m, 7H, CH=CH, OAcCH, OBzCH), 4.33 – 4.13 (m, 1H, NHCH), 3.83 – 3.62 (m, 2H, CH_2O), 2.26 – 1.56 (m, 11H, OAc, $CH=CHCH_2$), 1.49 (s, 3H, $CH=CHCH_3$), 1.42 – 1.14 (m, 34H, CH_2), 0.87 (t, *J* = 6.6 Hz, 6H, CH_2CH_3). ^{13}C NMR (75 MHz, $CDCl_3$) δ 169.4, 169.1, 166.7, 138.2, 137.3, 137.1, 133.7, 131.0, 123.0, 129.8, 128.7, 126.2, 125.5, 124.9, 123.1, 74.8, 74.7, 61.7, 54.0, 39.8, 32.7, 32.5, 32.1, 31.8, 30.3, 29.9, 29.8, 29.6, 29.5, 29.4, 29.3, 29.1, 28.9, 28.1, 28.0, 27.4, 27.3, 26.9, 22.8, 21.1, 16.2, 16.0, 14.2. HRMS (ESI) calcd $[M+Na]^+$ for $C_{44}H_{71}NNaO_6$, 732.5174; found: 732.5178.

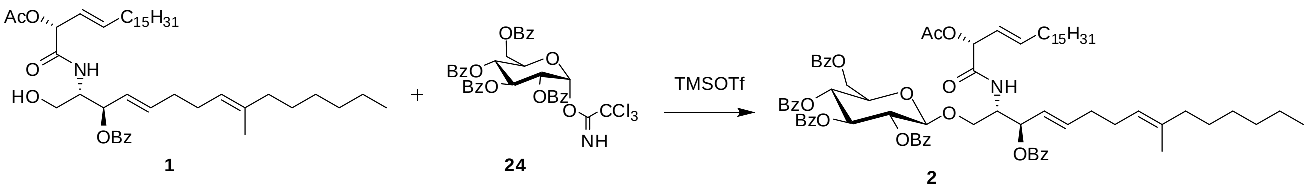

(2*R*,3*E*)-2-acetoxy-N-[(2*S*,3*R*,4*E*,8*E*)-1-(2,3,4,6-tetrabenzoyloxy-l-β-D-glucopyranosyloxy)-3-benzoyloxy-9-methylpentadec-4,8-dien-2-yl]nonadec-3-enamide 2

Mixed solvents (anhydrous ethyl ether /THF=2:1, 5 mL) was added to a solution of compound **1** (100 mg, 0.14 mmol), compound **24** (121 mg, 0.15 mmol), 4A molecular sieve (1 g), under the protection of nitrogen. The mixed solution was stirred at room temperature for 1 h, and was cooled to -30 °C, and then trimethylsilyl trifluoromethanesulfonate (0.65 uL) was dropped. After the reaction was completed by TLC detection, triethylamine (1 mL) was added to the solution. The mixture was filtered and the filtrate was concentrated under vacuum. The residue was purified using silica gel chromatography (20% ethyl acetate in hexanes) to give compound **2** as a colorless amorphous solid (108 mg, 60% yield). $[\alpha]_D$ = +15.2 (*c* 1.14, $CHCl_3$). ^1H NMR (300 MHz, $CDCl_3$) δ8.10 (d, *J* = 7.2 Hz, 2H, Ar-H), 8.00 – 7.81 (m, 6H, Ar-H), 7.72 – 7.30 (m, 17H, Ar-H), 6.44 (d, *J* = 9.2 Hz, 1H, C=ONH), 6.02 – 5.84 (m, 2H, BzOCHCH=CH, AcOCHCH=CH), 5.84 – 5.44 (m, 6H, $CH_2CH=CH$, H-3, H-4, H-2), 6.02 – 5.21 (m, 2H, OAcCH, OBzCH), 4.79 (d, *J* = 8.0 Hz, 1H, H-1), 4.56 – 4.25 (m, 3H, NHCH, H-6), 4.12 – 4.00 (m, 1H, H-5), 3.69 – 3.56 (m, 1H, CH_aH_bO), 3.41 – 3.26 (m, 1H, CH_aH_bO), 2.10 (s, 3H, OAc), 2.04 – 1.65 (m, 8H, $CH=CHCH_2$), 1.45 (s, 3H, $CH=CHCH_3$), 1.39 – 1.15 (m, 34H, CH_2), 0.88 (t, *J* = 6.1 Hz, 6H, CH_2CH_3). ^{13}C NMR (75 MHz, $CDCl_3$) δ 169.4, 168.6, 166.1, 165.9, 165.3, 138.0, 136.1, 133.7, 133.6, 133.5, 133.4, 133.2, 133.1, 130.1, 123.0, 129.9, 129.8, 129.6, 129.5, 129.3, 129.2, 129.1, 128.9, 128.8, 128.7, 128.6, 128.5, 125.6, 122.9, 101.0, 72.4, 72.3, 69.7, 63.1, 50.9, 38.0, 32.4,

32.1, 31.7, 29.8, 29.6, 29.5, 29.4, 28.8, 27.1, 23.5, 22.8, 22.7, 20.8, 14.2, 14.1. HRMS (ESI) $[M+Na]^+$ calcd for $C_{78}H_{97}NNaO_{15}$, 1310.6749; found: 1310.6742.

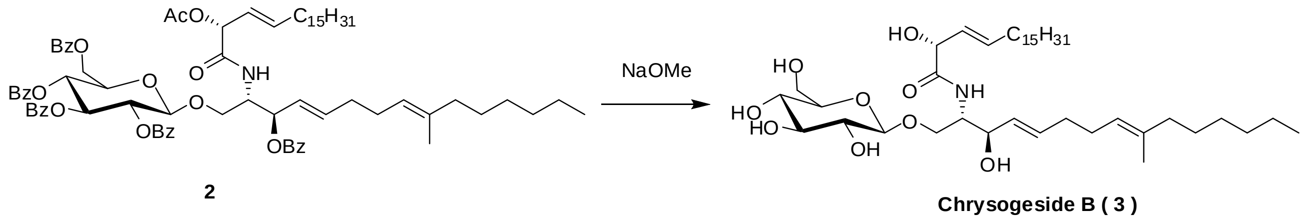

Chrysogeside B (3)

Sodium methoxide solution (0.05 mL 0.5 *M* in methanol, 0.025 mmol) was added to the solution of compound **2** (90 mg, 0.09 mmol) and anhydrous methanol (5 mL)at 0 °C, and the solution was stirred at room temperature for 2 h. After the reaction was completed by TLC detection, ambrest 15 was added to adjust pH 6-7. After the mixture was filtered and filtrate was concentrated under vacuum. The residue was purified using silica gel chromatography (10% methanol /acetate in chloroform) to give **Chrysogeside B (3)** as a colorless amorphous solid (43 mg, 85% yield). The resulting product was confirmed by ^1H NMR ^{13}C NMR and HRMS. $[\alpha]_D$ = -8.1 (c 0.5, CH_3OH). (lit.[9] $[\alpha]_D$ = -8.0 (*c* 0.5, CH_3OH)). ^1H NMR (300 MHz, CD_3OD) δ 5.96 – 5.80 (m, 1H, HOCHC<u>H</u>=CH), 5.80 – 5.67 (m, 1H, HOCHC<u>H</u>=CH), 5.66 – 5.26 (m, 3H, CH_2C<u>H</u>=CH), 4.63 – 4.39 (m, 1H, O=CC<u>H</u>OH), 4.27 (d, *J* = 7.6 Hz, 1H, <u>H</u>-<u>1</u>), 4.23 – 4.05 (m, 3H, COC<u>H₂</u>, NHCHC<u>H</u>OH), 4.04 – 3.92 (m, 1H, NHC<u>H</u>CHOH), 3.92 – 3.81 (m, 1H, <u>H</u>-<u>3</u>), 3.80 – 3.60 (m, 2H, <u>H</u>-<u>4</u>, <u>H</u>-<u>2</u>), 3.30 – 3.26 (m, 2H, <u>H</u>-<u>6</u>), 3.25 – 3.15 (m, 1H, <u>H</u>-<u>5</u>), 2.36 – 1.89 (m, 6H, CH=CHC<u>H₂</u>), 1.73 – 1.48 (m, 2H, C<u>H₂</u>$(CH_2)_4CH_3$), 1.43 (s, 3H, CH=CHC<u>H₃</u>), 1.29 (s, 34H, C<u>H₂</u>), 0.90 (t, *J* = 6.6 Hz, 6H, CH_2C<u>H₃</u>). ^{13}C NMR (75 MHz, CD_3OD) δ 175.5, 135.6, 135.1, 134.8, 132.4, 130.9, 129.9, 129.0, 104.7, 78.0, 75.0, 74.1, 73.3, 72.9, 71.6, 69.6, 62.7, 55.0, 42.8, 34.0, 33.4, 33.1, 31.2, 30.8, 30.7, 30.4, 30.2, 26.9, 25.0, 24.6, 23.7, 19.5, 14.4. HRMS (ESI) $[M+Na]^+$ calcd for $C_{41}H_{75}NNaO_9$, 748.5334; found: 748.5338.

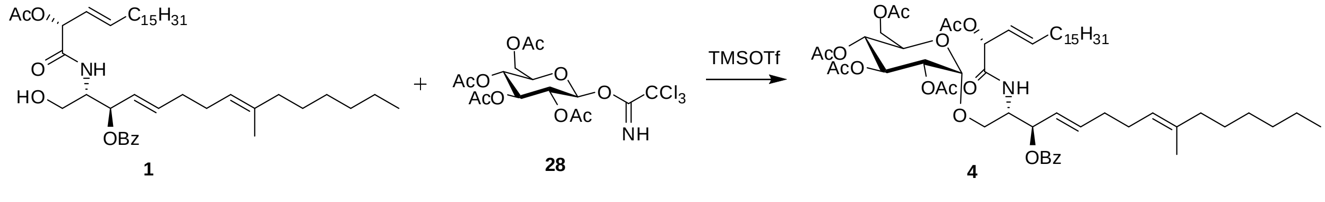

(2*R*,3*E*)-2-Acetoxy-N-[(2*S*,3*R*,4*E*,8*E*)-1-(2,3,4,6-tetraacetyloxy-l-α-D-glucopyranosyloxy)-3-benzoylo xy-9-methylpentadec-4,8-dien-2-yl]nonadec-3-enamide 4

Mixed solvents (anhydrous ethyl ether /THF=2:1, 5 mL) was added to a solution of compound **1**

(100 mg, 0.14 mmol), compound **28** (78 mg, 0.15 mmol), 4A molecular sieve (1 g) under the protection of nitrogen. The mixed solution was stirred at room temperature for 1 h, and was cooled to -30 °C, and then trimethylsilyl trifluoromethanesulfonate (0.65 uL) was dropped. After the reaction was completed by TLC detection, triethylamine (1 mL) was added to the solution in 10 min under stirring. The mixture was filtered and the filtrate was concentrated under vacuum. The residue was purified using silica gel chromatography (20% ethyl acetate in hexanes) to give compound **4** as a colorless amorphous solid (92 mg, 50% yield). $[\alpha]_D$ = +19.3 (*c* 0.76, $CHCl_3$). ^1H NMR (300 MHz, $CDCl_3$) δ 8.02 (d, *J* = 7.4 Hz, 2H, Ar-H), 7.57 (t, *J* = 7.2 Hz, 1H, Ar-H), 7.44 (t, *J* = 7.4 Hz, 2H, Ar-H), 6.47 (d, *J* = 9.2 Hz, 1H, C=ONH), 6.03 – 5.73 (m, 2H, BzOCHCH=CH, AcOCHCH=CH), 5.73 – 5.41 (m, 5H, CH_2CH=CH, H-3, H-4), 5.41 – 5.02 (m, 2H, OAcCH, OBzCH), 4.99 (d, *J* = 3.6 Hz, 1H, H-1), 4.50 – 4.36 (m, 1H, H-2), 3.86 – 3.72 (m, 2H, H-5, H-6a), 4.19 – 4.01 (m, 2H, H-6b, NHCH), 3.86 – 3.72 (m, 1H CH_aH_bO), 3.66 – 3.45 (m, 1H, CH_aH_bO), 2.18 – 2.01 (m, 15H, OAc), 1.99 – 1.60 (m, 8H, CH=$CHCH_2$), 1.47 (s, 3H, CH=$CHCH_3$), 1.44 – 1.11 (m, 34H, CH_2), 0.87 (t, *J* = 5.6 Hz, 6H CH_2CH_3). ^{13}C NMR (75 MHz, $CDCl_3$) δ 169.6, 169.5, 169.2, 168.5, 168.4, 168.3, 168.0, 137.1, 135.0, 132.6, 132.2, 128.8, 128.7, 127.6, 127.4, 124.5, 121.9, 95.9, 73.7, 73.6, 73.4, 71.5, 70.9, 67.3, 60.8, 60.4, 52.8, 36.9, 32.9, 31.3, 30.9, 30.6, 28.7, 28.6, 28.5, 28.3, 27.7, 27.6, 24.6, 23.9, 22.3, 21.7, 21.5, 19.8, 19.5, 18.2, 13.1, 13.0. HRMS (ESI) $[M+Na]^+$ calcd for $C_{58}H_{89}NNaO_{15}$, 1062.6114; found: 1062.6107.

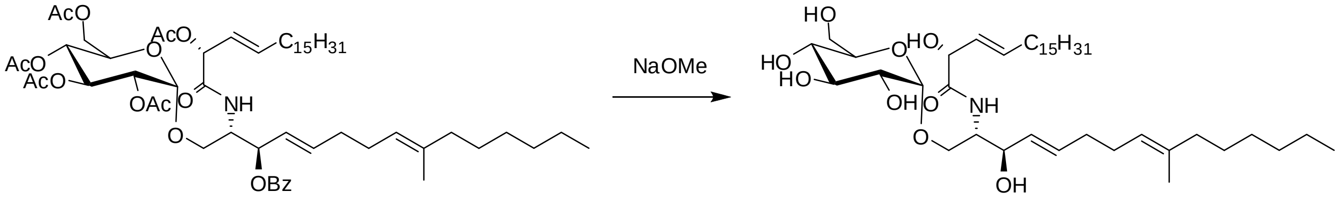

4 5

(2*R*,3*E*)-2-Hydroxy-N-[(2*S*,3*R*,4*E*,8*E*)-l-α-D-glucopyranosyloxy-3-hydroxy-9-methylpentadec-4,8-dien-2-yl]nonadec-3-enamide 5

Sodium methoxide solution (0.03 mL, 0.5 *M* in methanol, 0.015 mmol) was added to the solution of compound **4** (73 mg, 0.06 mmol) and anhydrous methanol (5 mL) at 0 °C, and the solution was stirred at room temperature for 2 h. When the reaction was completed by TLC detection, ambrest 15 was added to adjust pH 6-7. After the mixture was filtered and filtrate was concentrated under vacuum. The residue was purified using silica gel chromatography (10% methanol /acetate in chloroform) to give compound **5** as a colorless amorphous solid (41 mg, 80% yield). $[\alpha]_D$ = +5.4 (*c*

0.50, CH_3OH). ^{1}H NMR (300 MHz, CD_3OD) δ 5.96 – 5.81 (m, 1H, HOCHCH=CH), 5.80 – 5.67 (m, 1H, HOCHCH=CH), 5.66 – 5.22 (m, 3H, CH_2CH=CH), 4.72 – 4.44 (m, 2H, O=CCHOH, H-1), 4.32 – 4.06 (m, 3H, COCH_2, NHCHCHOH), 4.06 – 3.93 (m, 1H, NHCHCHOH), 3.94 – 3.80 (m, 1H, H-3), 3.80 – 3.48 (m, 3H, H-4, H-2, H-5), 3.30 – 3.14 (m, 2H, H-6), 2.36 – 1.47 (m, 8H, CH=CCH_2, CH=CHCH_2), 1.45 (s, 3H, CH=CHCH_3), 1.41 – 1.23 (m, 34H, CH_2), 0.92 (t, *J* = 6.7 Hz, 6H, CH_2CH_3). ^{13}C NMR (75 MHz, CD_3OD) δ 175.6, 135.4, 134.6, 134.3, 100.3, 129.0, 103.3, 78.1, 76.2, 75.8, 74.1, 73.3, 71.8, 62.0, 56.4, 42.3, 34.0, 33.4, 33.0, 31.2, 30.8, 30.7, 30.6, 30.4, 30.2, 26.9, 25.0, 24.6, 23.7, 20.9, 14.4. HRMS (ESI) $[M+Na]^+$ calcd for $C_{41}H_{75}NNaO_9$, 748.5334; found: 748.5335.

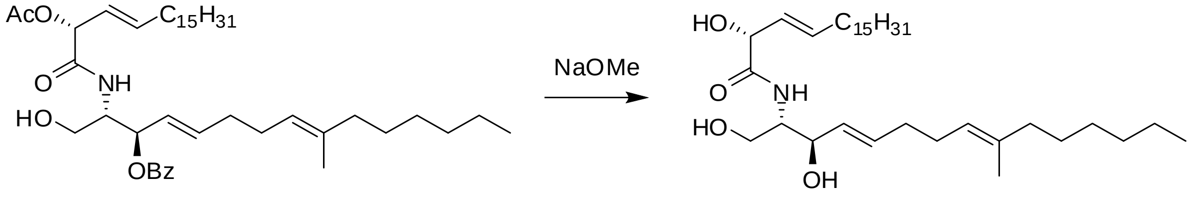

1 6

(2*R*,3*E*)-2-Hydroxy-N-[(2*S*,3*R*,4*E*,8*E*)-1-hydroxy-3-hydroxy-9-methylpentadec-4,8-dien-2-yl]nonadec-3-enamide 6

Sodium methoxide solution (0.06 mL, 0.5 *M* in methanol, 0.03 mmol) was added to the solution of compound **1** (100 mg, 0.14 mmol) and anhydrous methanol (5 mL) at 0 °C, and the solution was stirred at room temperature for 2 h. When the reaction was completed by TLC detection, ambrest 15 was added to adjust pH 6-7. After the mixture was filtered and filtrate was concentrated under vacuum. The residue was purified using silica gel chromatography (ethyl acetate in chloroform) to give compound **6** as a colorless amorphous solid (71 mg, 89% yield). $[\alpha]_D$ = -6.2 (*c* 0.65, CH_3OH). ^{1}H NMR (300 MHz, $CDCl_3$) δ 6.09 – 5.67 (m, 2H, HOCHCH=CH), 6.67 – 5.03 (m, 3H, CH_2CH=CH), 4.72 – 4.14 (m, 3H, CHNH, CHOH), 3.99 – 3.39 (m, 2H, CH_2O), 2.35 – 1.49 (m, 8H, CH=CHCH_2), 1.48 – 0.93 (m, 37H, CH=CHCH_3,CH_2), 0.88 (t, *J* = 5.8 Hz, 6H, CH_3). ^{13}C NMR (75 MHz, CD_3OD) δ 175.7, 135.7, 134.7, 130.9, 129.0, 128.5, 127.7, 74.0, 73.2, 62.0, 56.6, 42.2, 34.0, 33.4, 33.0, 31.2, 30.8, 30.7, 30.6, 30.4, 30.3, 30.2, 28.9, 26.9, 25.0, 23.7, 14.4. HRMS (ESI) $[M+H]^+$ calcd for $C_{35}H_{66}NO_4$, 564.4990; found: 564.4997.

^1H and ^{13}C NMR Spectra of All Products

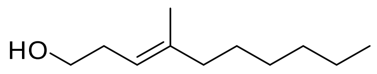

8

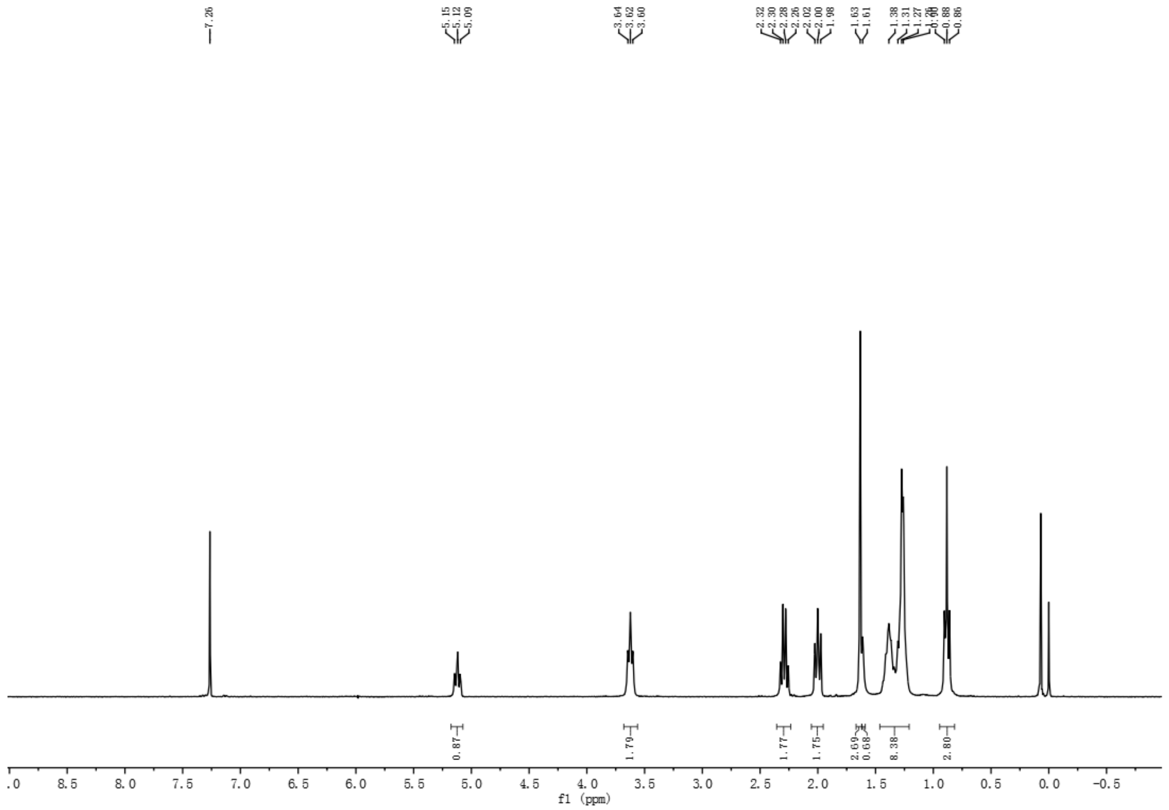

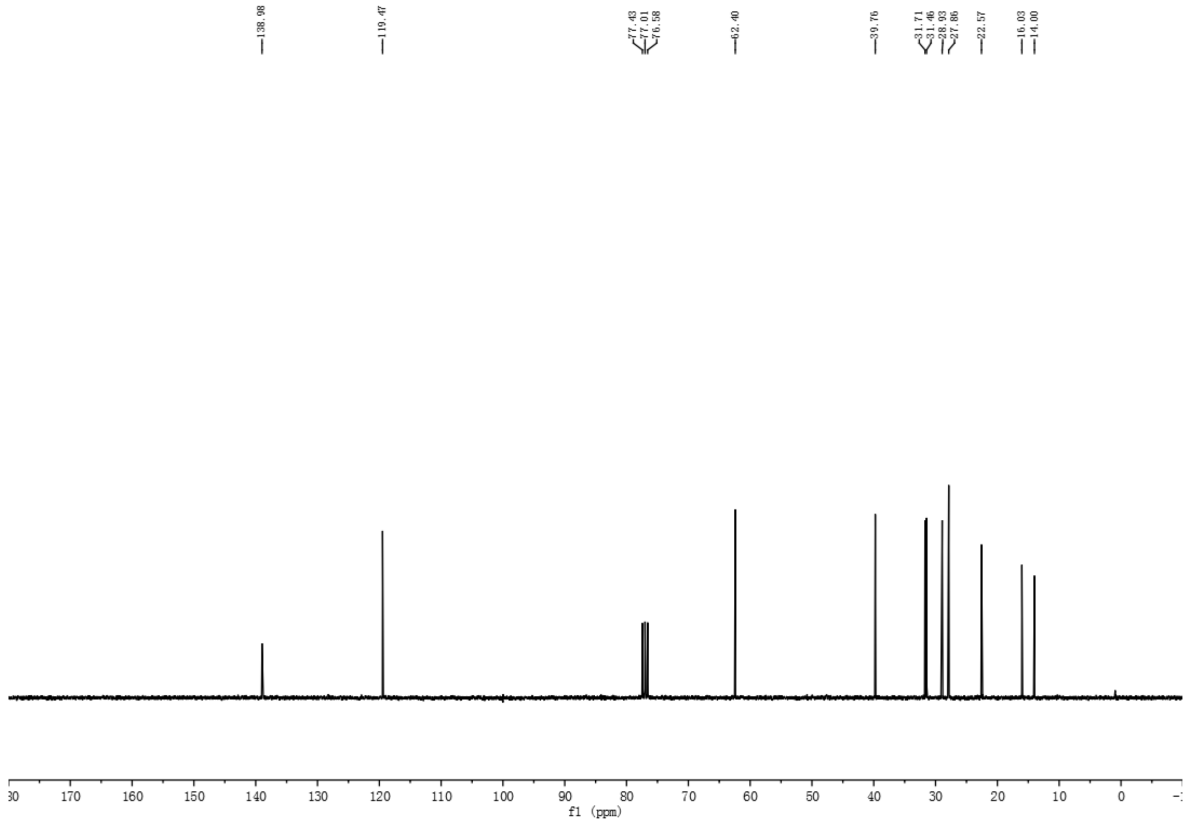

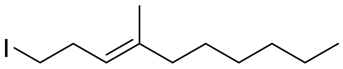

9

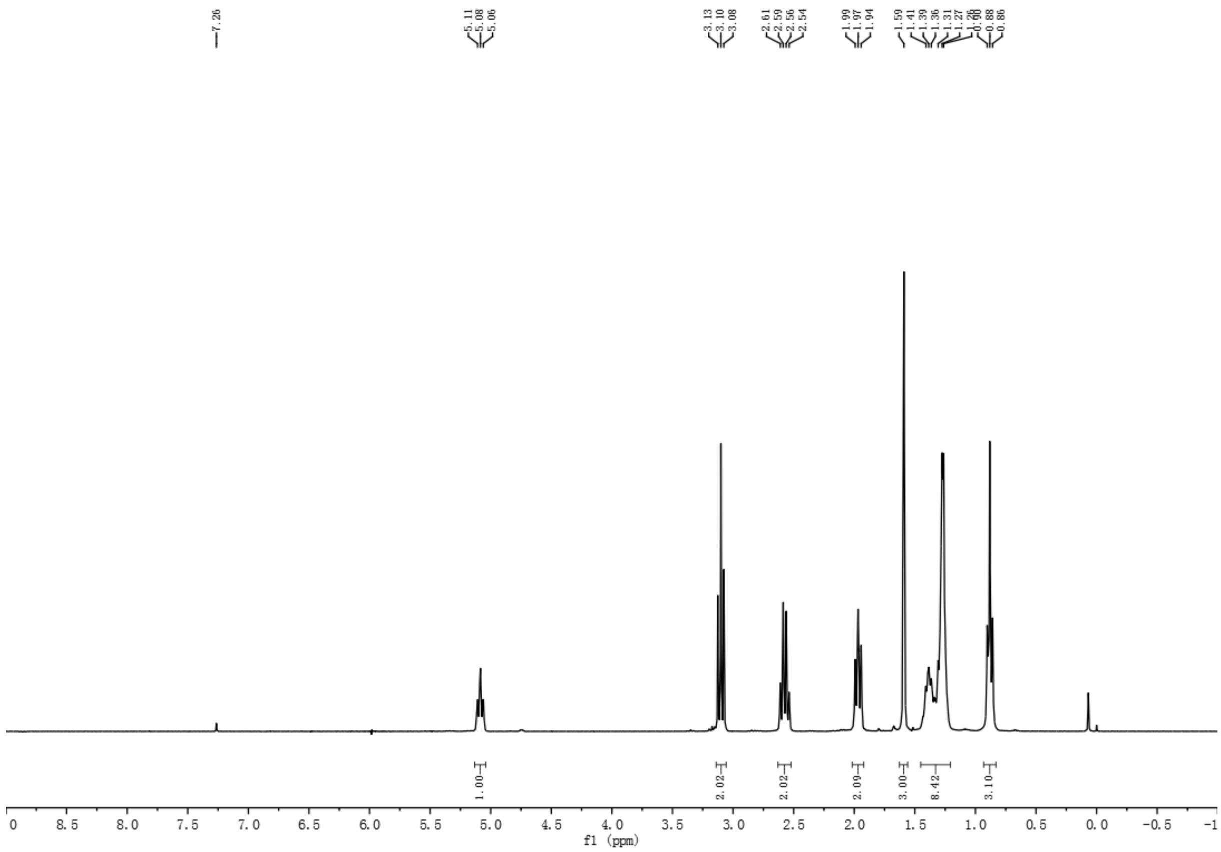
7.26
5.11
5.08
5.06
3.13
3.10
3.08
2.61
2.59
2.56
2.54
1.99
1.97
1.94
1.59
1.41
1.39
1.36
1.31
1.27
0.88
0.86
1.00
2.02
2.02
2.09
3.00
8.42
3.10
f1 (ppm)

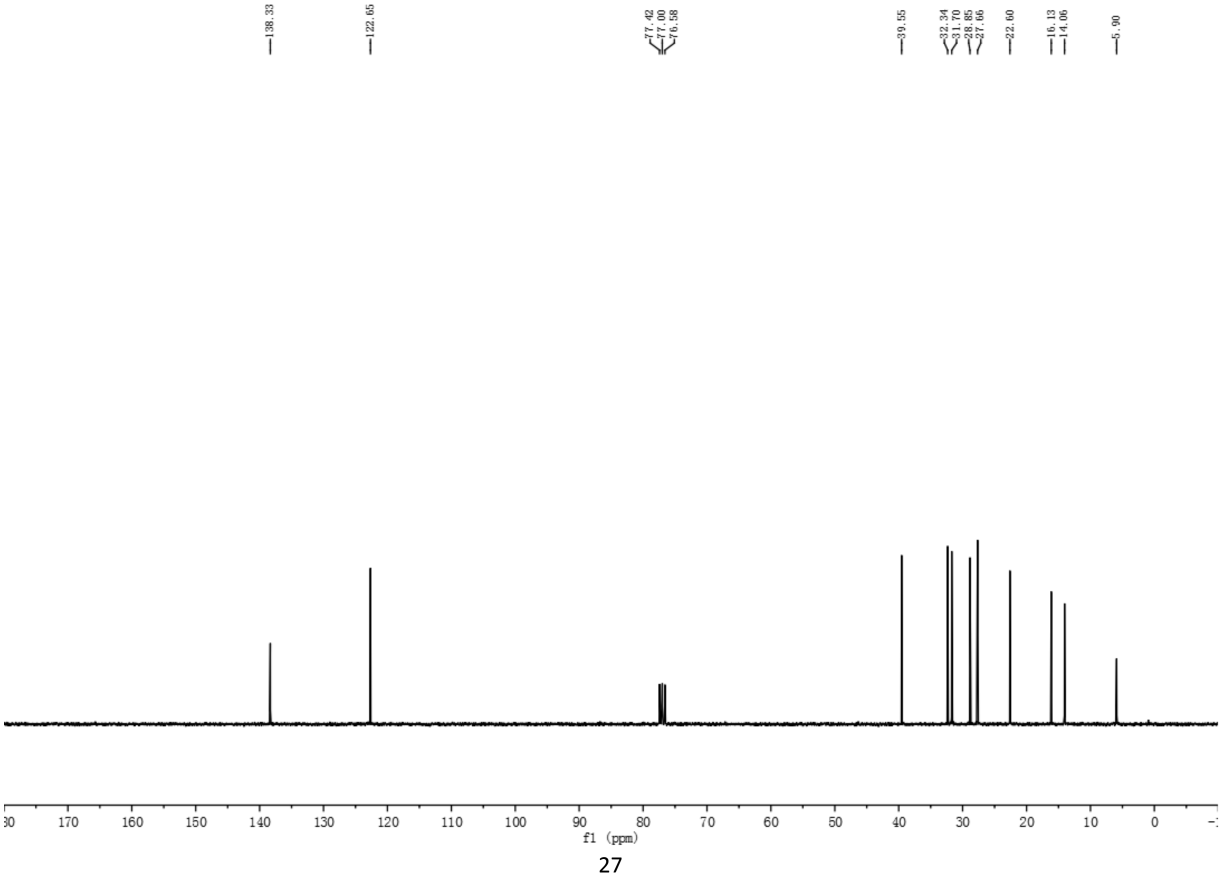
138.33
122.65
77.42
77.00
76.58
39.55
32.34
31.70
28.85
27.66
22.60
16.13
14.06
5.90
f1 (ppm)

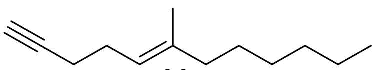

11

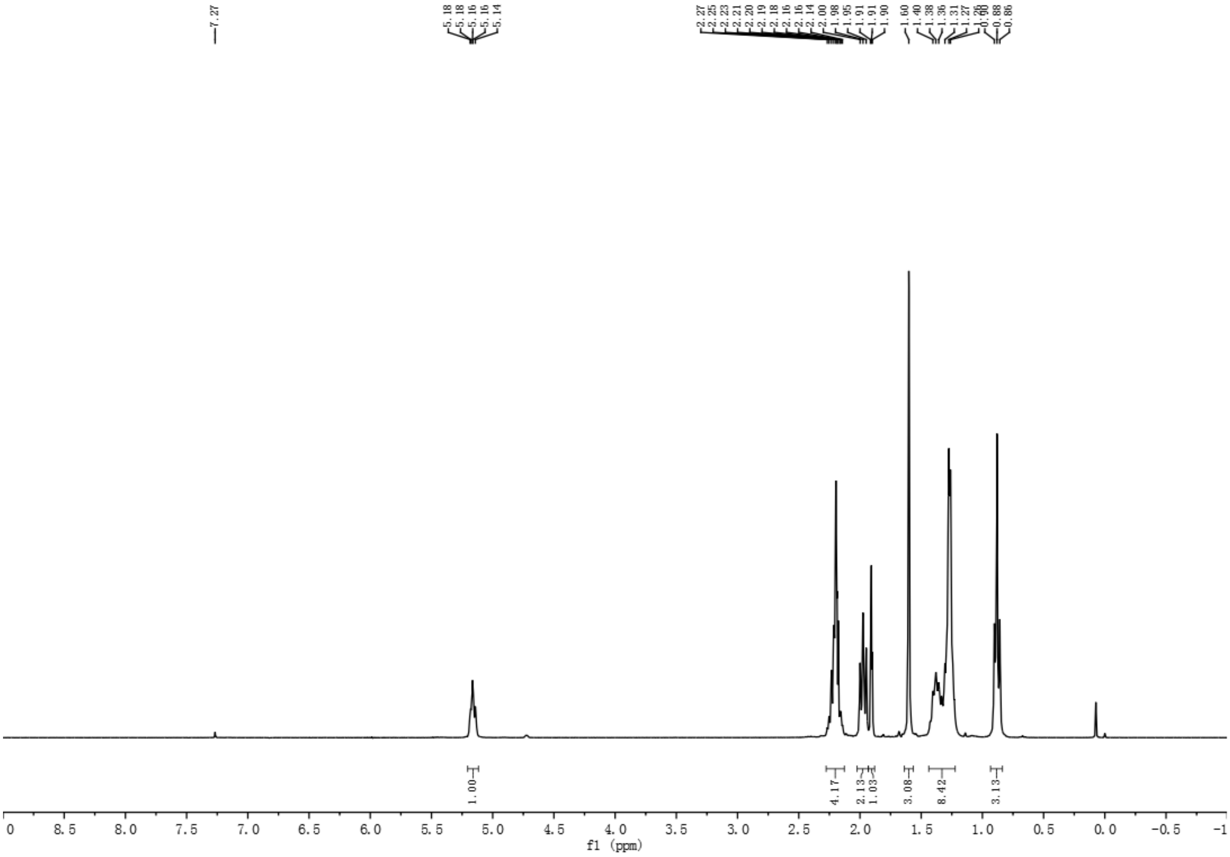
7.27
5.18
5.18
5.16
5.16
5.14
2.27
2.25
2.23
2.21
2.20
2.19
2.18
2.16
2.16
2.14
2.00
1.98
1.95
1.91
1.91
1.90
1.60
1.40
1.38
1.36
1.31
1.27
0.88
0.86
1.00
4.17
2.13
1.03
3.08
8.42
3.13
f1 (ppm)

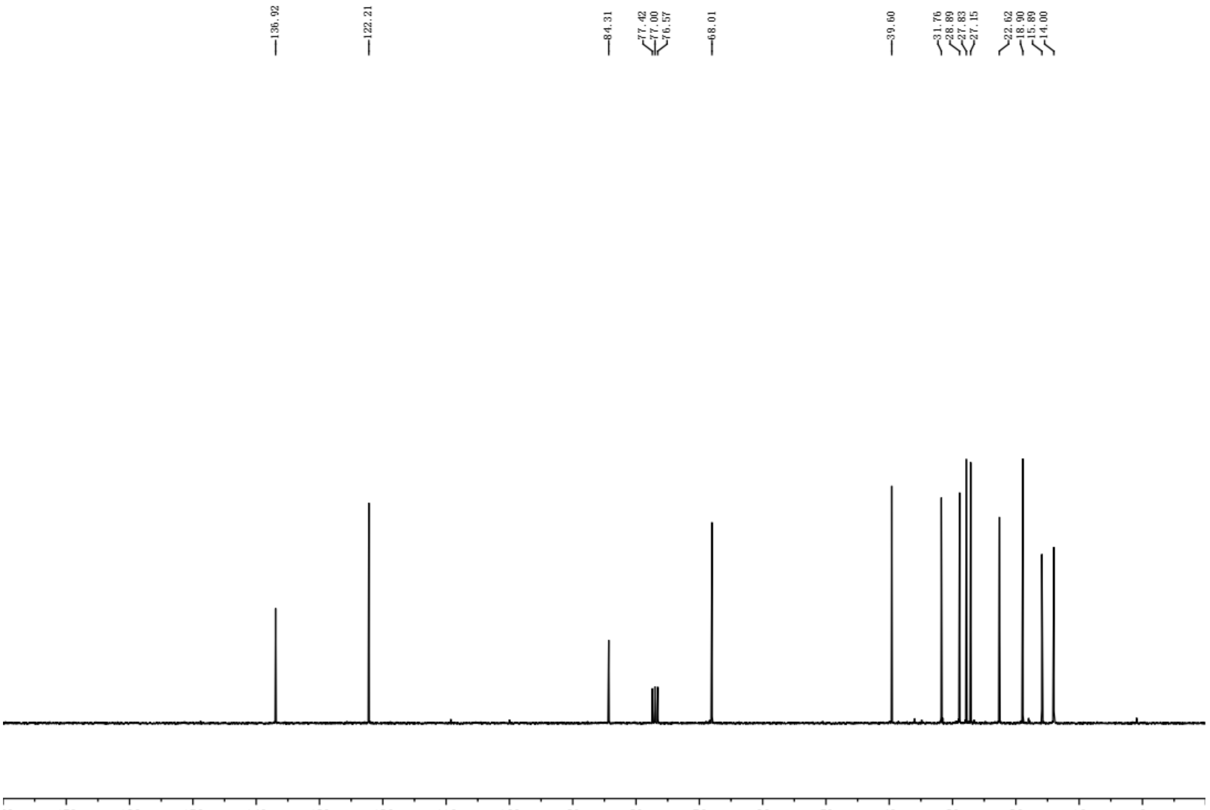
136.92
122.21
84.31
77.42
77.00
76.57
68.01
39.60
31.76
28.89
27.83
27.15
22.62
18.90
15.89
14.00
f1 (ppm)

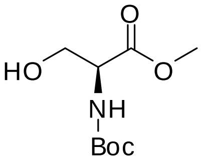
O
HO
O
NH
Boc

12

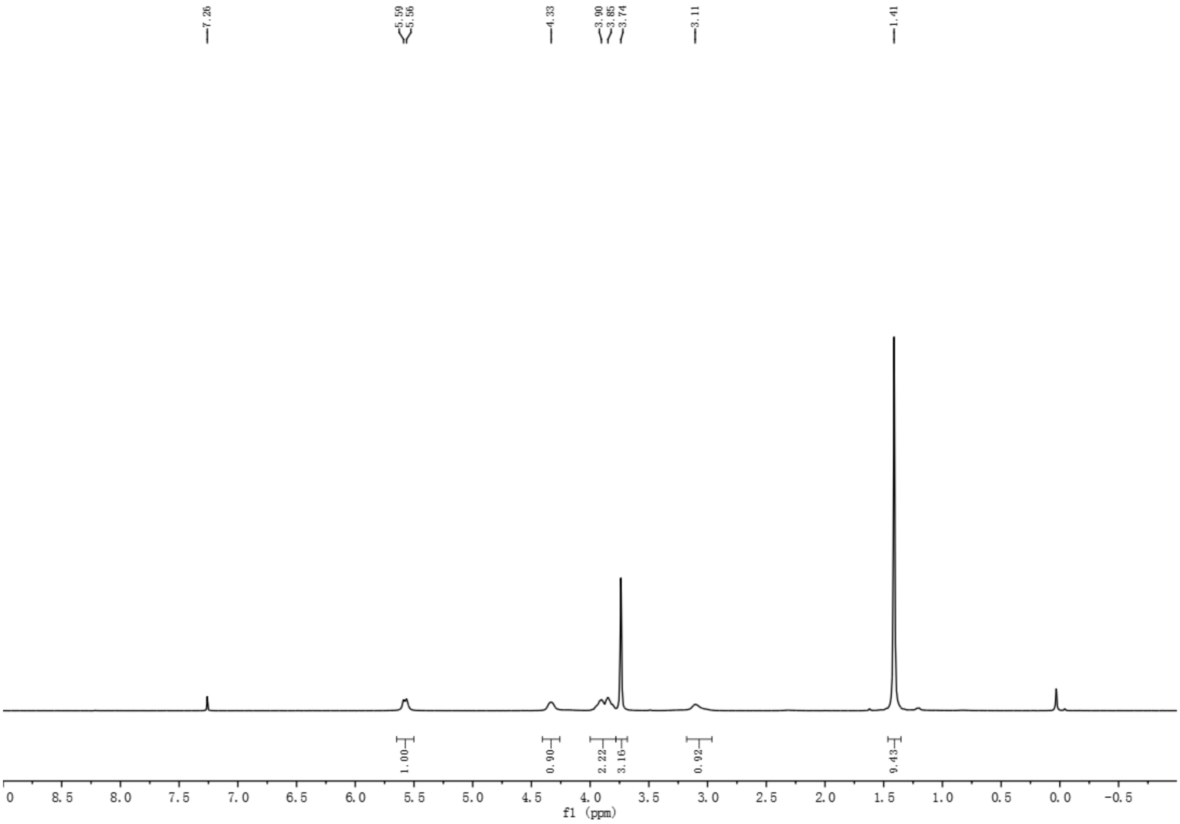
7.26
5.59
5.56
4.33
3.90
3.85
3.74
3.11
1.41
1.00
0.90
2.22
3.16
0.92
9.43
f1 (ppm)

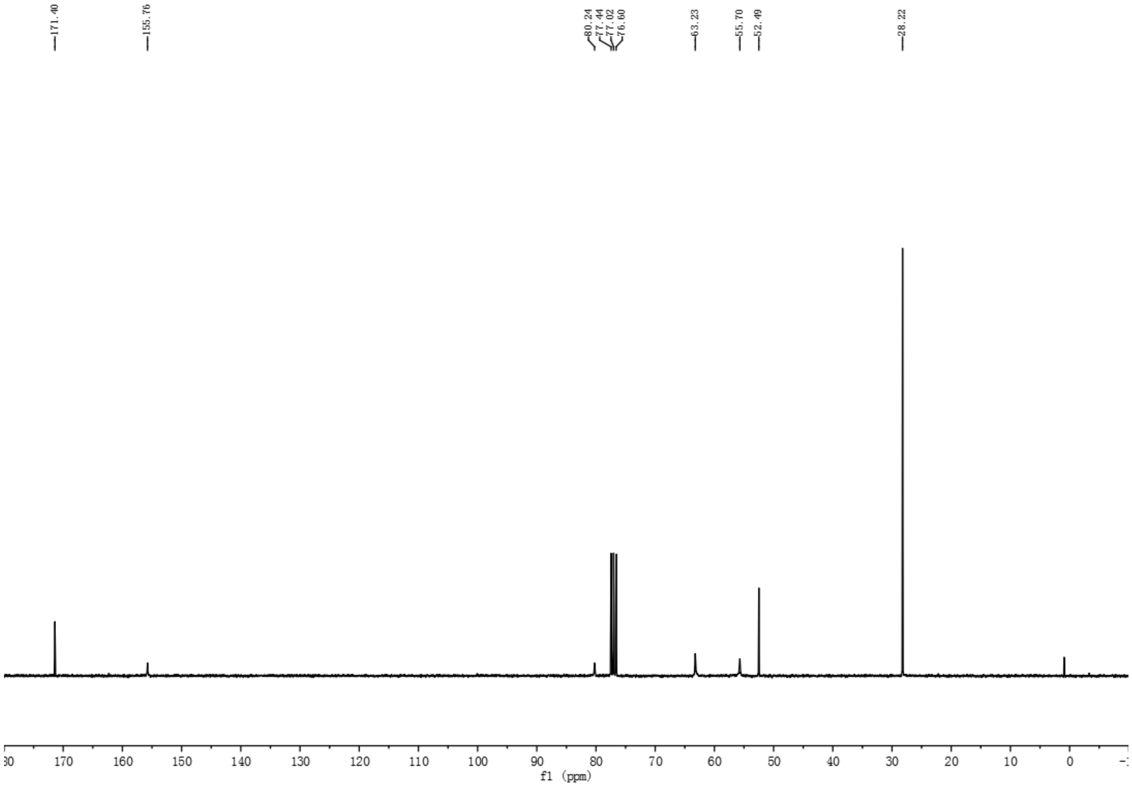
171.40
155.76
80.24
77.44
77.02
76.60
63.23
55.70
52.49
28.22
f1 (ppm)

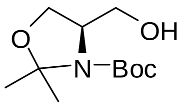
O
OH
N
Boc

14

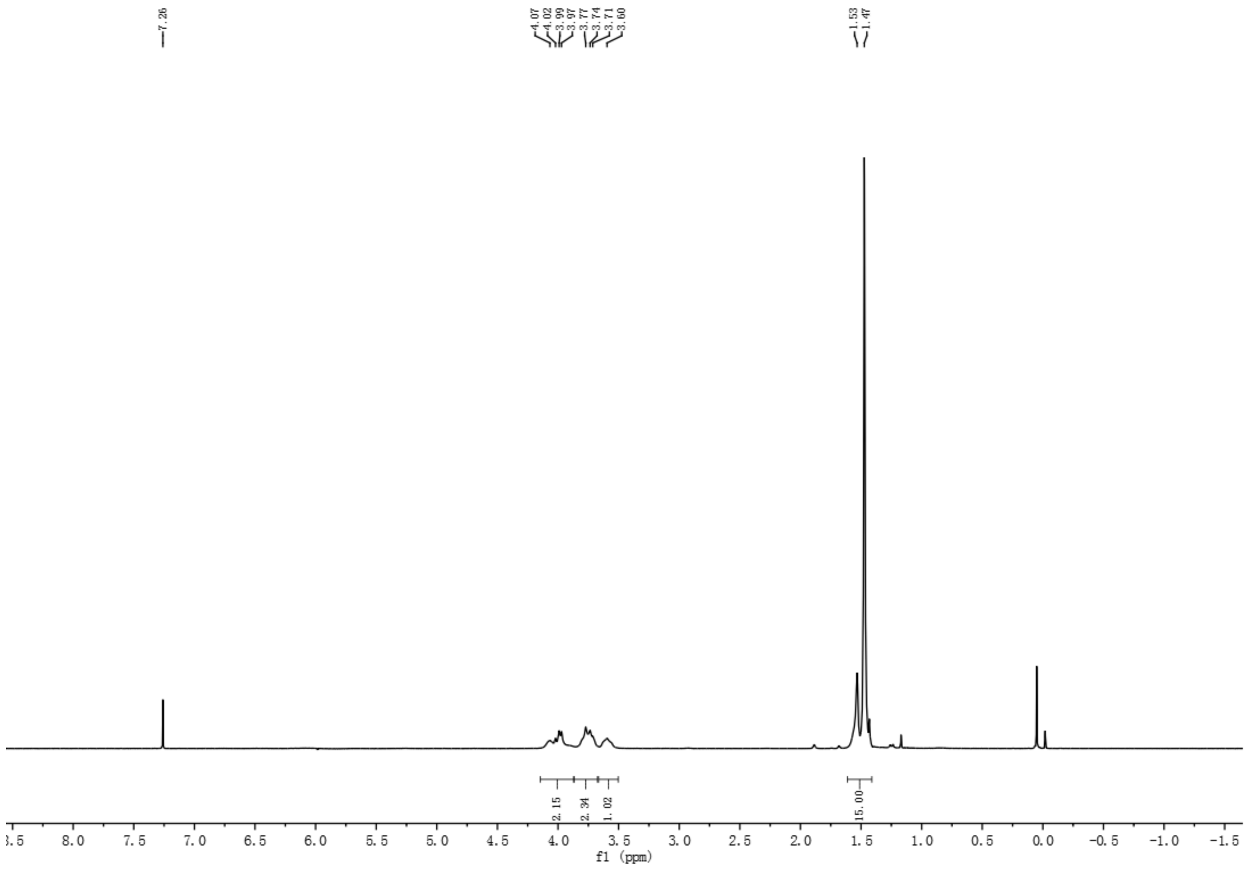
7.26
4.07
4.02
3.99
3.97
3.77
3.74
3.71
3.60
1.53
1.47
2.15
2.34
1.02
15.00
f1 (ppm)

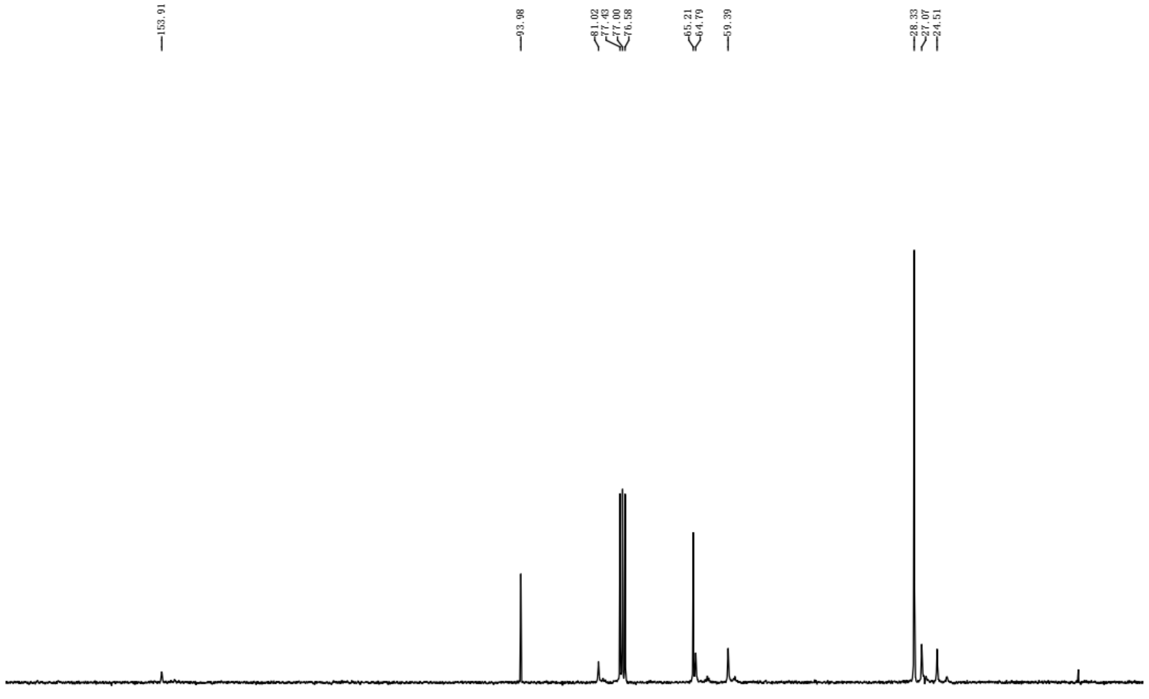
153.91
93.98
81.02
77.43
77.00
76.58
65.21
64.79
59.39
28.33
27.07
24.51
f1 (ppm)

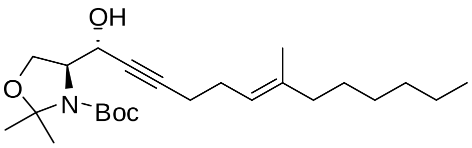
OH
O
N
Boc

16

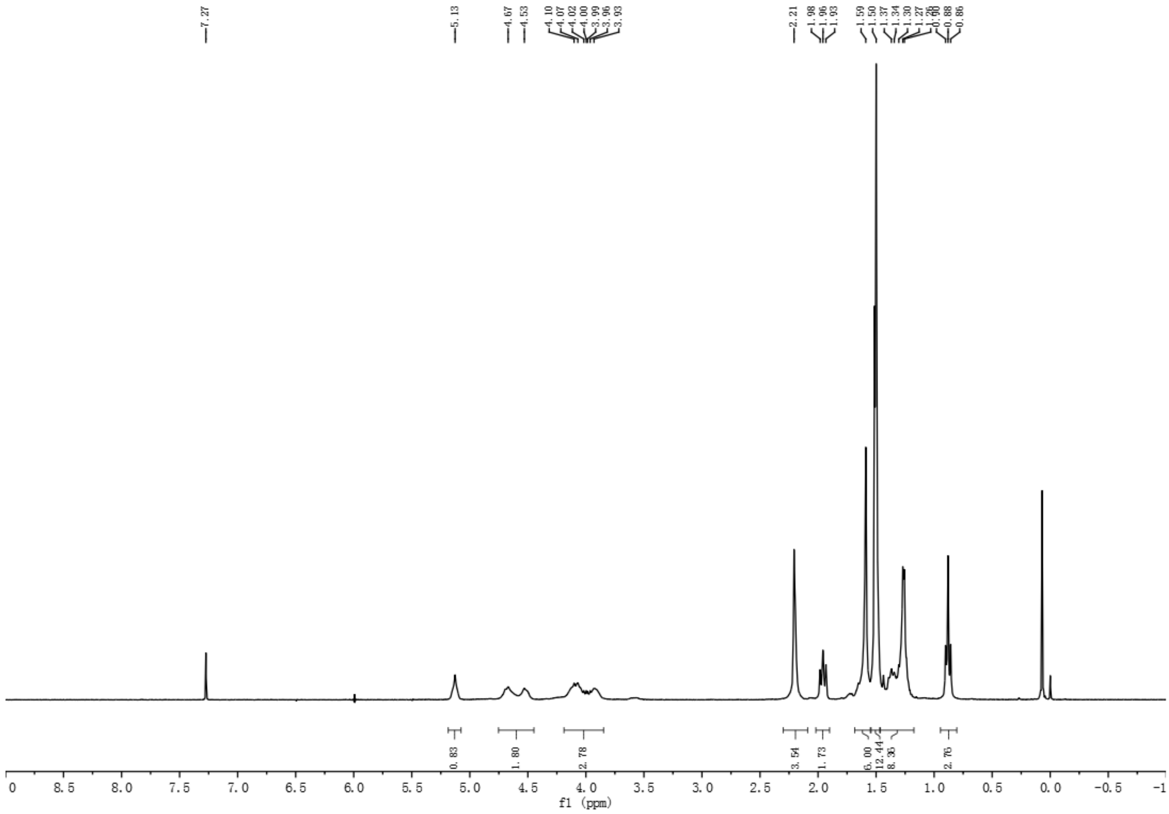
7.27
5.13
4.67
4.53
4.10
4.07
4.02
4.00
3.99
3.96
3.93
2.21
1.98
1.96
1.93
1.59
1.50
1.37
1.34
1.30
1.27
0.88
0.86
0.83
1.80
2.78
3.54
1.73
6.00
12.44
8.35
2.76
f1 (ppm)

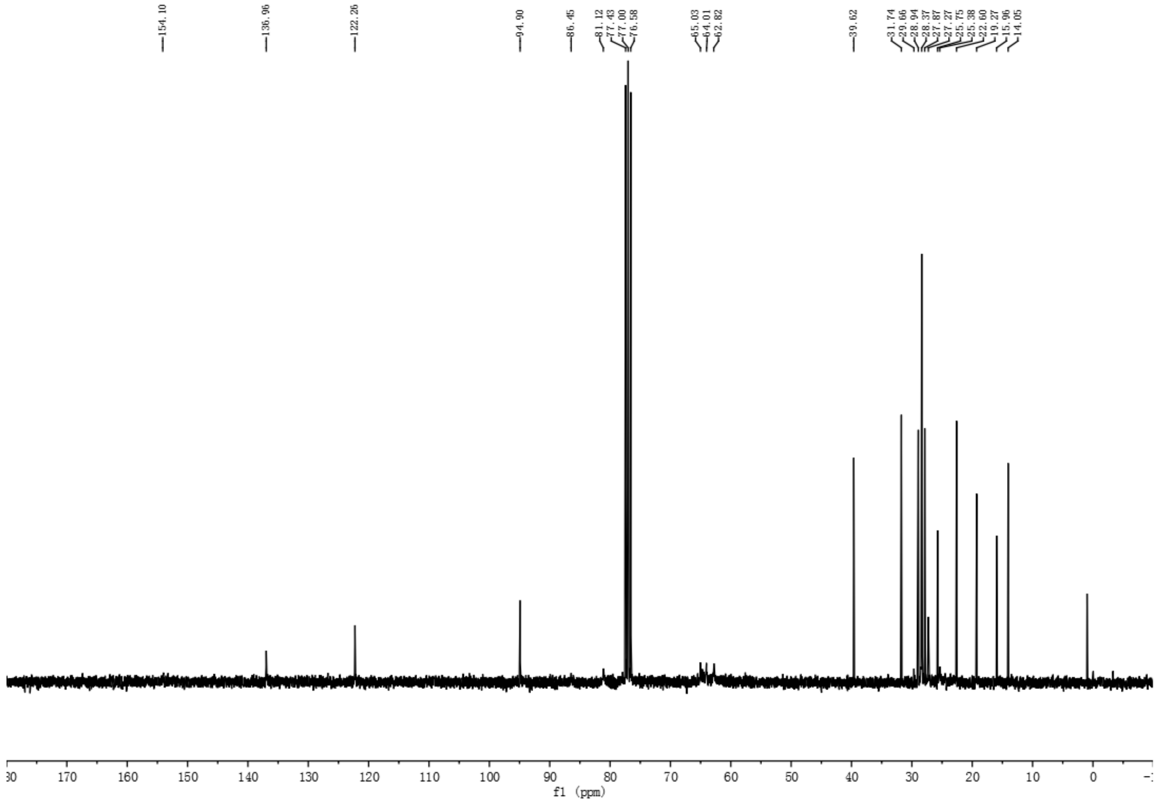
154.10
136.96
122.26
94.90
86.45
81.12
77.43
77.00
76.58
65.03
64.01
62.82
39.62
31.74
29.66
28.94
28.37
27.87
27.27
25.75
25.38
22.60
19.27
15.96
14.05
f1 (ppm)

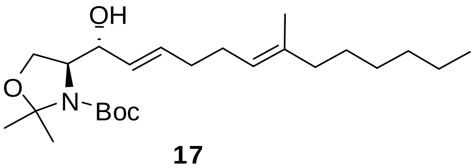
OH
O
N
Boc

17

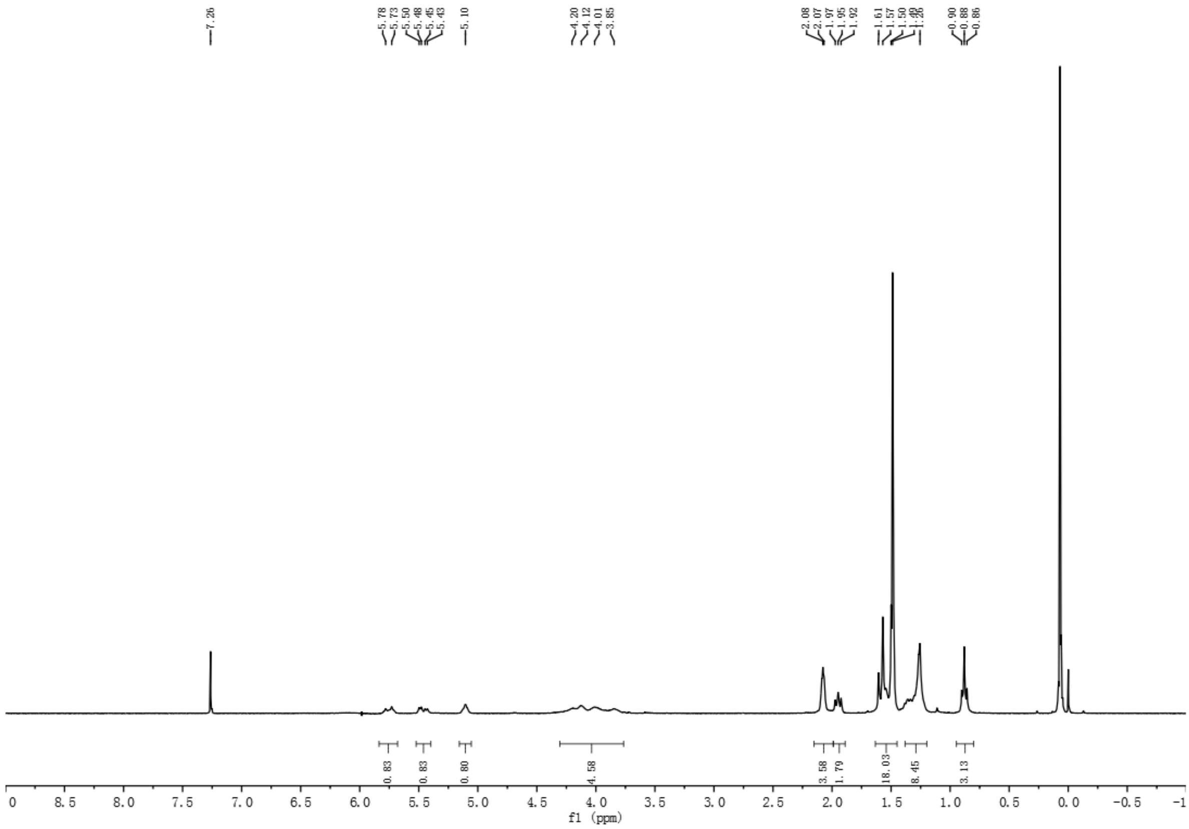
7.26
5.78
5.73
5.50
5.48
5.45
5.43
5.10
4.20
4.12
4.01
3.85
2.08
2.07
1.97
1.95
1.92
1.61
1.57
1.50
1.49
1.28
0.90
0.88
0.86
0.83
0.83
0.80
4.58
3.58
1.79
18.03
8.45
3.13
f1 (ppm)

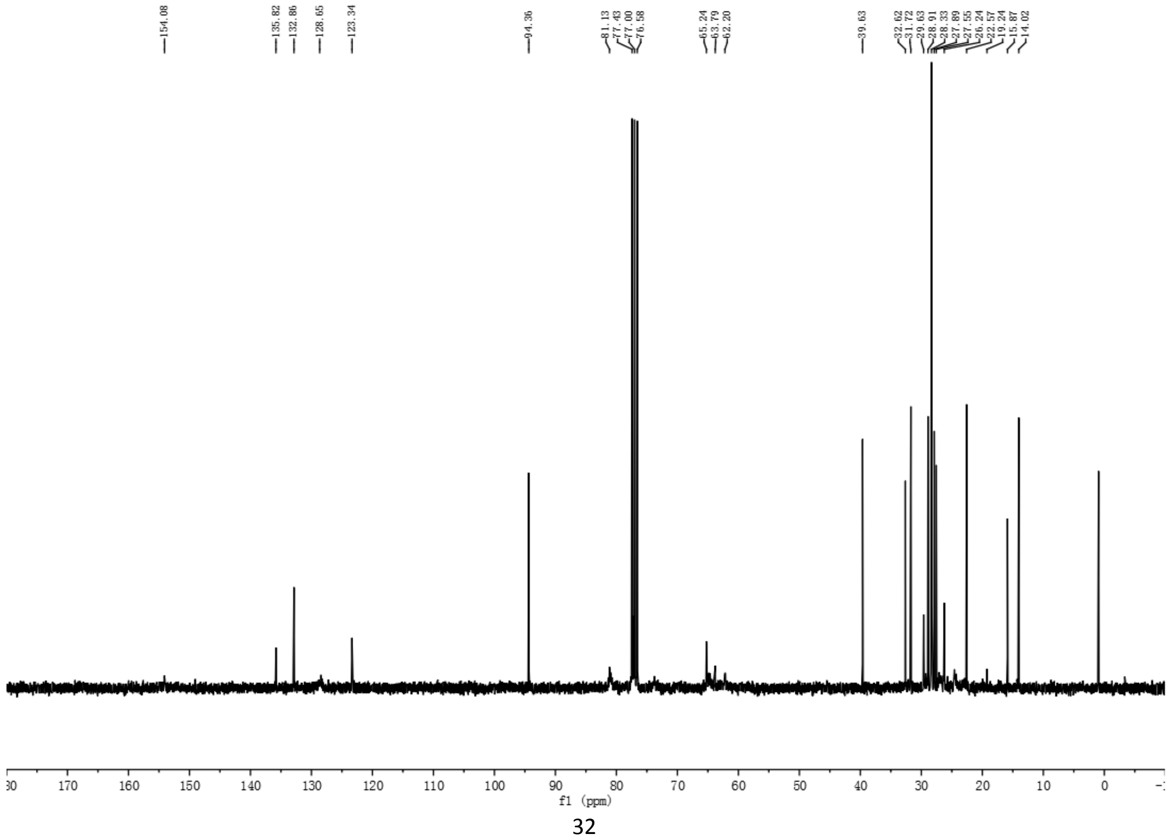
154.08
135.82
132.86
128.65
123.34
94.36
81.13
77.43
77.00
76.58
65.24
63.79
62.20
39.63
32.62
31.72
29.63
28.91
28.33
27.89
27.55
26.24
22.57
19.24
15.87
14.02
f1 (ppm)

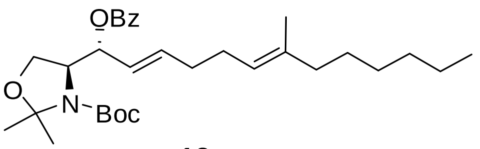

18

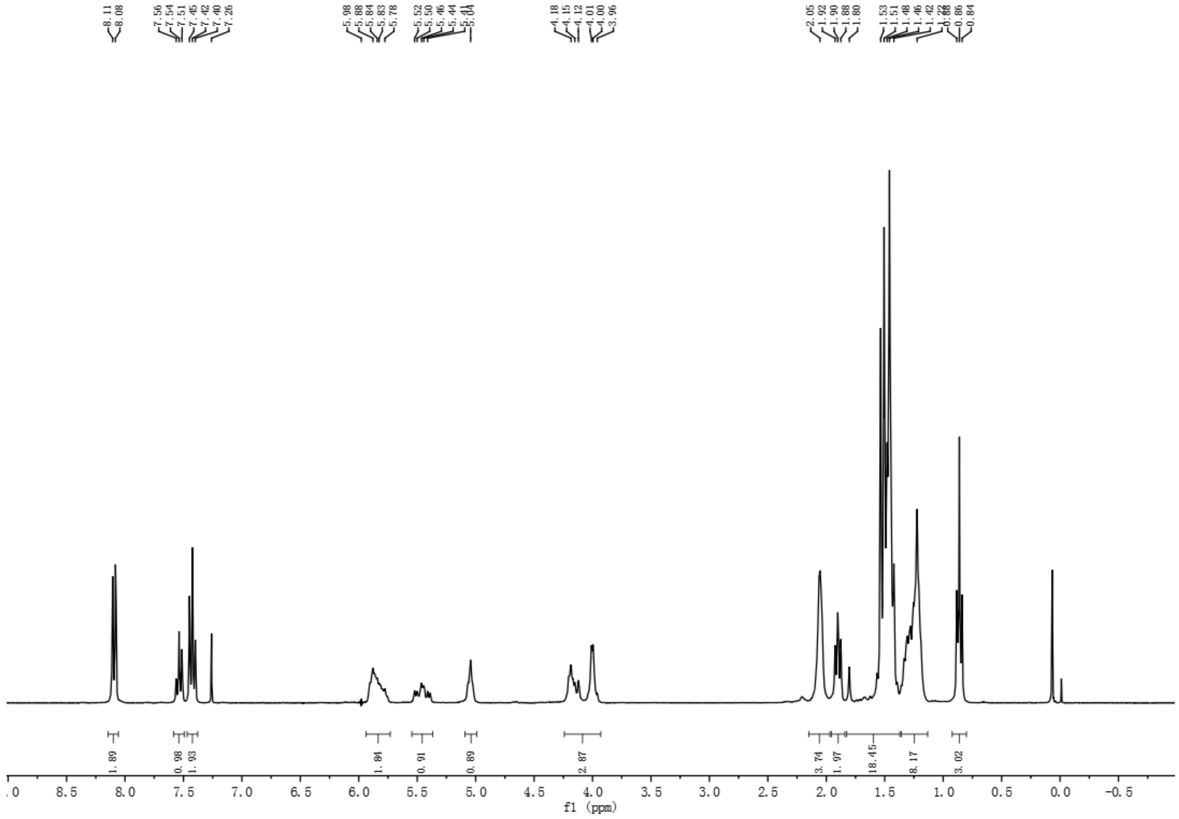

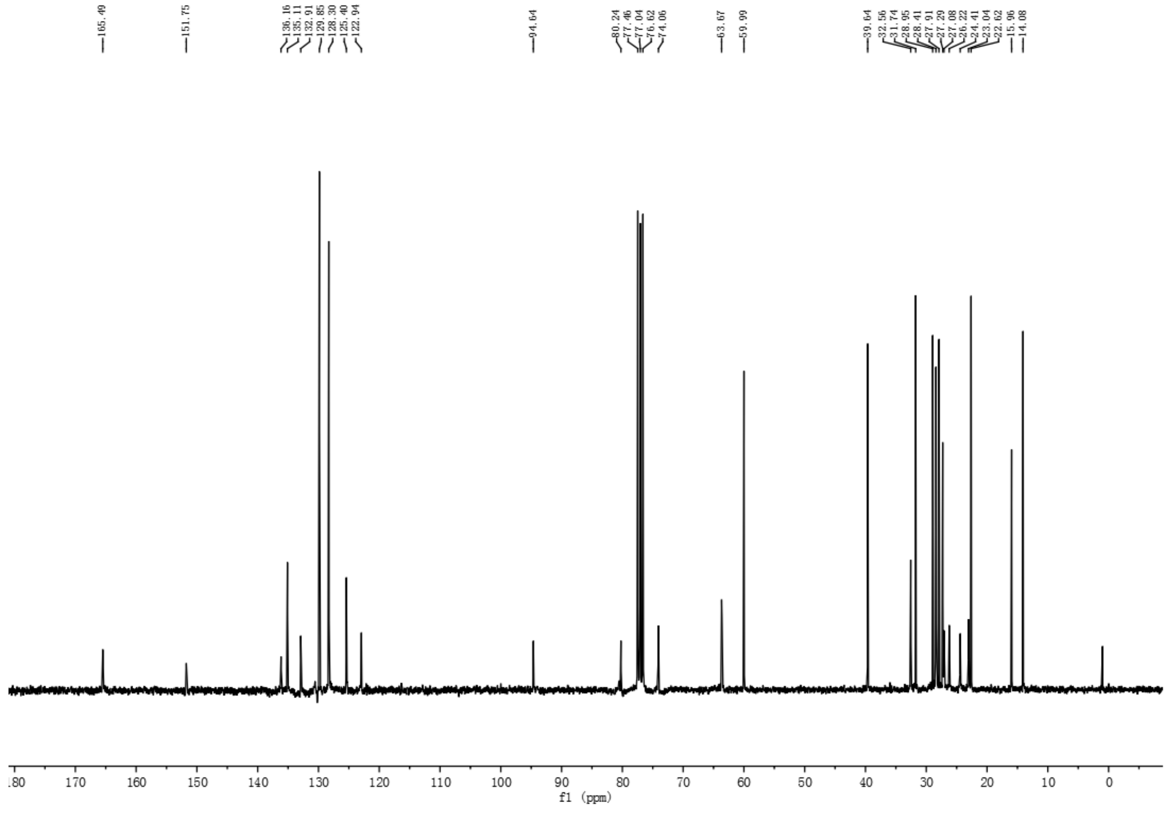

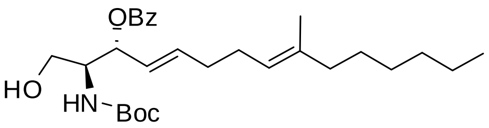

19

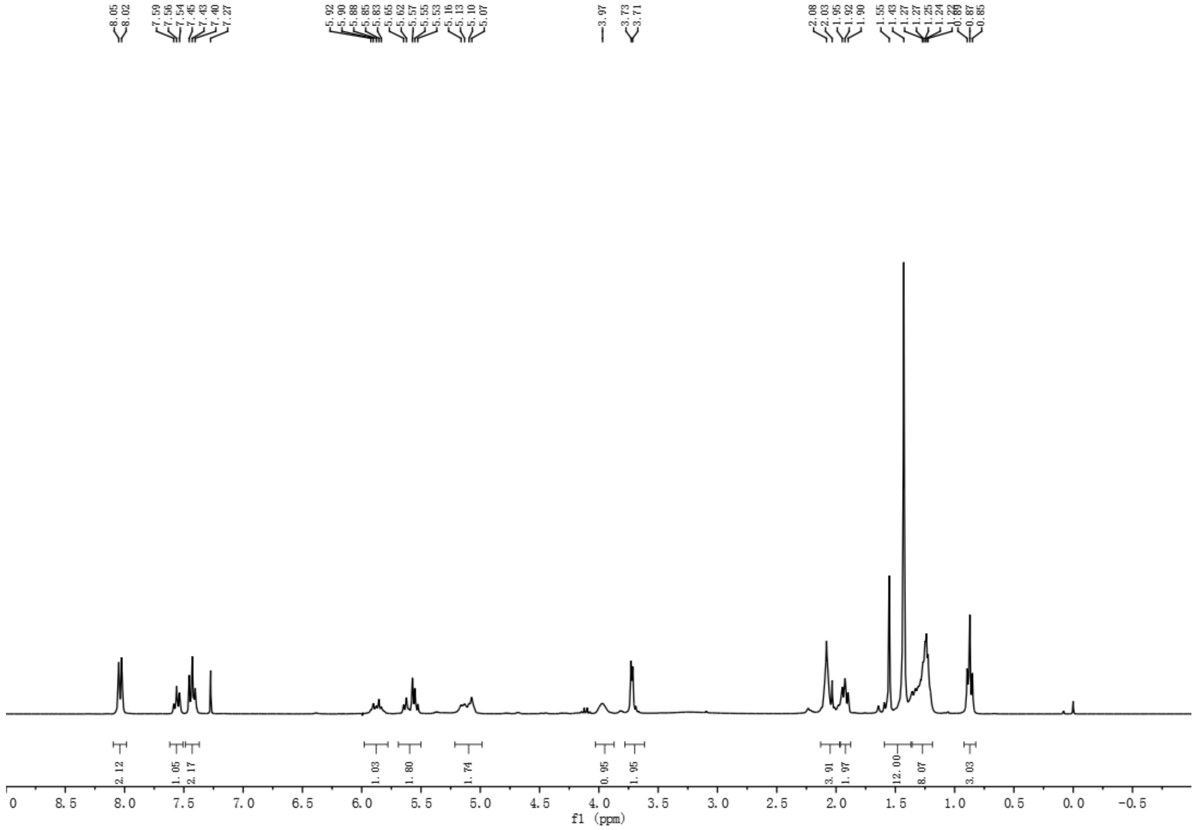

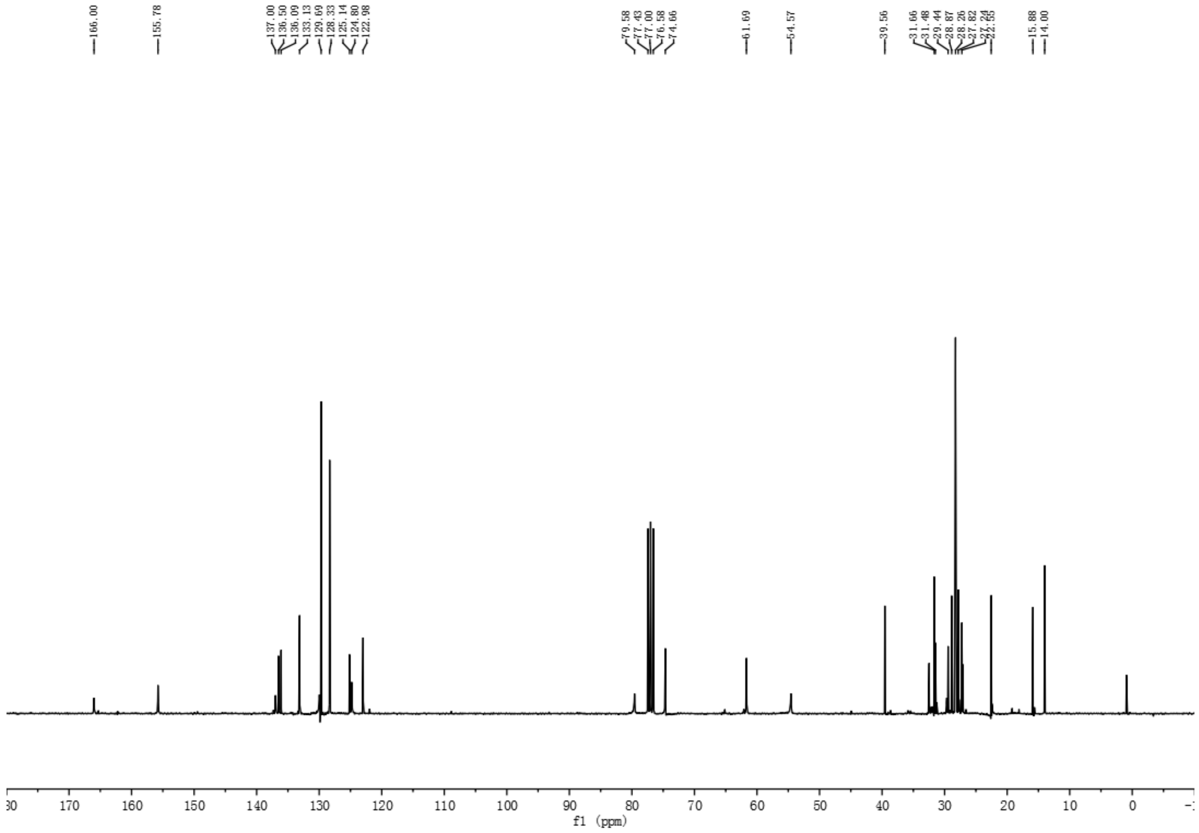

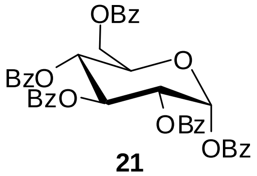
OBz
BzO
BzO
O
OBz
OBz

21

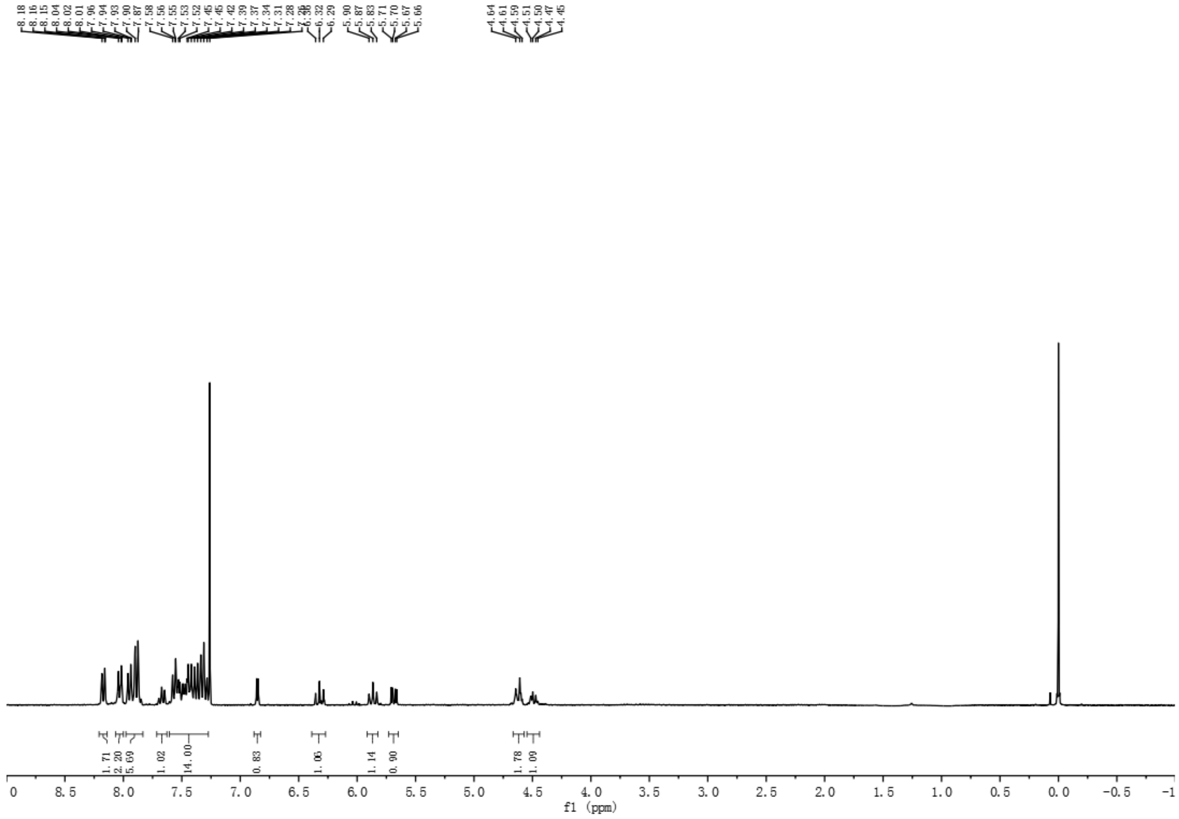
8.18 8.16 8.15 8.04 8.02 8.01 7.96 7.94 7.93 7.90 7.87 7.58 7.56 7.55 7.53 7.52 7.45 7.45 7.42 7.39 7.37 7.34 7.31 7.28 6.32 6.29 5.90 5.87 5.83 5.71 5.70 5.67 5.66 4.64 4.61 4.59 4.51 4.50 4.47 4.45
1.71 2.20 5.69 1.02 14.00 0.83 1.06 1.14 0.90 1.78 1.00
f1 (ppm)

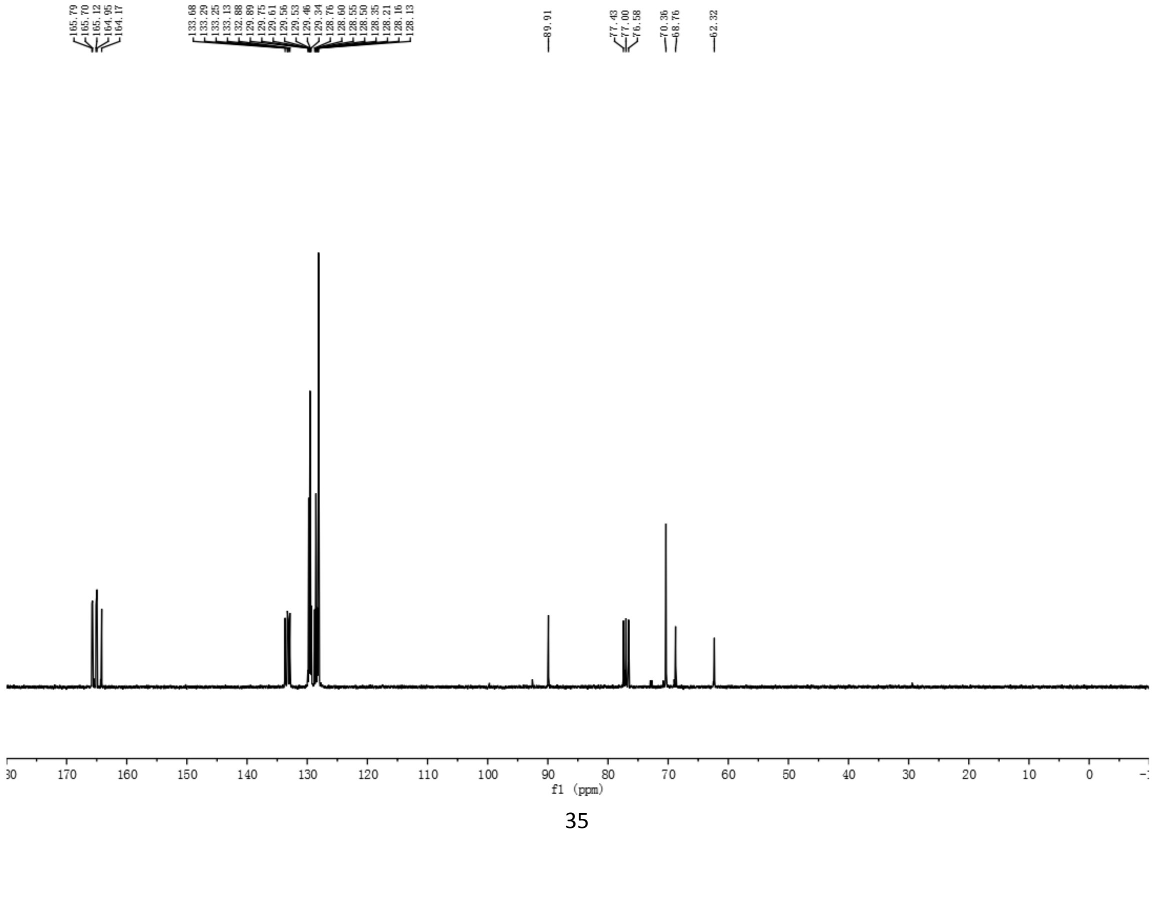
165.79 165.70 165.12 164.95 164.17 133.68 133.29 133.25 133.13 132.88 129.89 129.75 129.61 129.56 129.53 129.46 129.34 128.76 128.60 128.55 128.50 128.35 128.21 128.16 128.13 89.91 77.43 77.00 76.58 70.36 68.76 62.32
f1 (ppm)

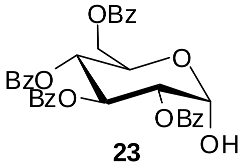
OBz
O
BzO
BzO
OBz
OH

23

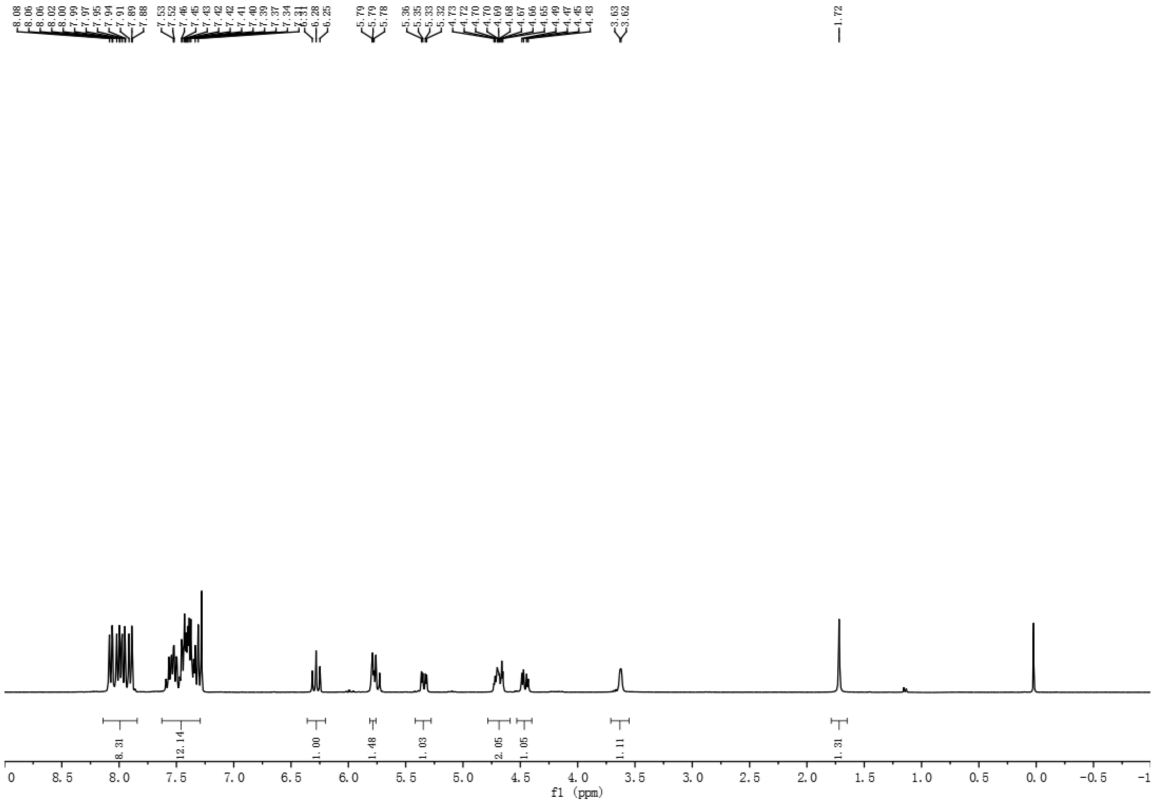
8.08 8.06 8.06 8.02 8.00 7.99 7.97 7.95 7.94 7.91 7.89 7.88 7.53 7.52 7.46 7.45 7.43 7.42 7.42 7.41 7.40 7.39 7.37 7.34 7.31 6.31 6.28 6.25 5.79 5.79 5.78 5.36 5.35 5.33 5.32 4.73 4.72 4.70 4.70 4.69 4.68 4.67 4.66 4.65 4.49 4.47 4.45 4.43 3.63 3.62 1.72
8.31
12.14
1.00
1.48
1.03
2.05
1.05
1.11
1.31
f1 (ppm)

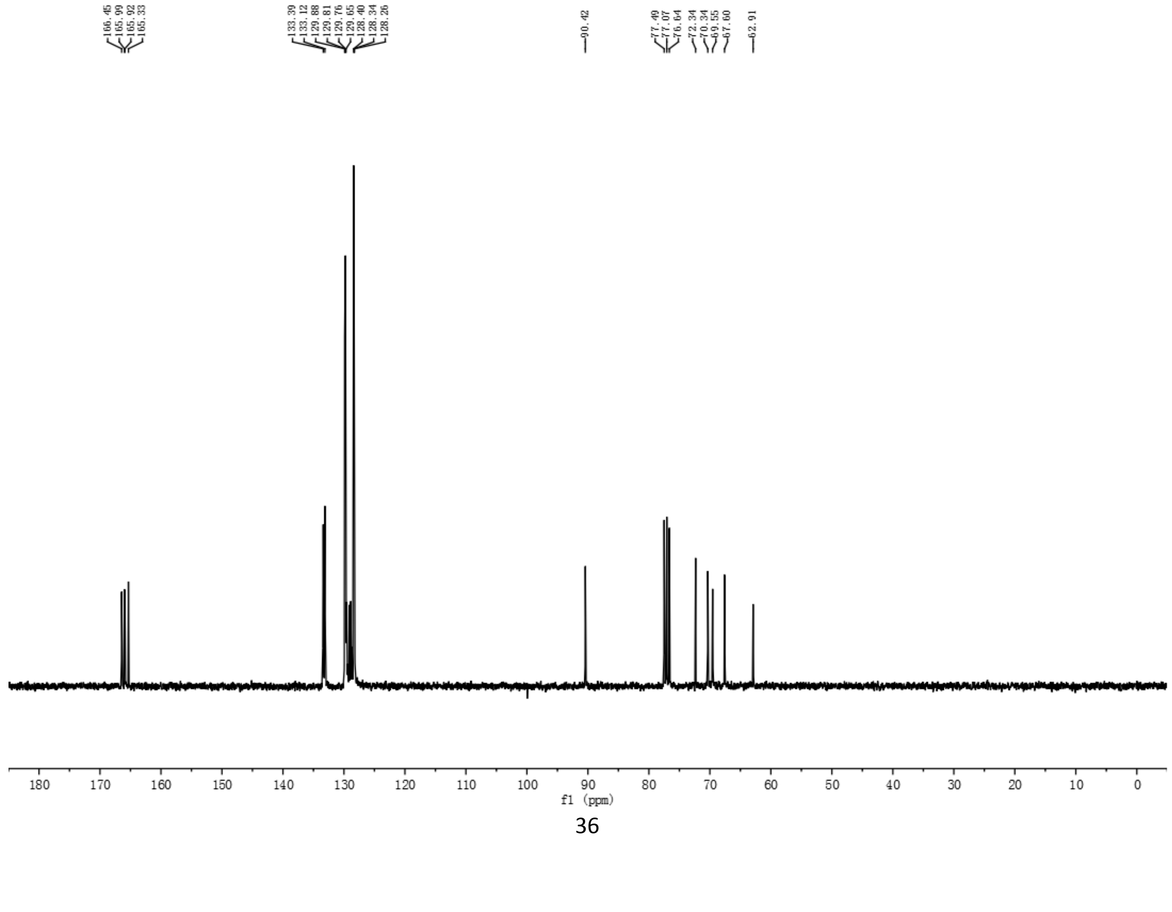
166.45 165.99 165.92 165.33
133.39 133.12 129.88 129.81 129.76 129.65 128.40 128.34 128.26
90.42
77.49 77.07 76.64 72.34 70.34 69.55 67.60
62.91
f1 (ppm)

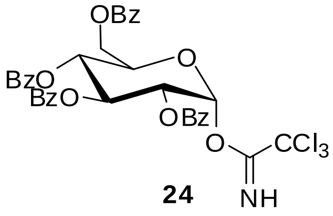
OBz
O
BzO
BzO
OBz
O
CCl3
NH

24

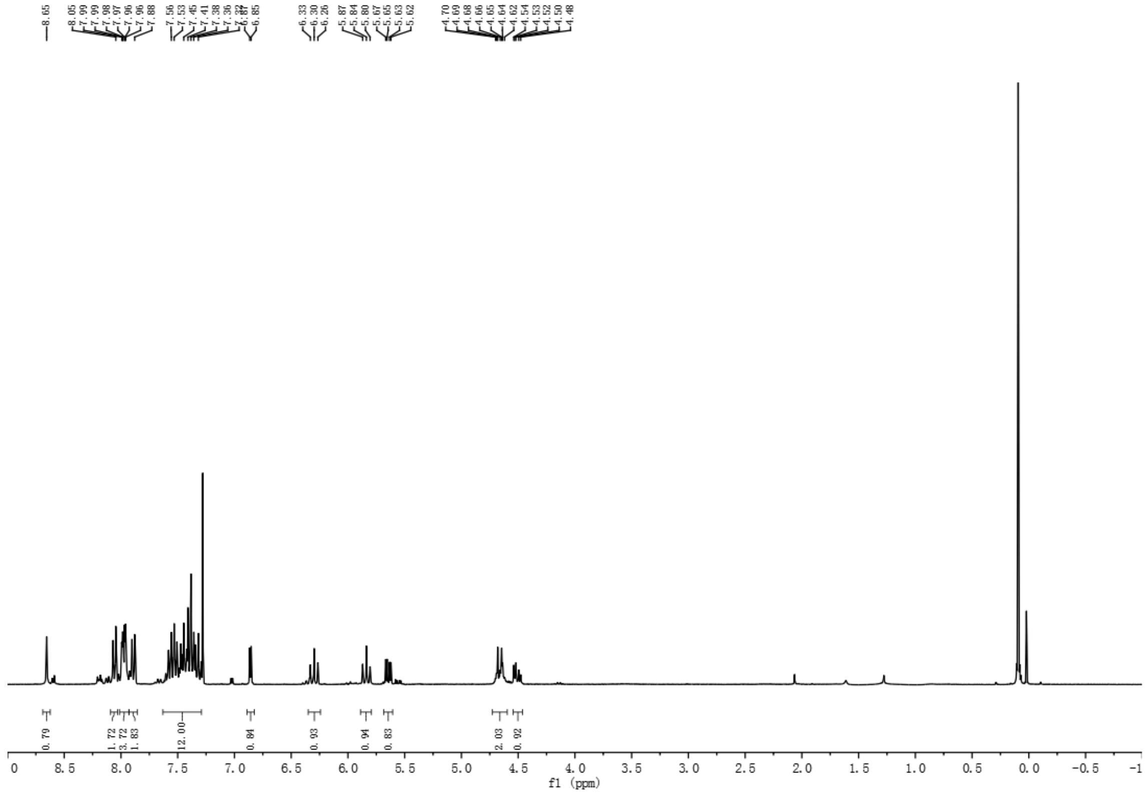
8.65
8.05
7.99
7.99
7.98
7.97
7.96
7.96
7.88
7.56
7.53
7.45
7.41
7.38
7.36
6.87
6.85
6.33
6.30
6.26
5.87
5.84
5.80
5.67
5.65
5.63
5.62
4.70
4.69
4.68
4.66
4.65
4.64
4.62
4.54
4.53
4.52
4.50
4.48
0.79
1.72
3.72
1.83
12.00
0.84
0.93
0.94
0.83
2.03
0.92
f1 (ppm)

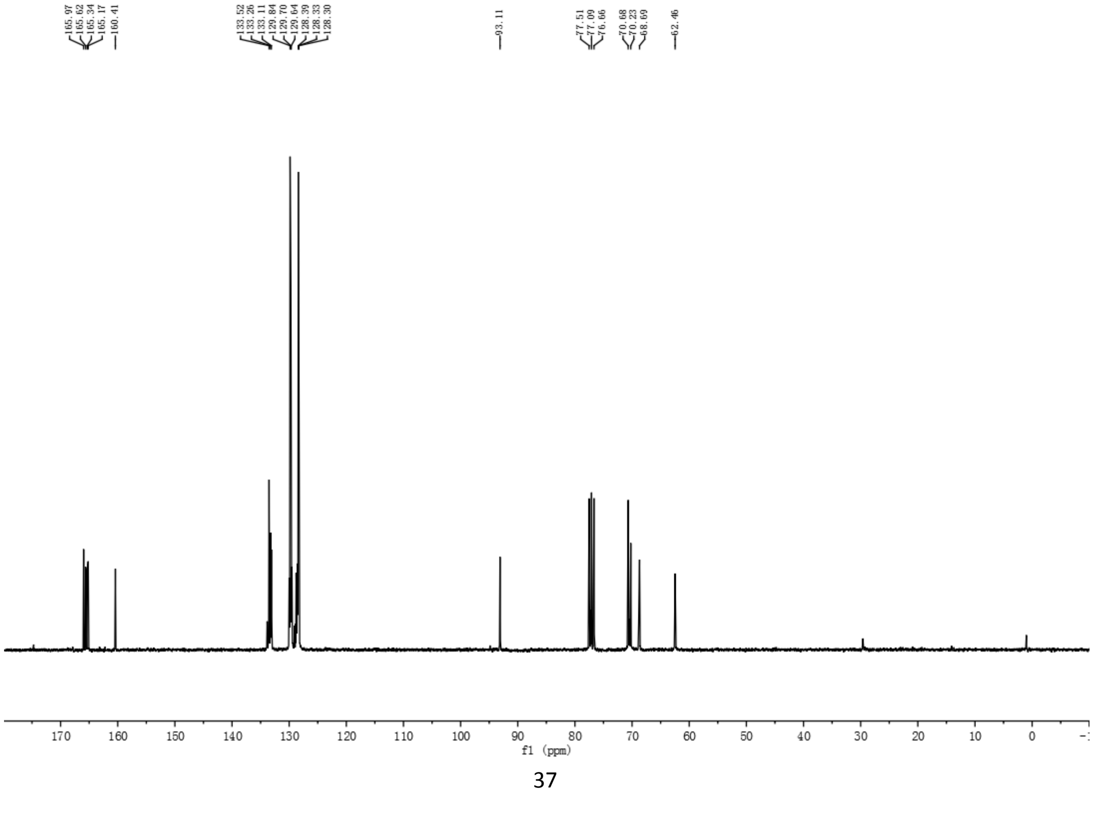
165.97
165.62
165.34
165.17
160.41
133.52
133.26
133.11
129.84
129.70
129.64
128.39
128.33
128.30
93.11
77.51
77.09
76.66
70.68
70.23
68.69
62.46
f1 (ppm)

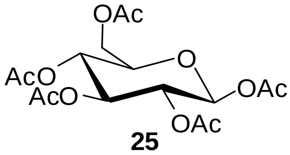
OAc
AcO
AcO
O
OAc
OAc

25

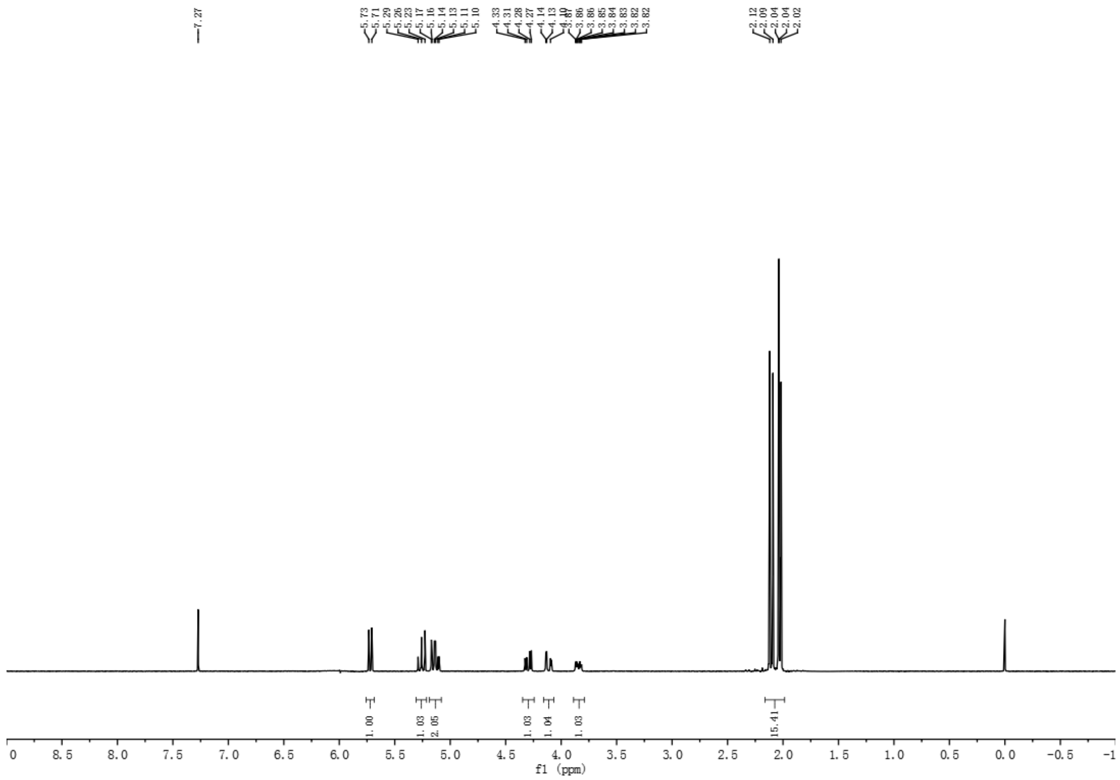
7.27
5.73 5.71 5.29 5.26 5.23 5.17 5.16 5.14 5.13 5.11 5.10
4.33 4.31 4.28 4.27 4.14 4.13 4.10 3.87 3.86 3.86 3.85 3.84 3.83 3.82 3.82
2.12 2.09 2.04 2.04 2.02
1.00 1.03 2.05 1.03 1.04 1.03 15.41
f1 (ppm)

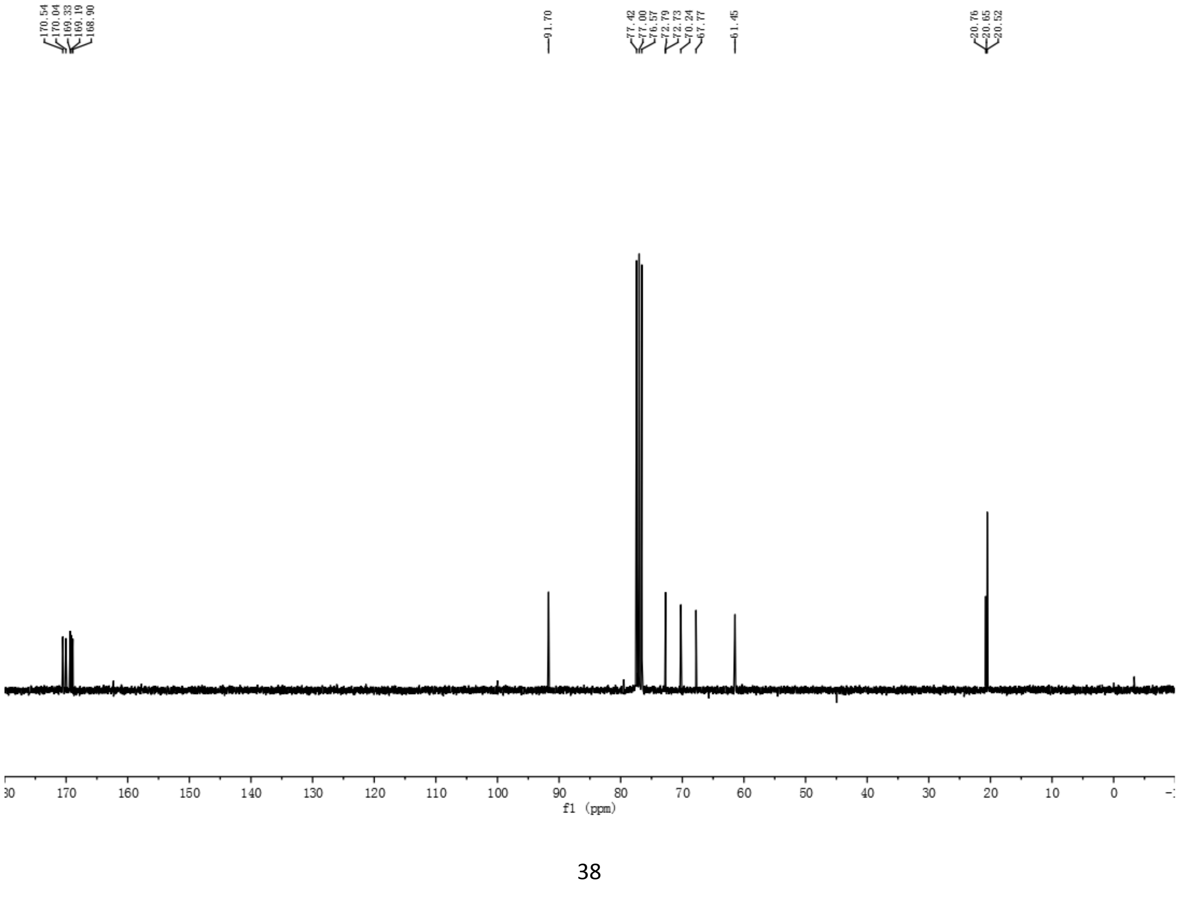
170.54 170.04 169.33 169.19 168.90
91.70
77.42 77.00 76.57 72.79 72.73 70.24 67.77
61.45
20.76 20.65 20.52
f1 (ppm)

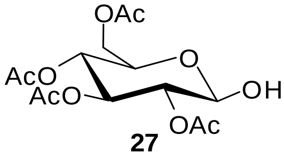
OAc
AcO
AcO
O
OH
OAc

27

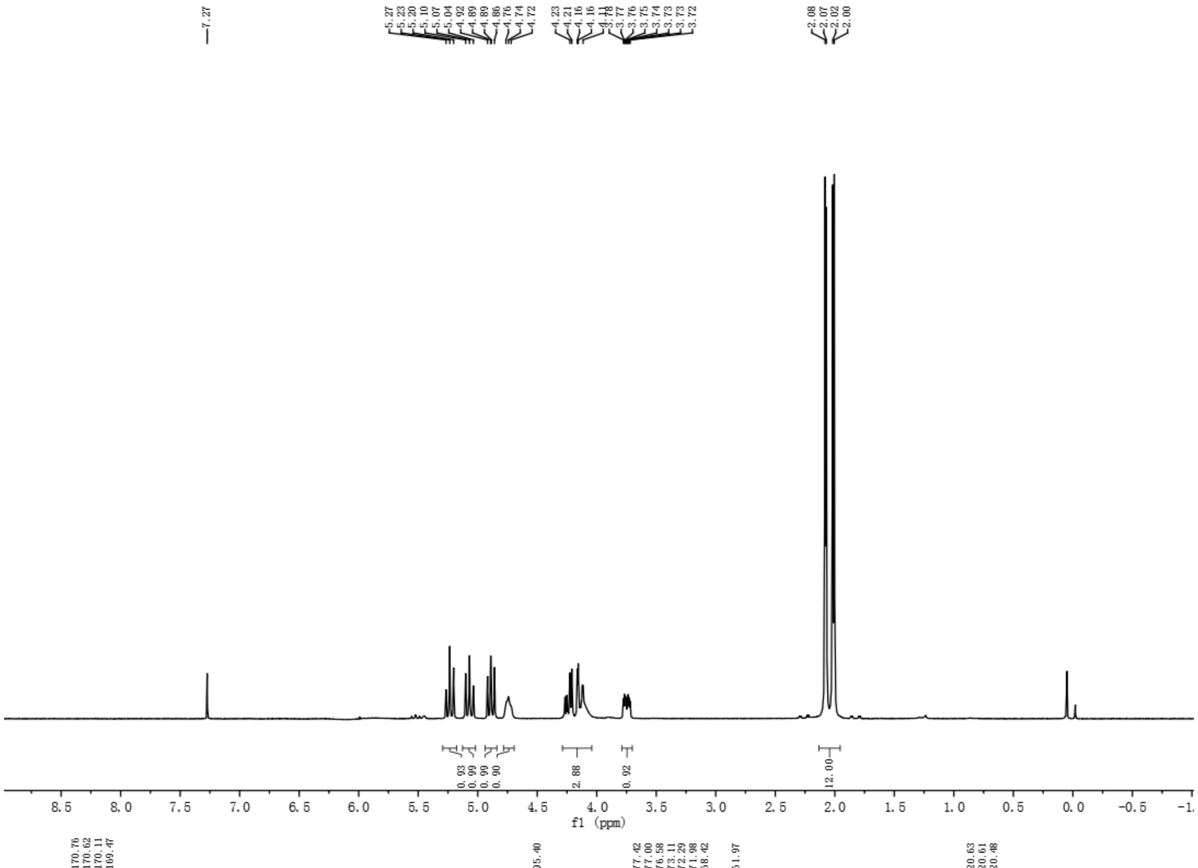
7.27
5.27
5.23
5.20
5.10
5.07
5.04
4.92
4.89
4.89
4.86
4.76
4.74
4.72
4.23
4.21
4.16
4.16
4.11
3.78
3.77
3.76
3.75
3.74
3.73
3.73
3.72
2.08
2.07
2.02
2.00
0.93
0.99
0.99
0.90
2.88
0.92
12.00
f1 (ppm)

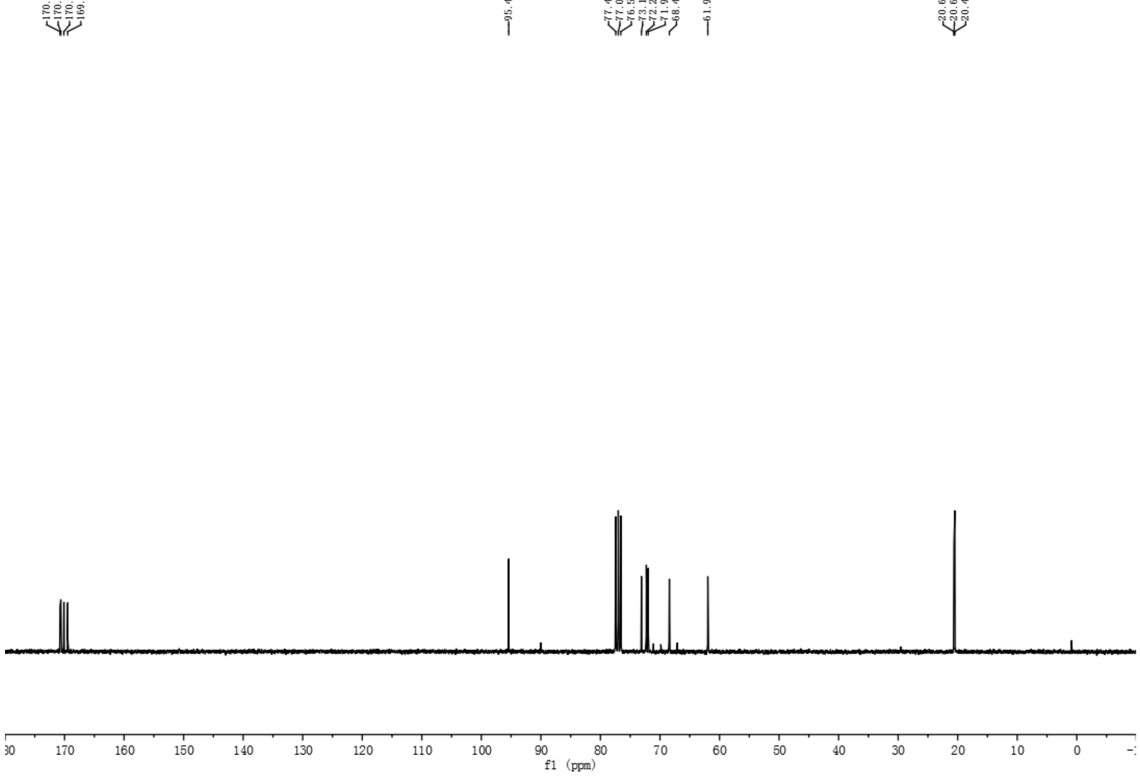
170.76
170.62
170.11
169.47
95.40
77.42
77.00
76.58
73.11
72.29
71.98
68.42
61.97
20.63
20.61
20.48
f1 (ppm)

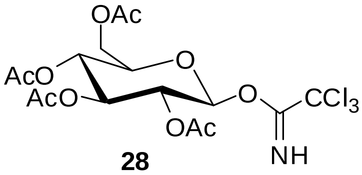
OAc
AcO
AcO
O
O
CCl3
OAc
NH

28

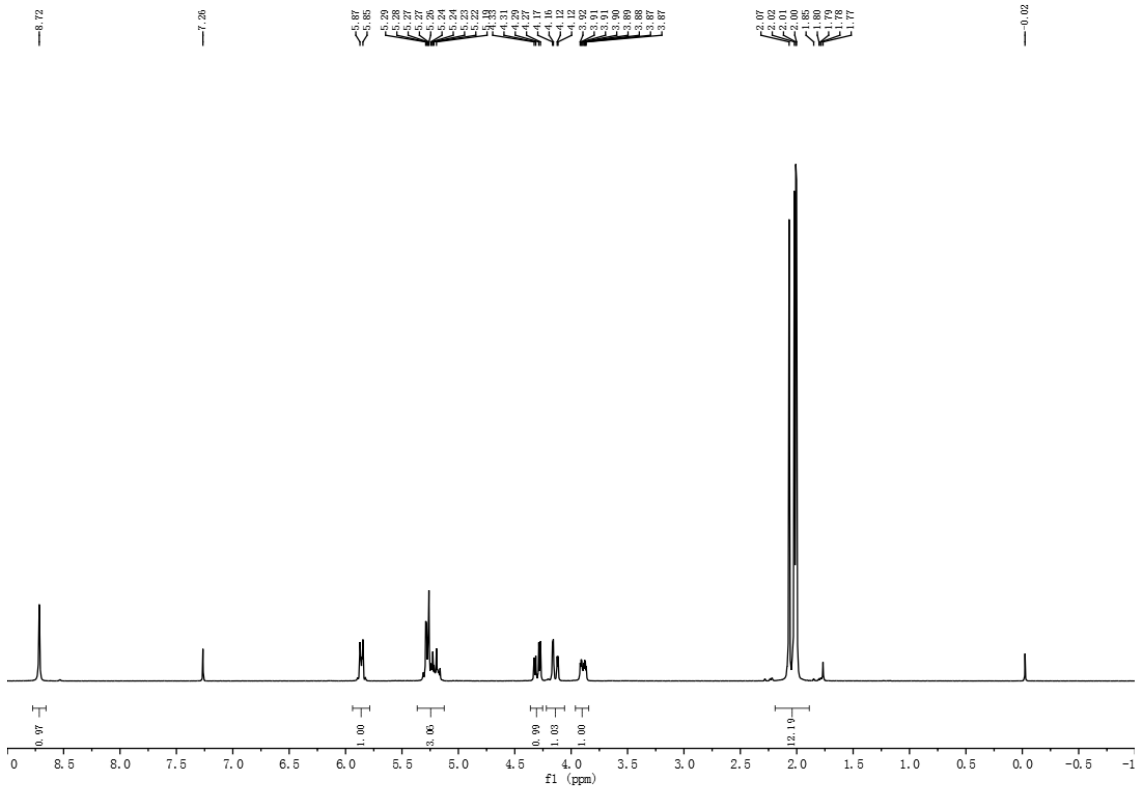
8.72
7.26
5.87
5.85
5.29
5.28
5.27
5.27
5.26
5.24
5.24
5.23
5.22
5.19
4.33
4.31
4.29
4.27
4.17
4.16
4.12
4.12
3.92
3.91
3.91
3.90
3.89
3.88
3.87
3.87
2.07
2.02
2.01
2.00
1.85
1.80
1.79
1.78
1.77
0.02
0.97
1.00
3.05
0.99
1.03
1.00
12.19
f1 (ppm)

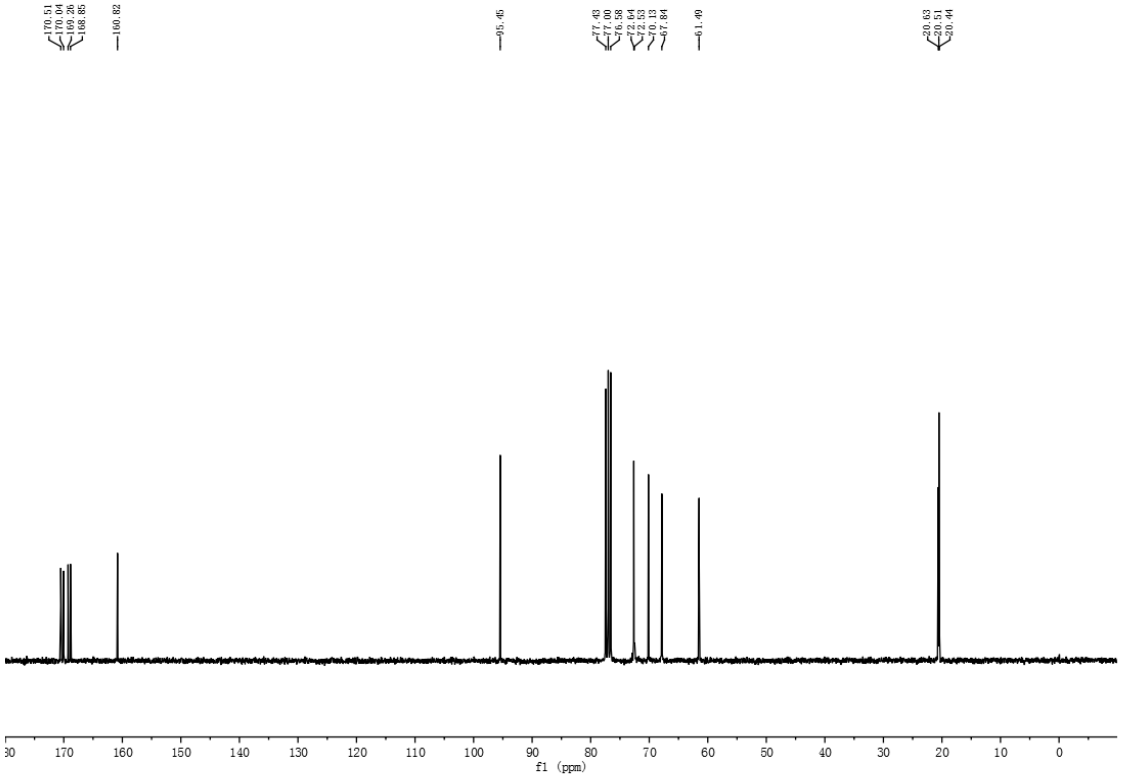
170.51
170.04
169.26
168.85
160.82
95.45
77.43
77.00
76.58
72.64
72.53
70.13
67.84
61.49
20.63
20.51
20.44
f1 (ppm)

$I-C_{15}H_{31}$

29

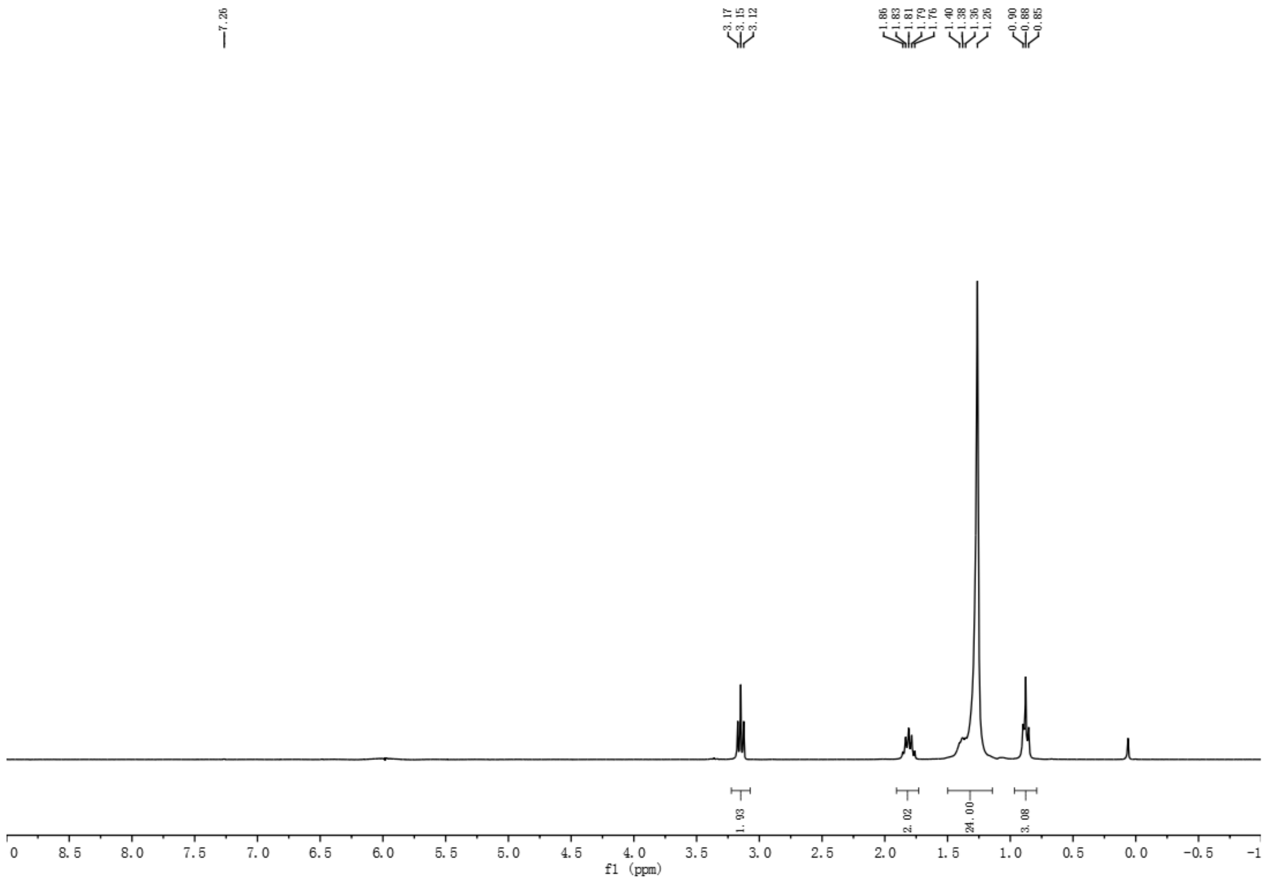
7.26
3.17
3.15
3.12
1.86
1.83
1.81
1.79
1.76
1.40
1.38
1.36
1.26
0.90
0.88
0.85
1.93
2.02
24.00
3.08
f1 (ppm)

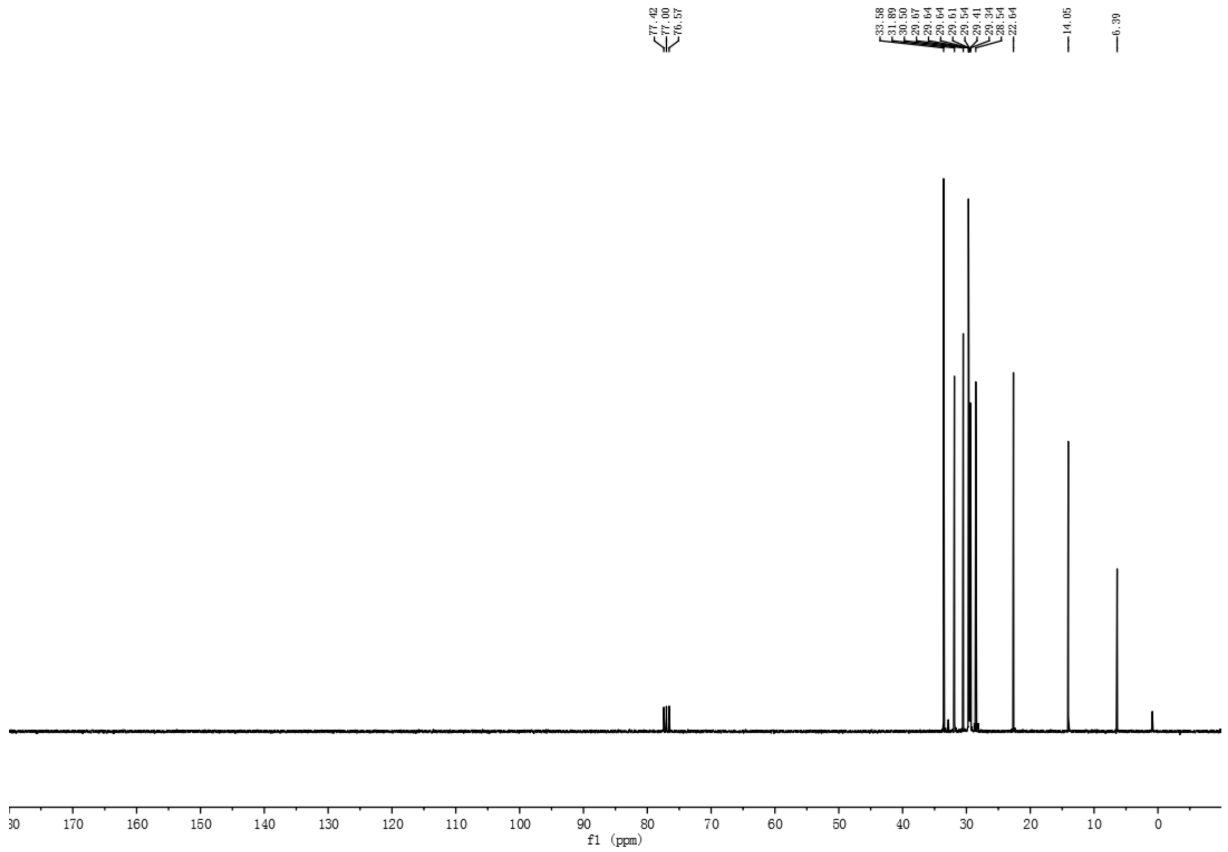
77.42
77.00
76.57
33.58
31.89
30.50
29.67
29.64
29.61
29.54
29.41
29.34
28.54
22.64
14.05
6.39
f1 (ppm)

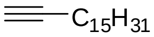
$C_{15}H_{31}$

30

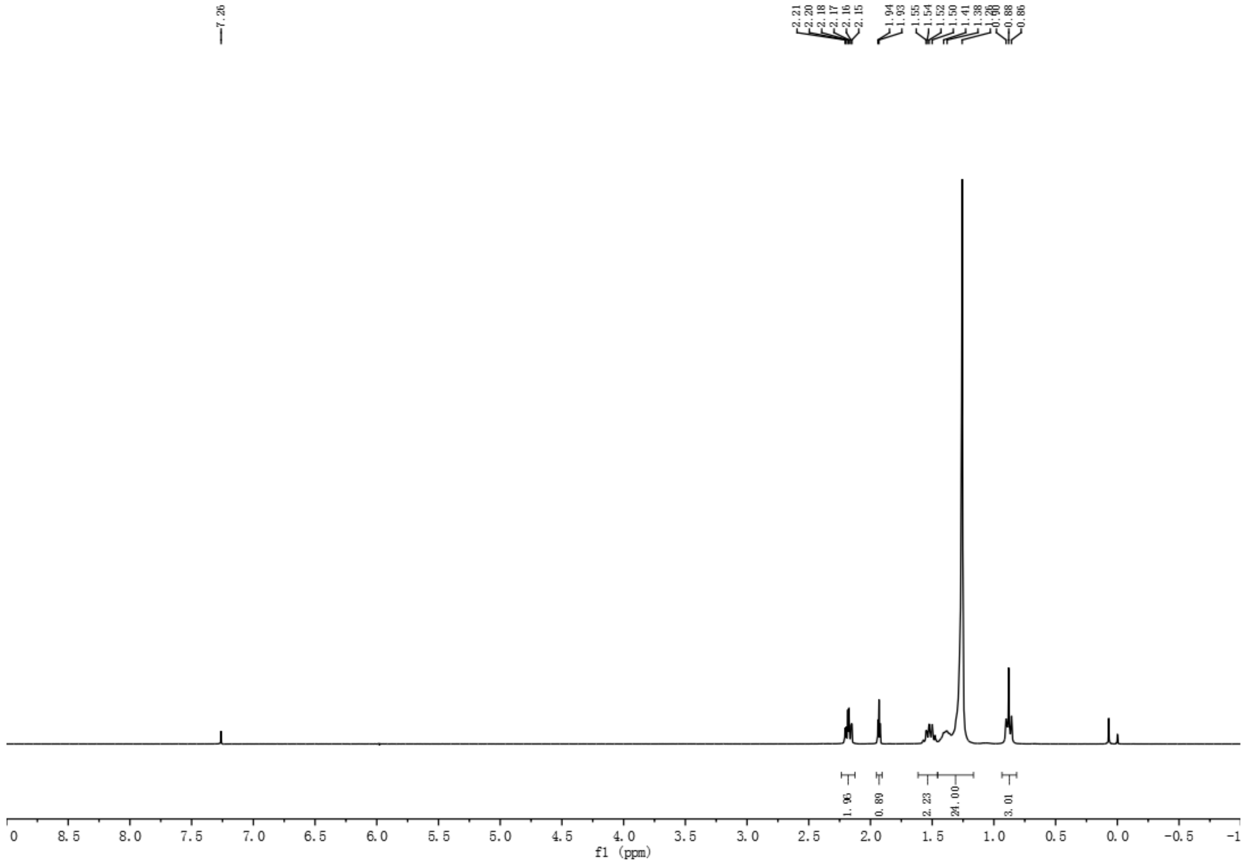
7.26
2.21
2.20
2.18
2.17
2.16
2.15
1.94
1.93
1.55
1.54
1.52
1.50
1.41
1.38
1.26
0.90
0.88
0.86
1.96
0.89
2.23
24.00
3.01
f1 (ppm)

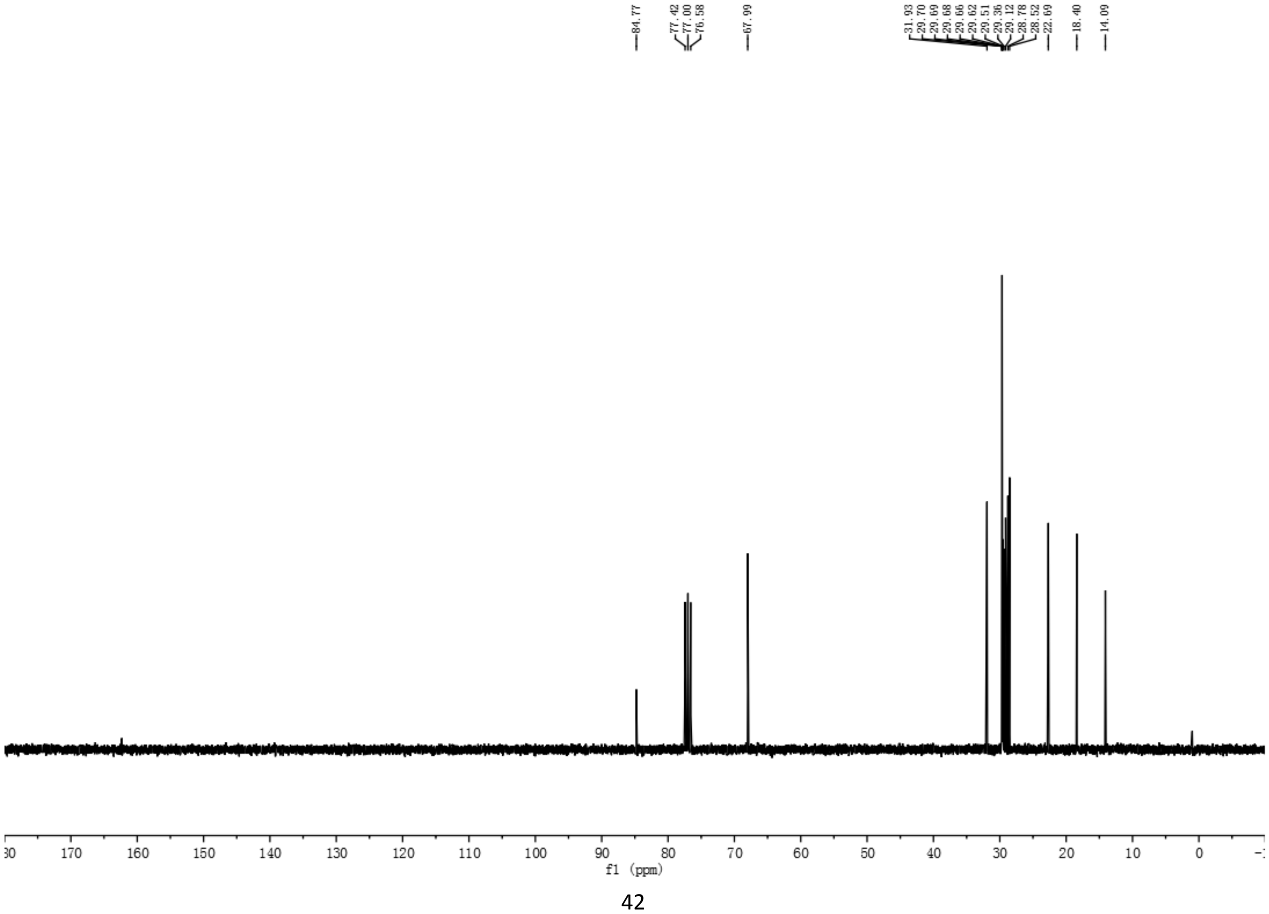
84.77
77.42
77.00
76.58
67.99
31.93
29.70
29.69
29.68
29.66
29.62
29.51
29.36
29.12
28.78
28.52
22.69
18.40
14.09
f1 (ppm)

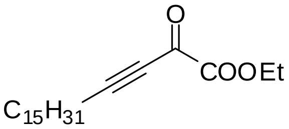

31

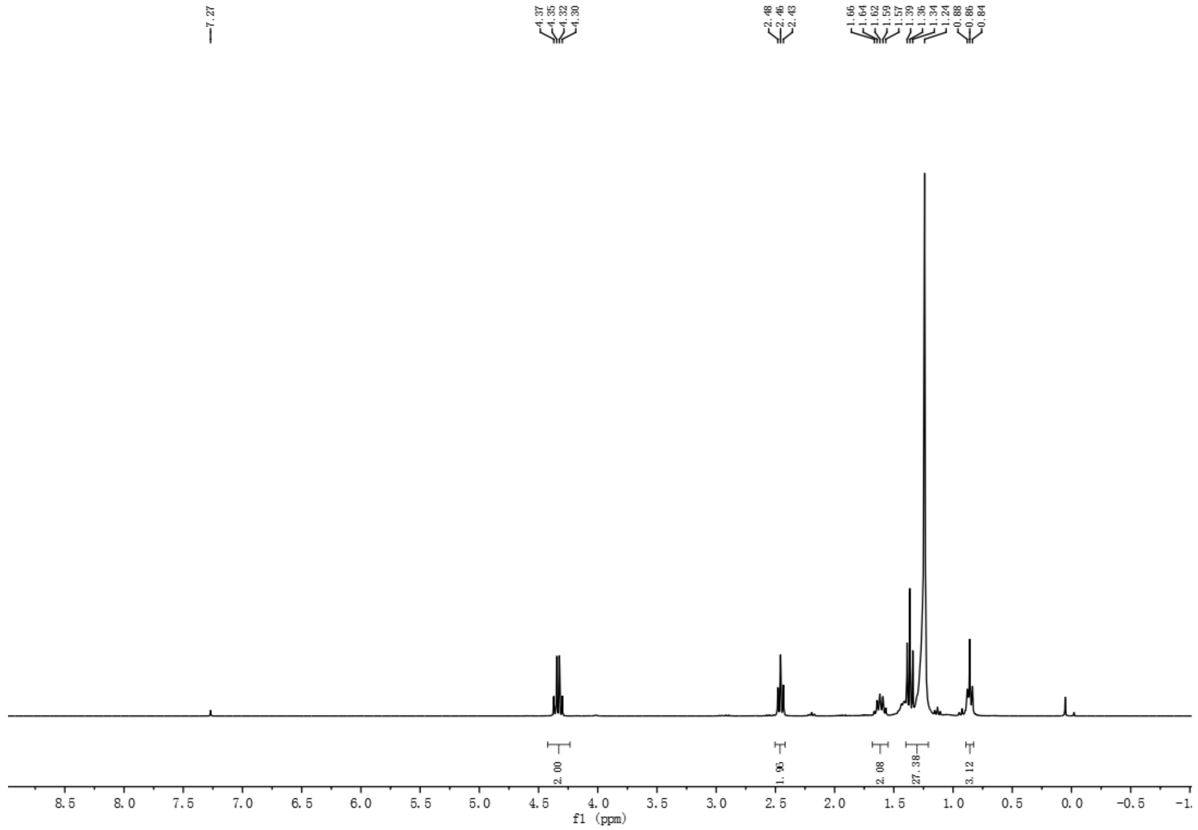

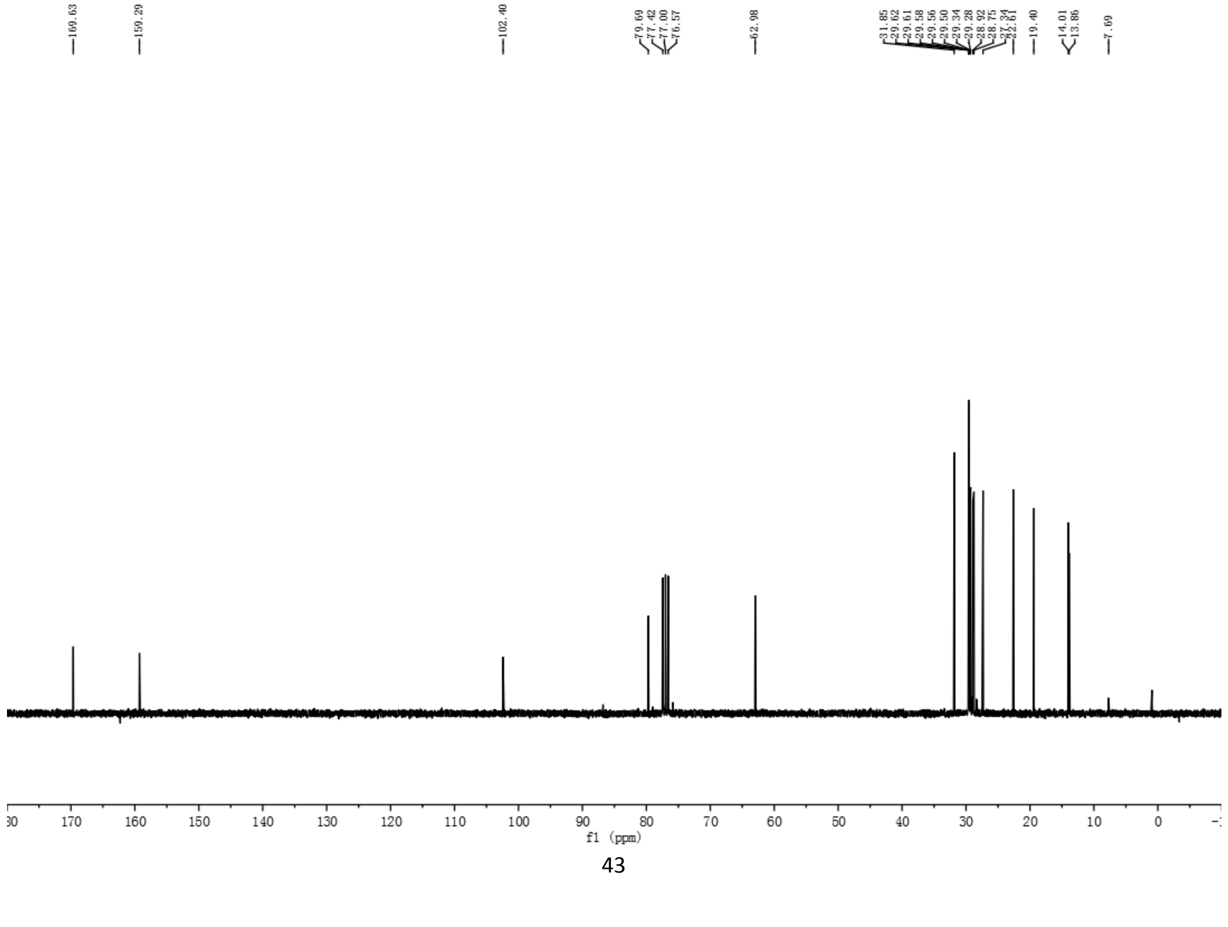

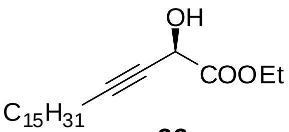

32

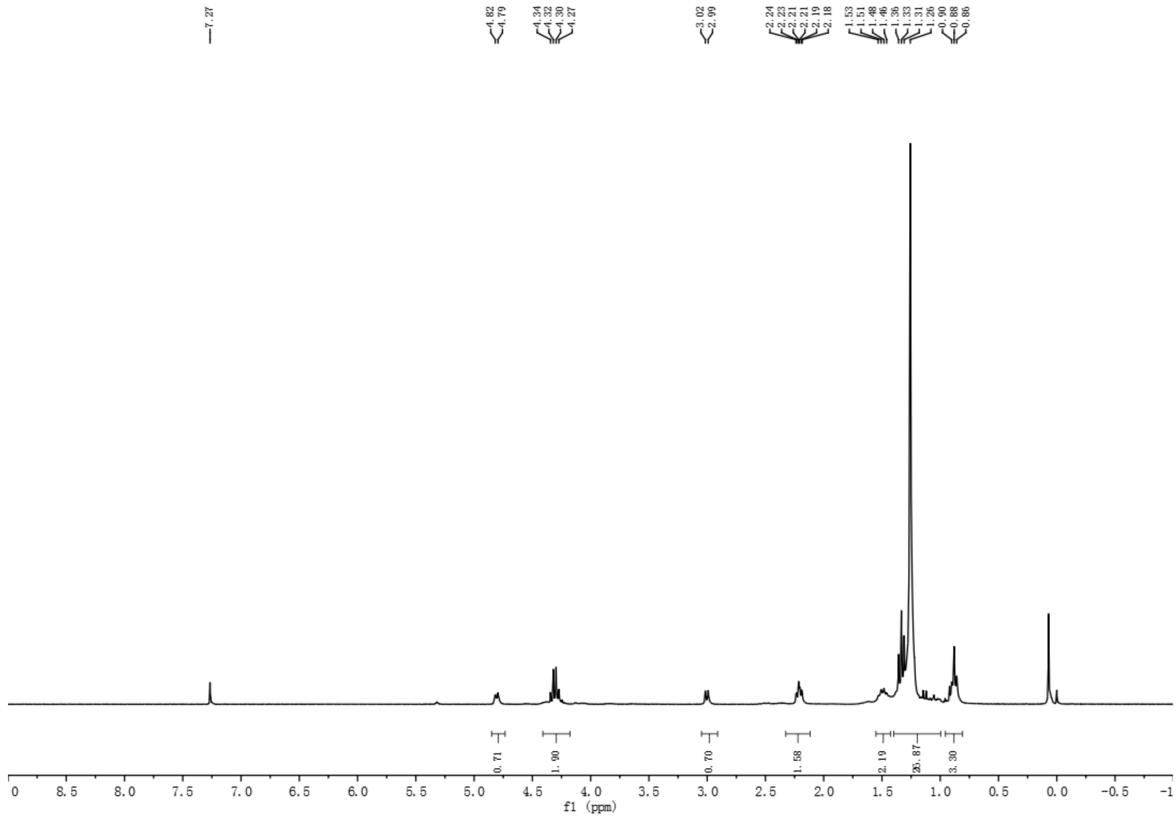

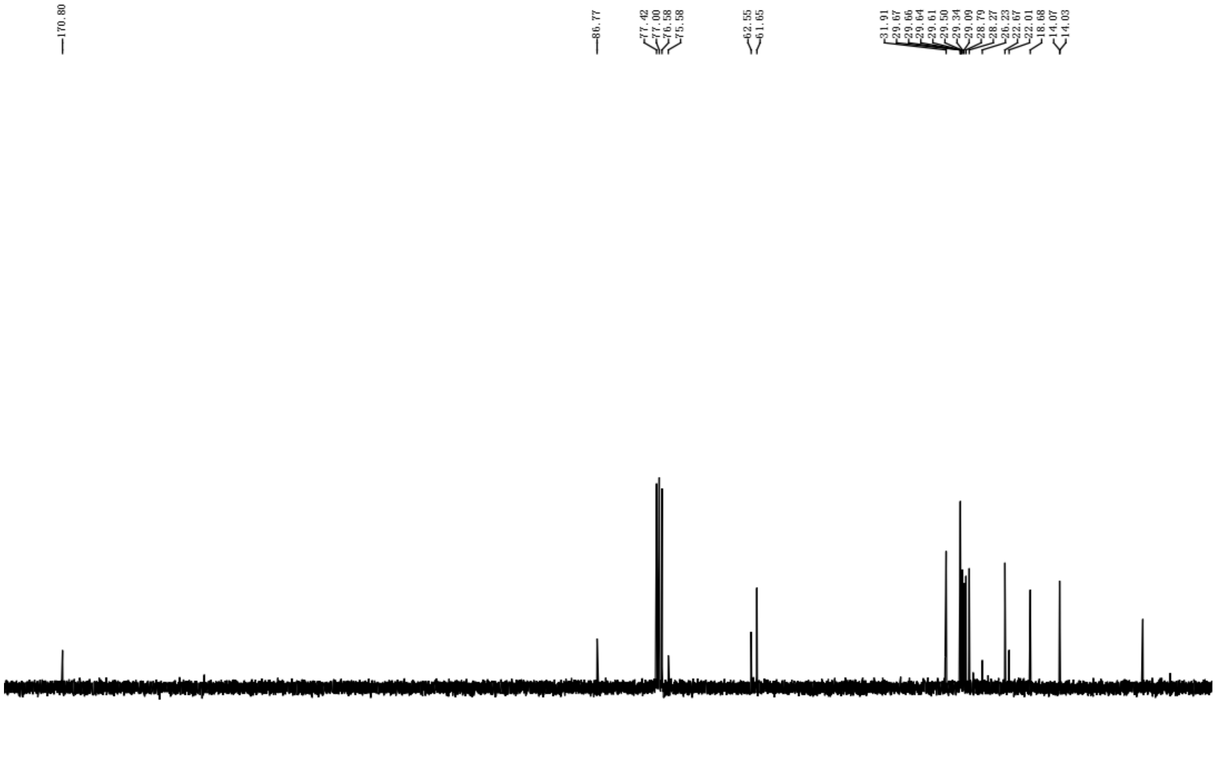

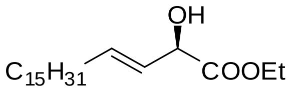

33

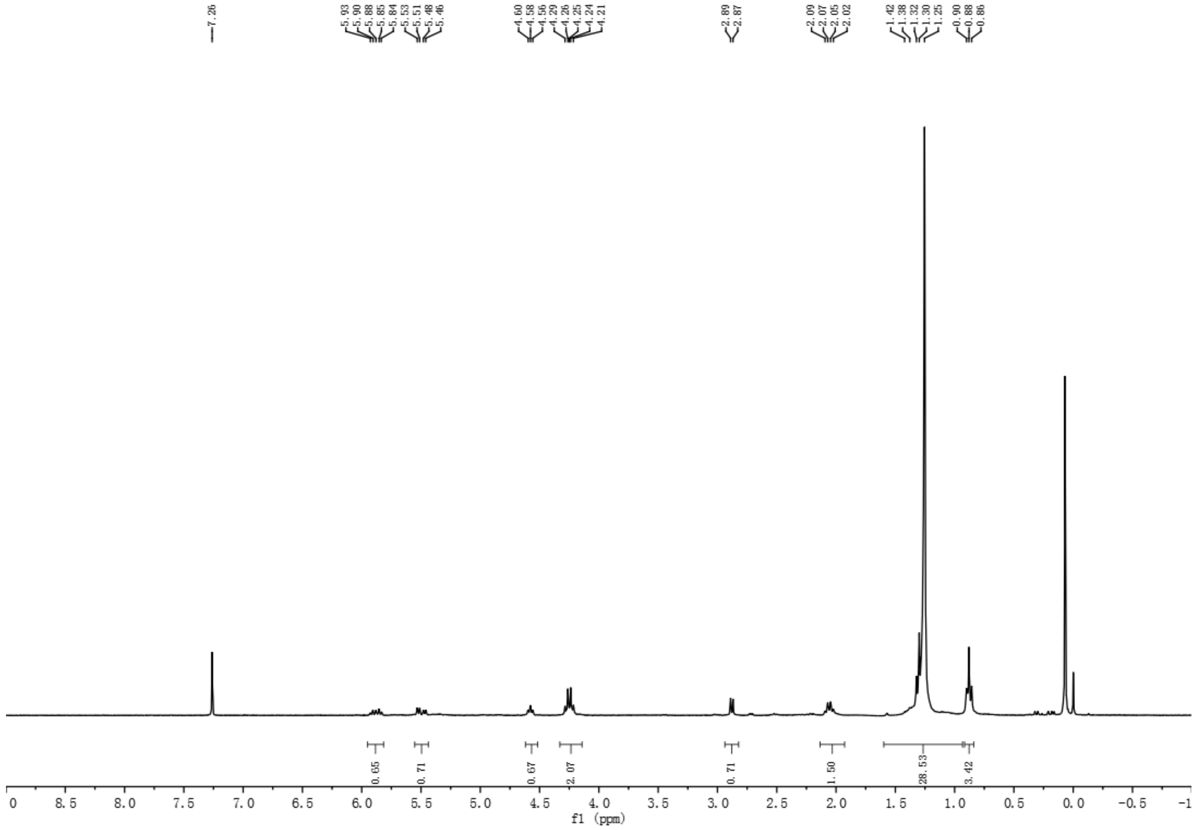

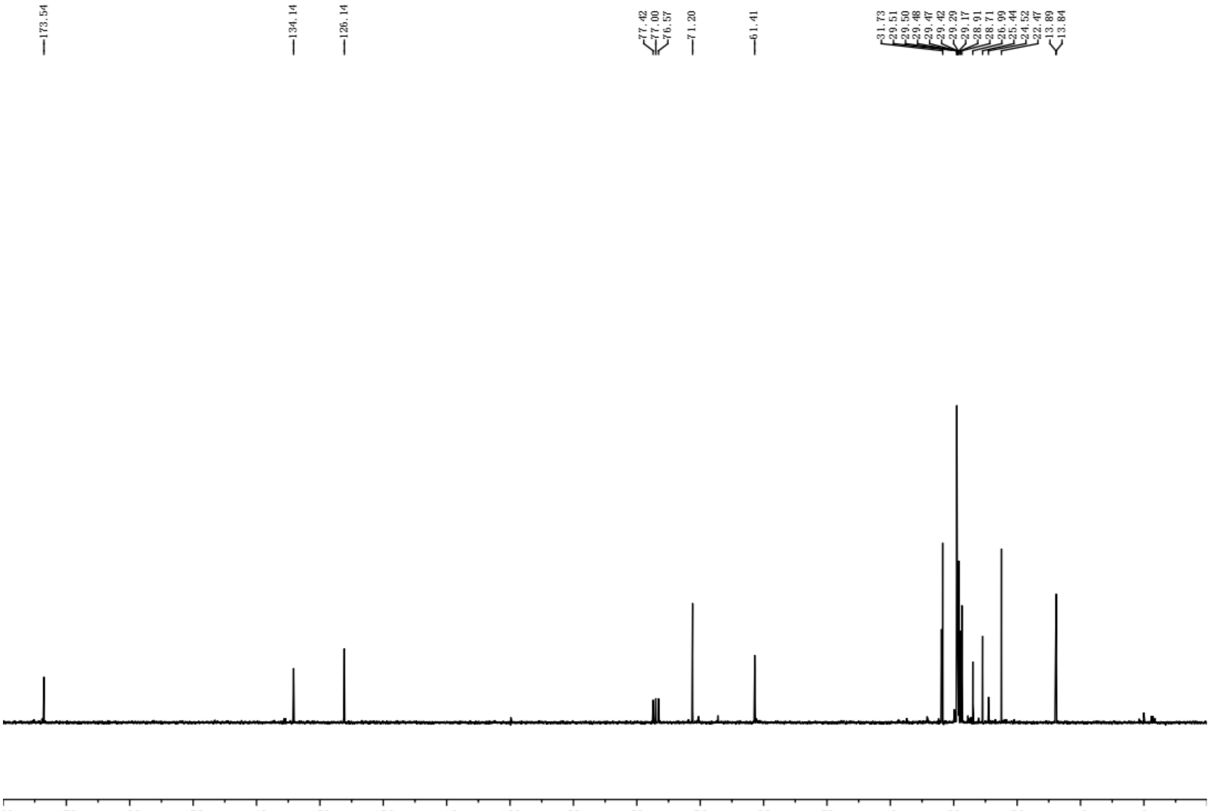

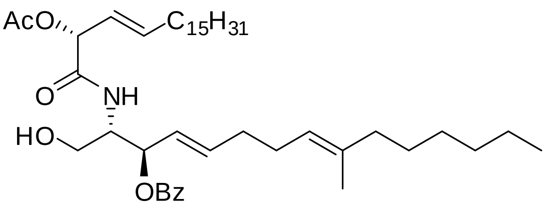

1

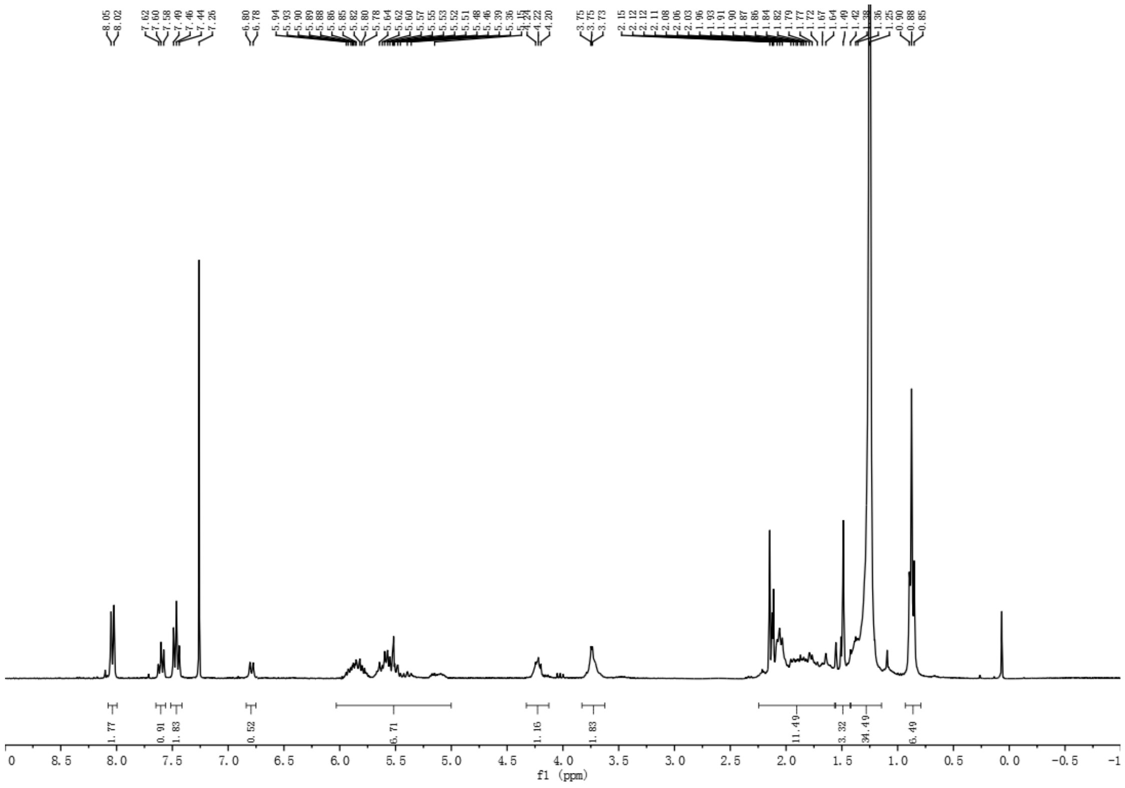

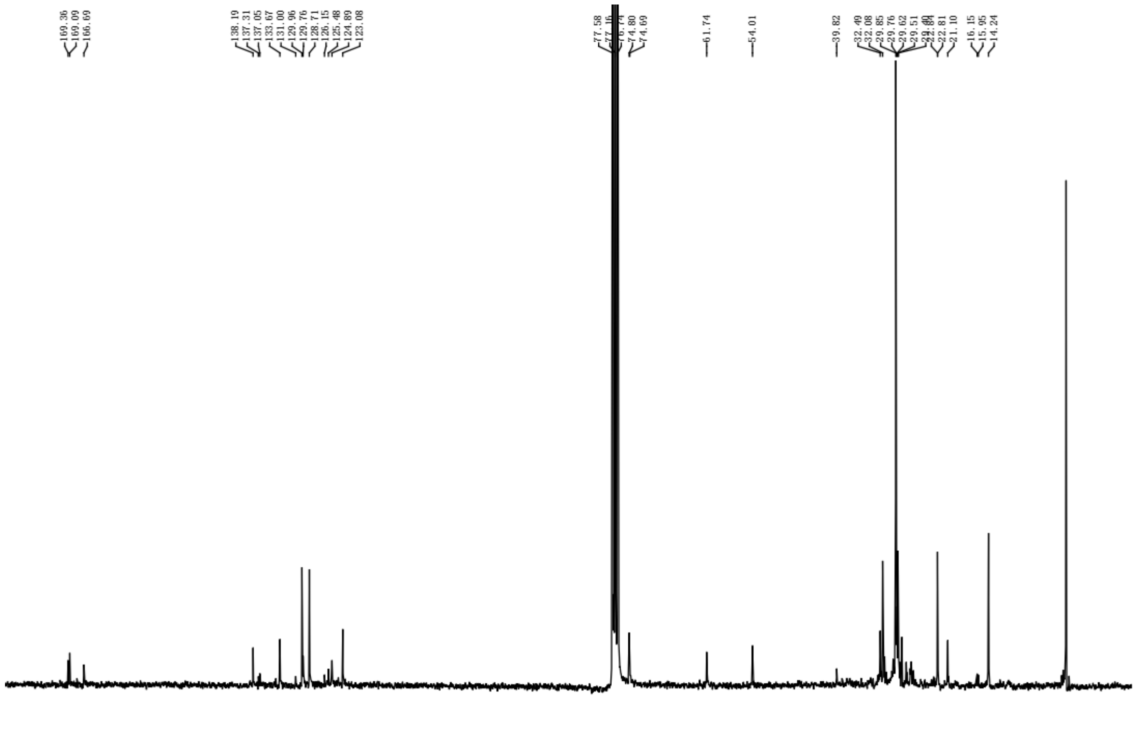

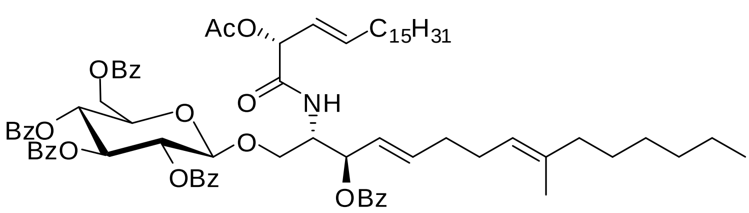

2

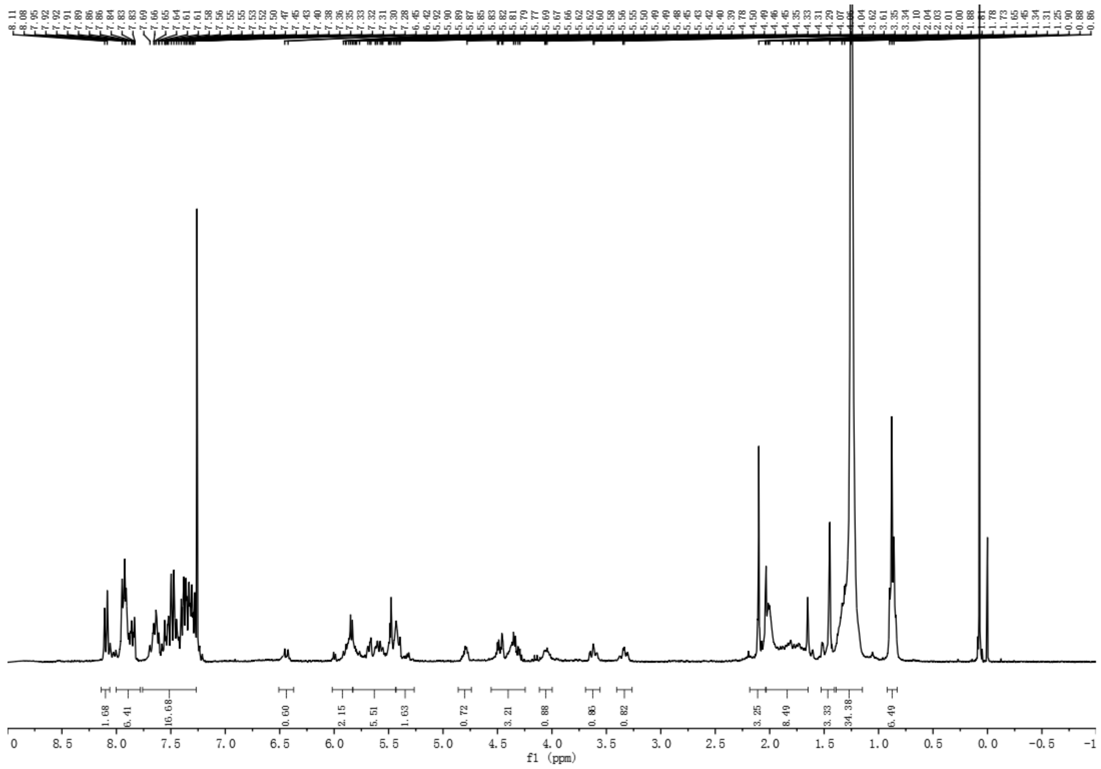

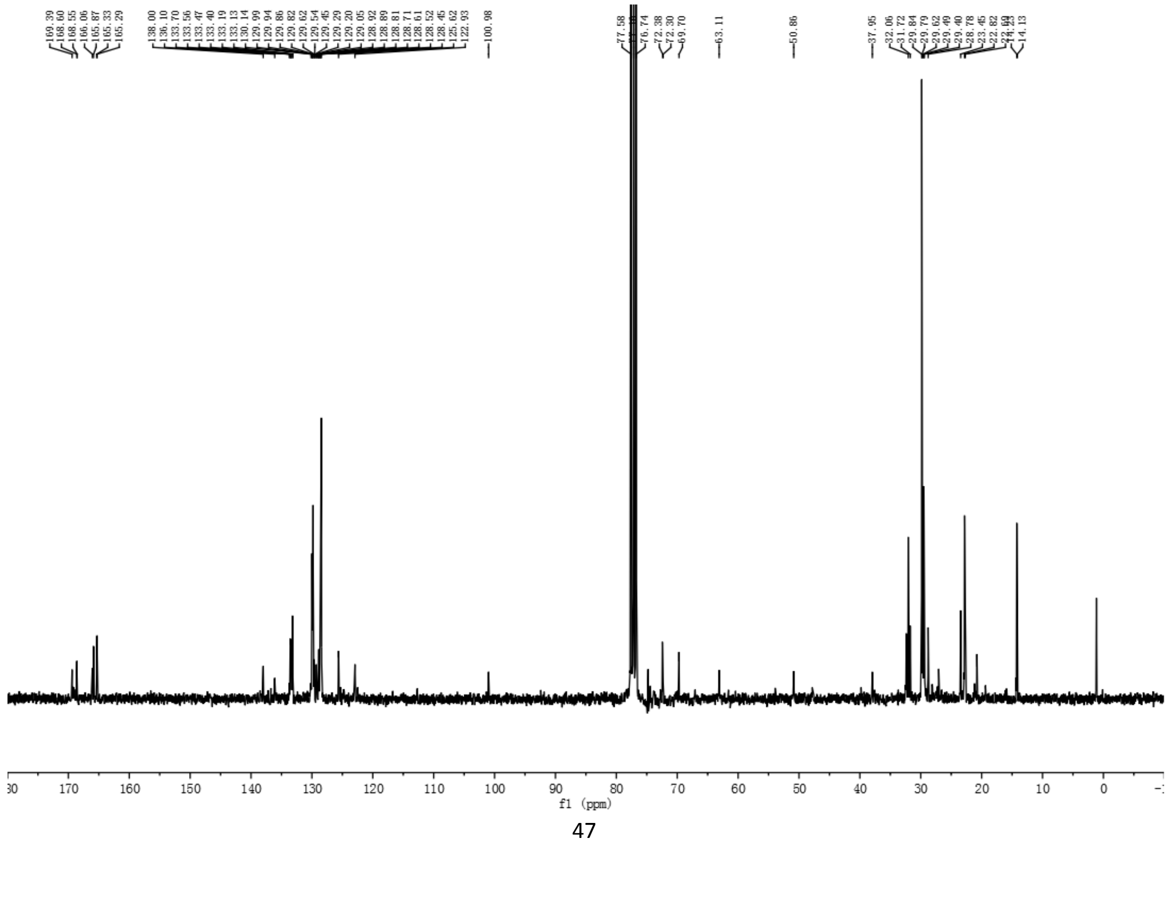

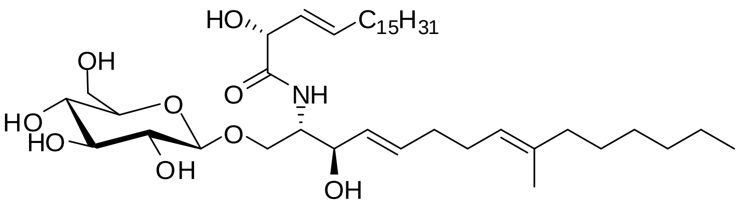

3

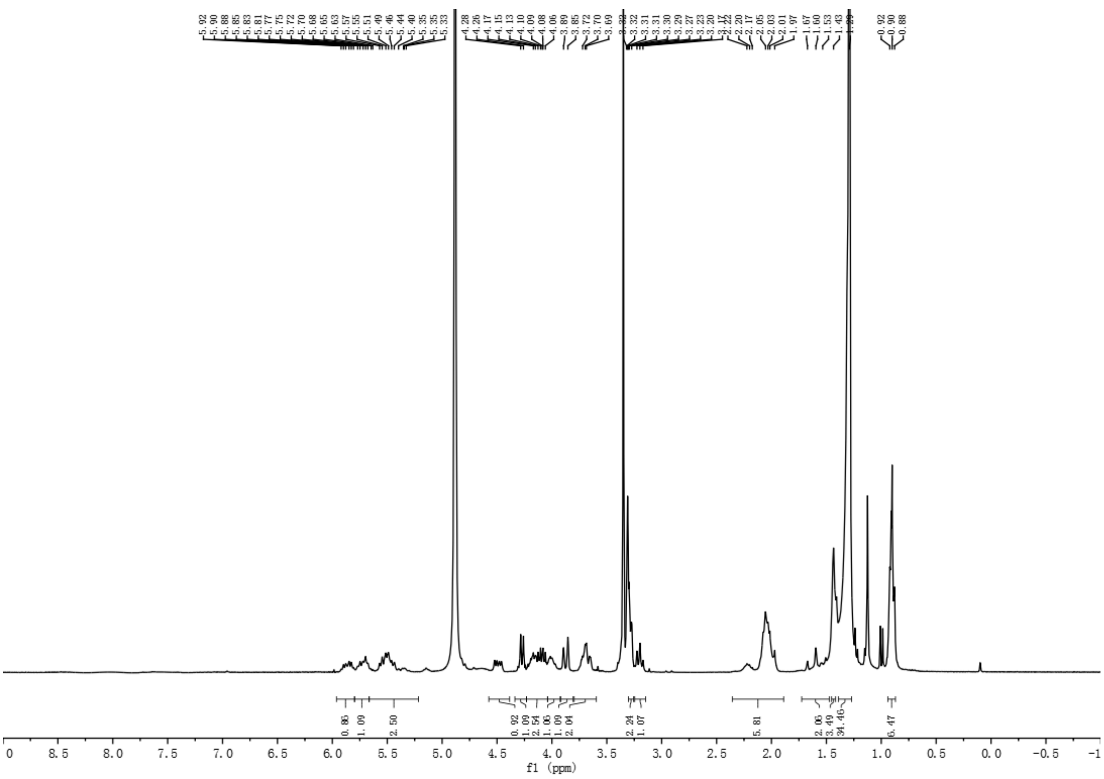

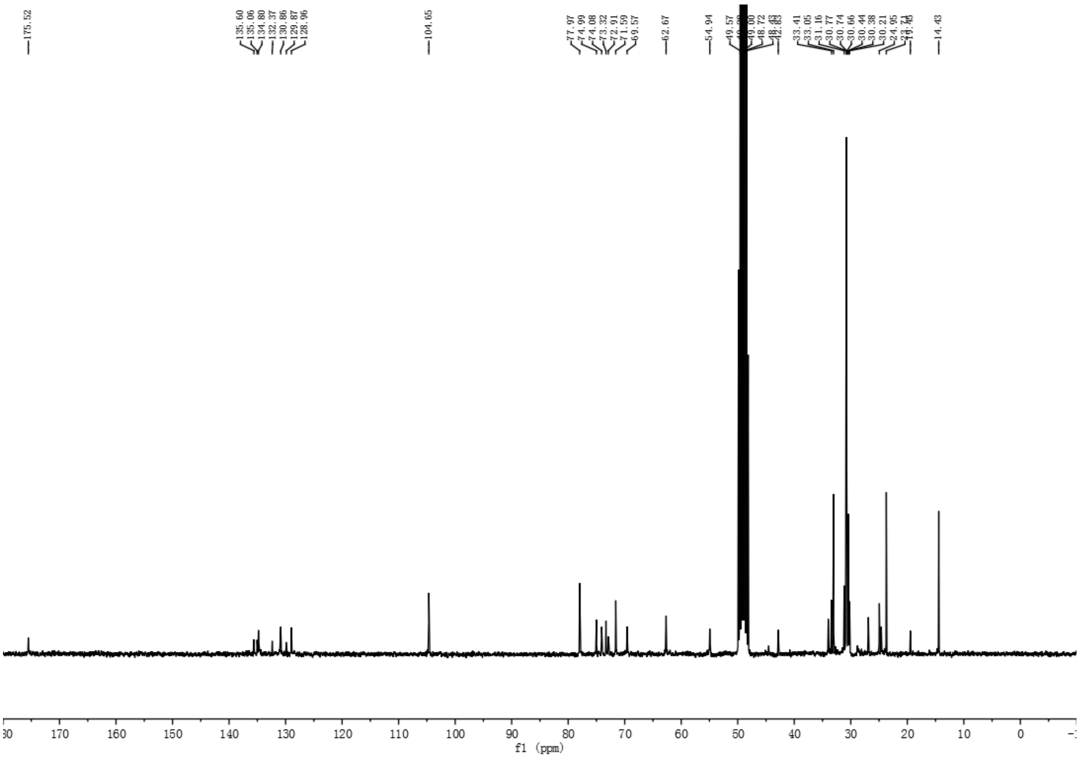

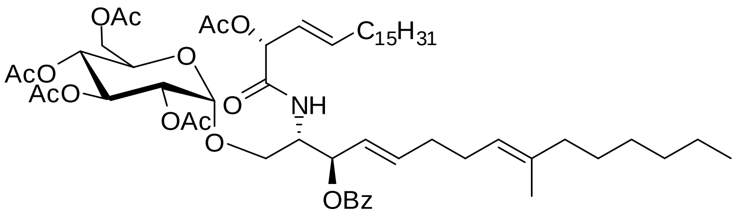

4

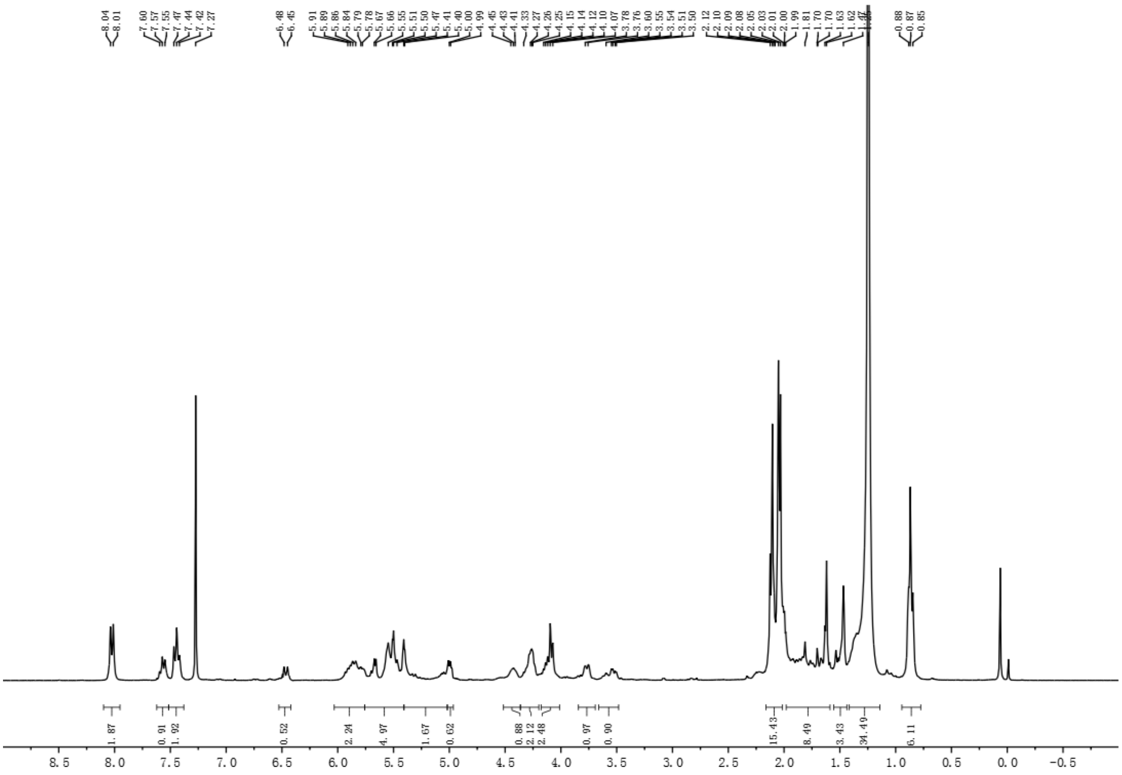

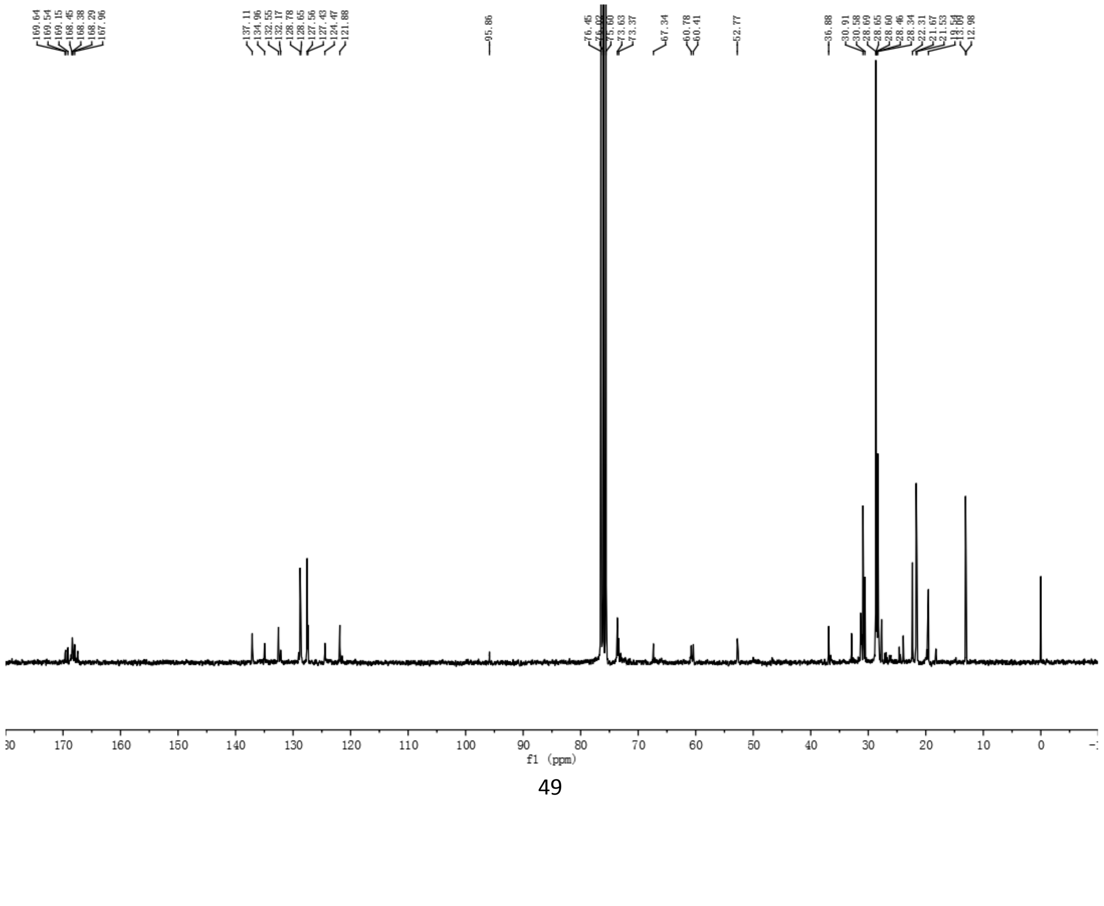

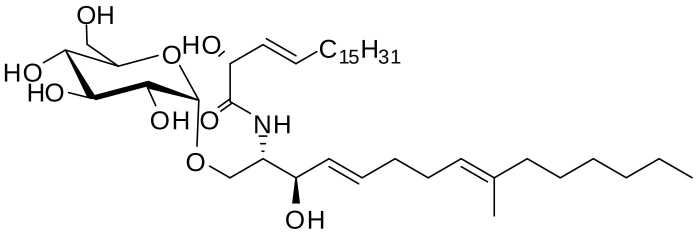

5

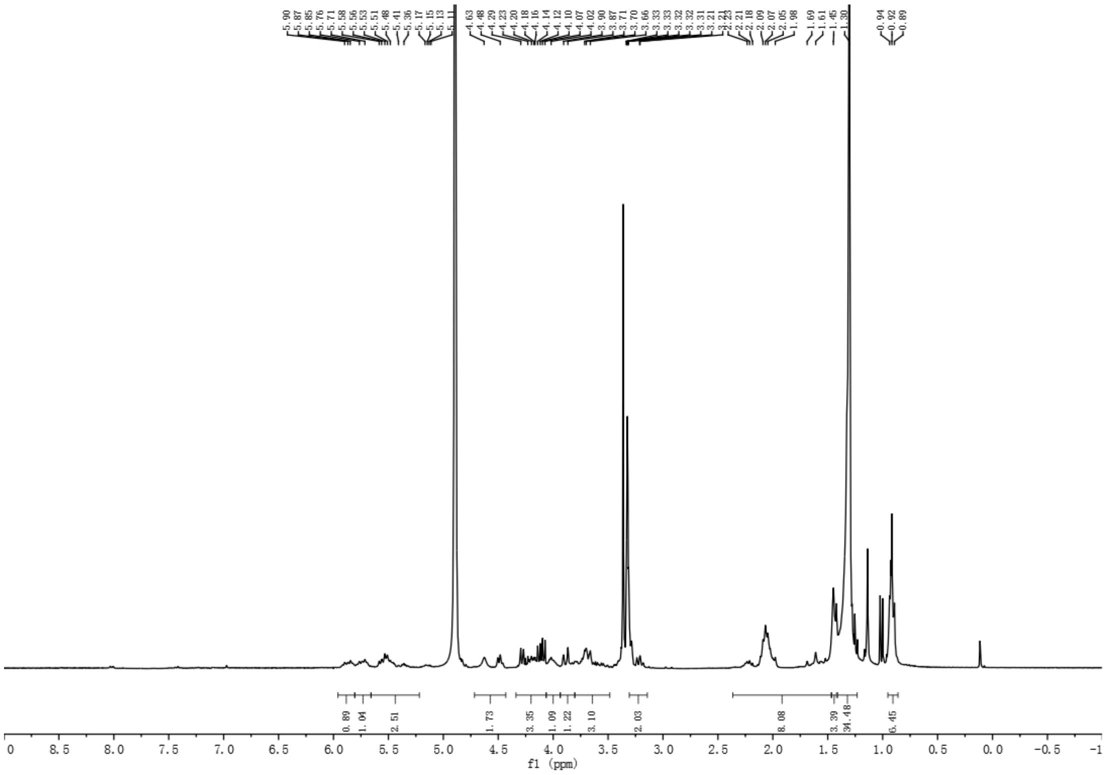

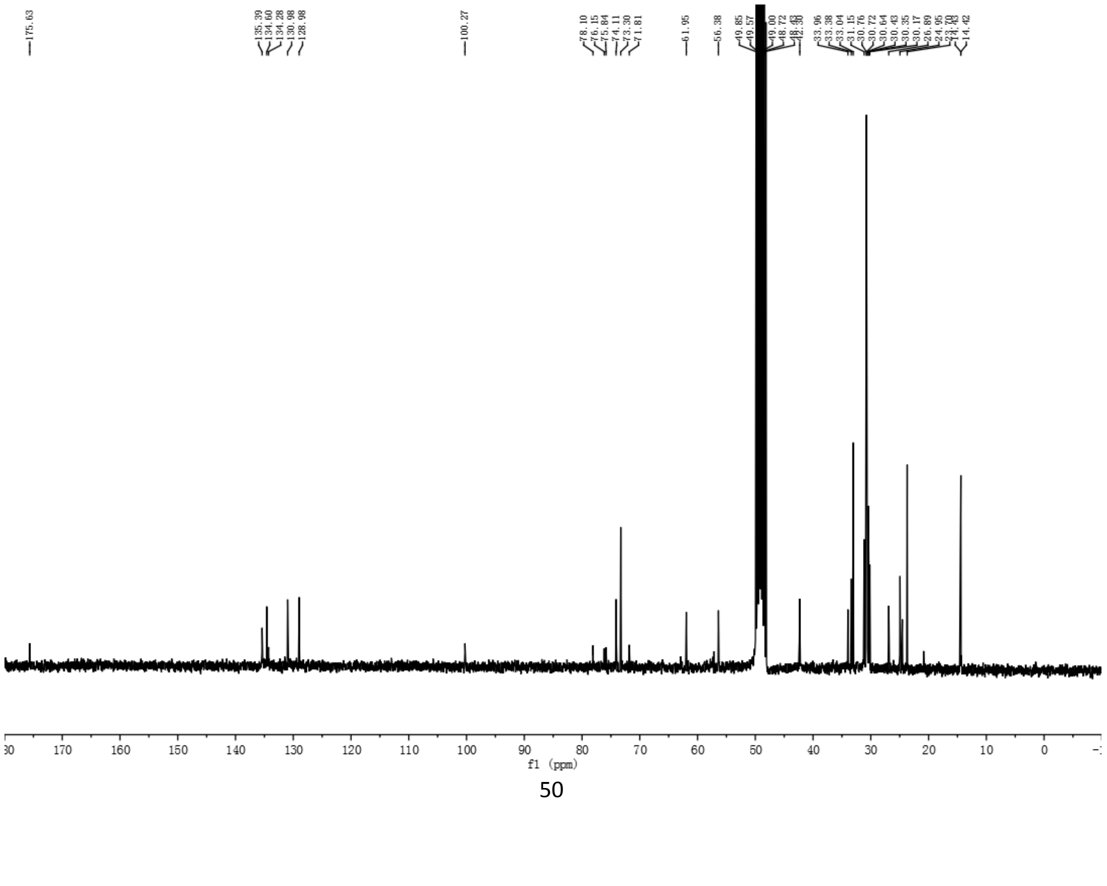

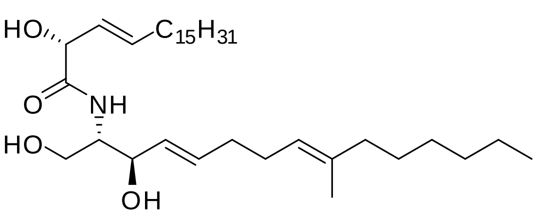

6

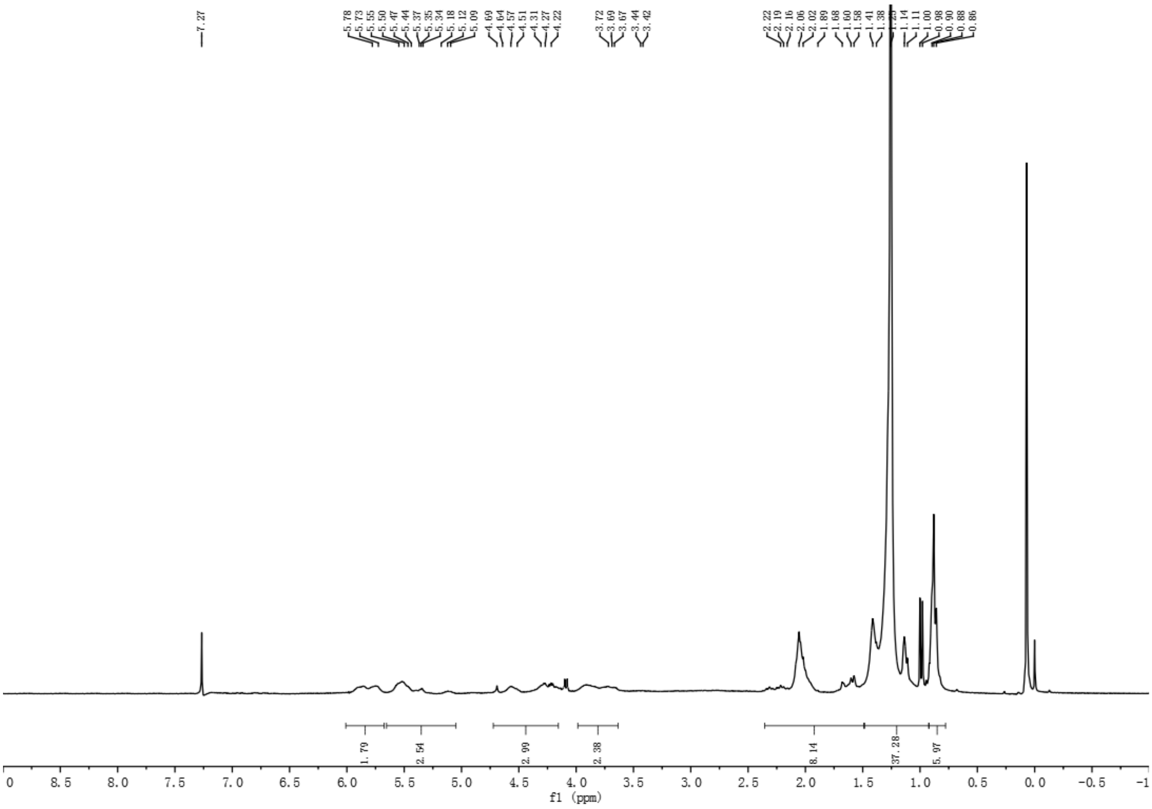

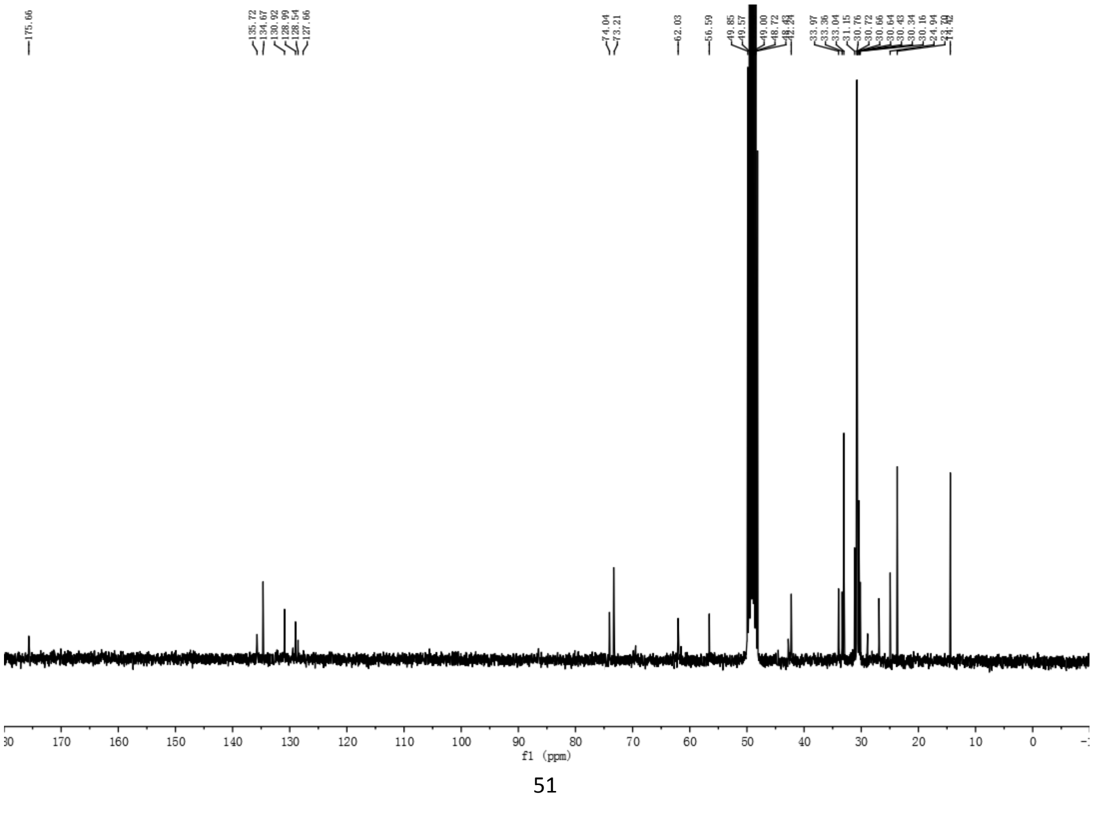

HRMS Date and MS Date of All Products

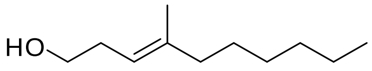

8

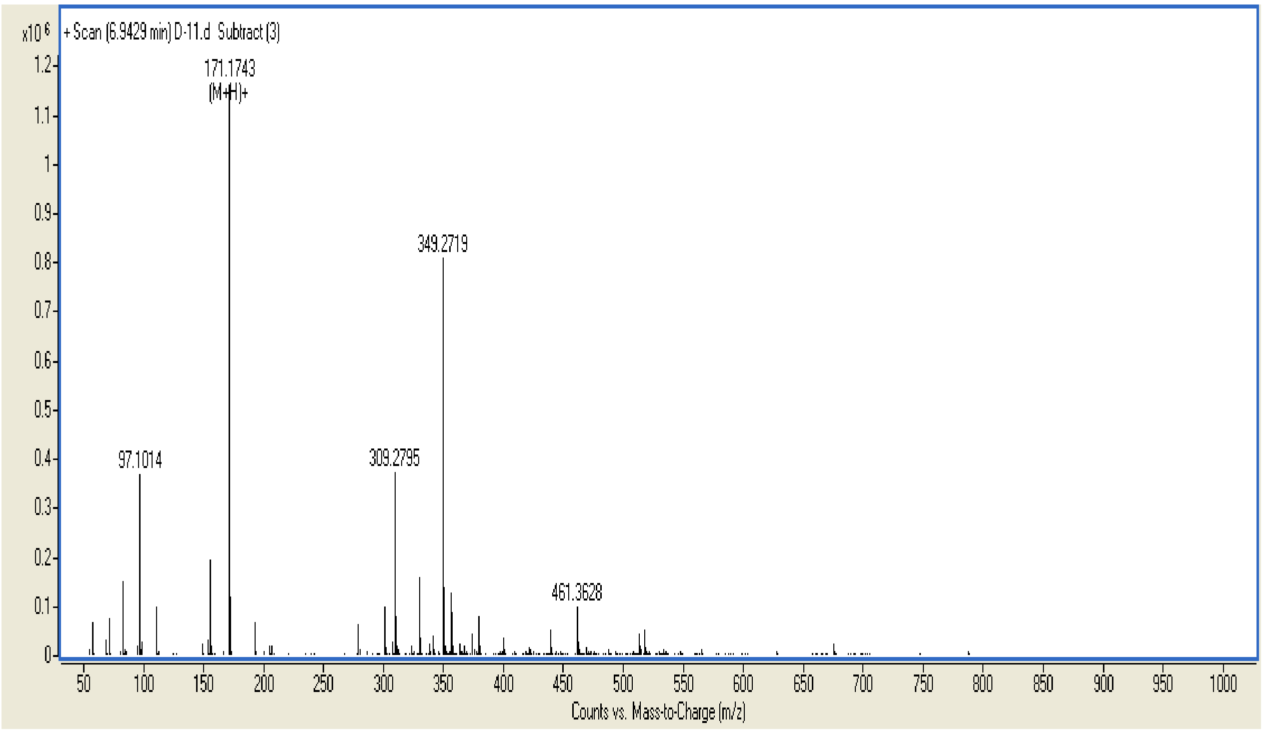

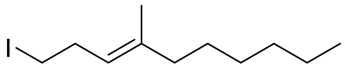

9

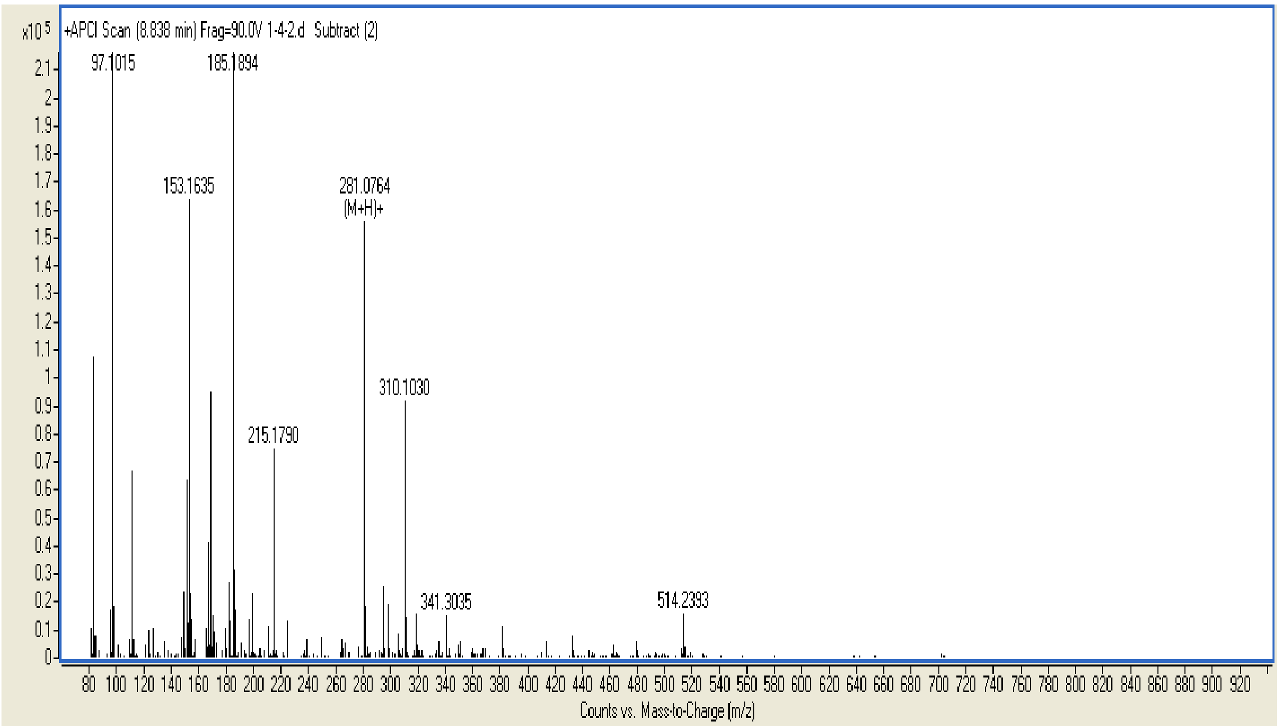

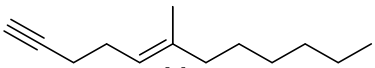

11

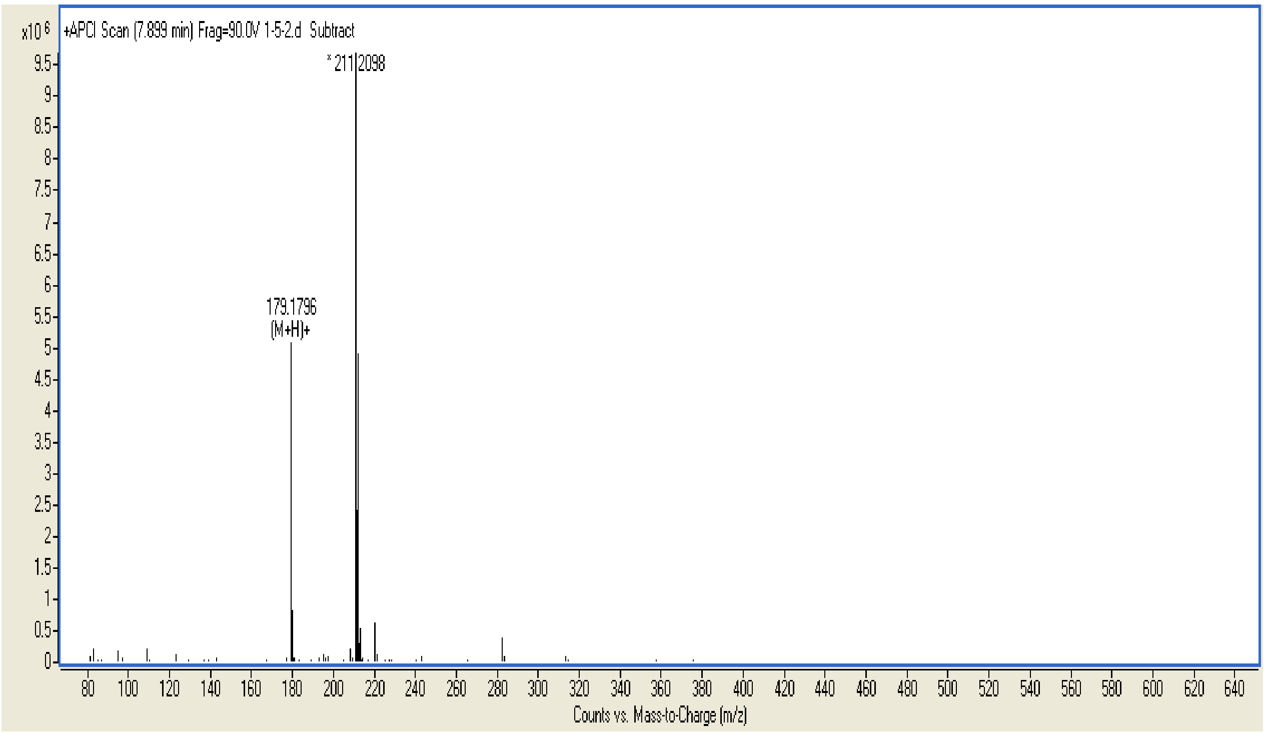
x10 6
+APCI Scan (7.899 min) Frag=90.0V 1-5-2.d Subtract
* 211.2098
179.1796
(M+H)+
Counts vs. Mass-to-Charge (m/z)

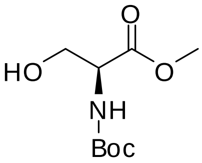

12

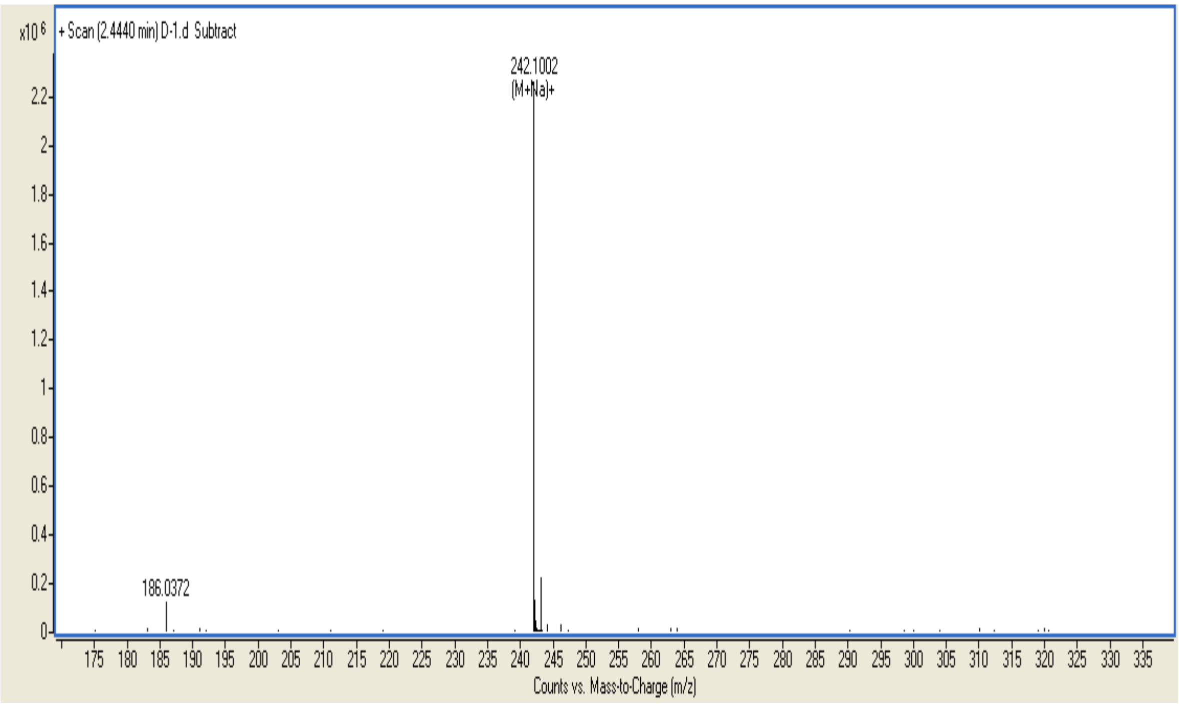
x10 6
+ Scan (2.4440 min) D-1.d Subtract
242.1002
(M+Na)+
186.0372
Counts vs. Mass-to-Charge (m/z)

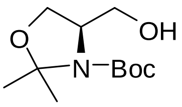
OH
O
N
Boc

14

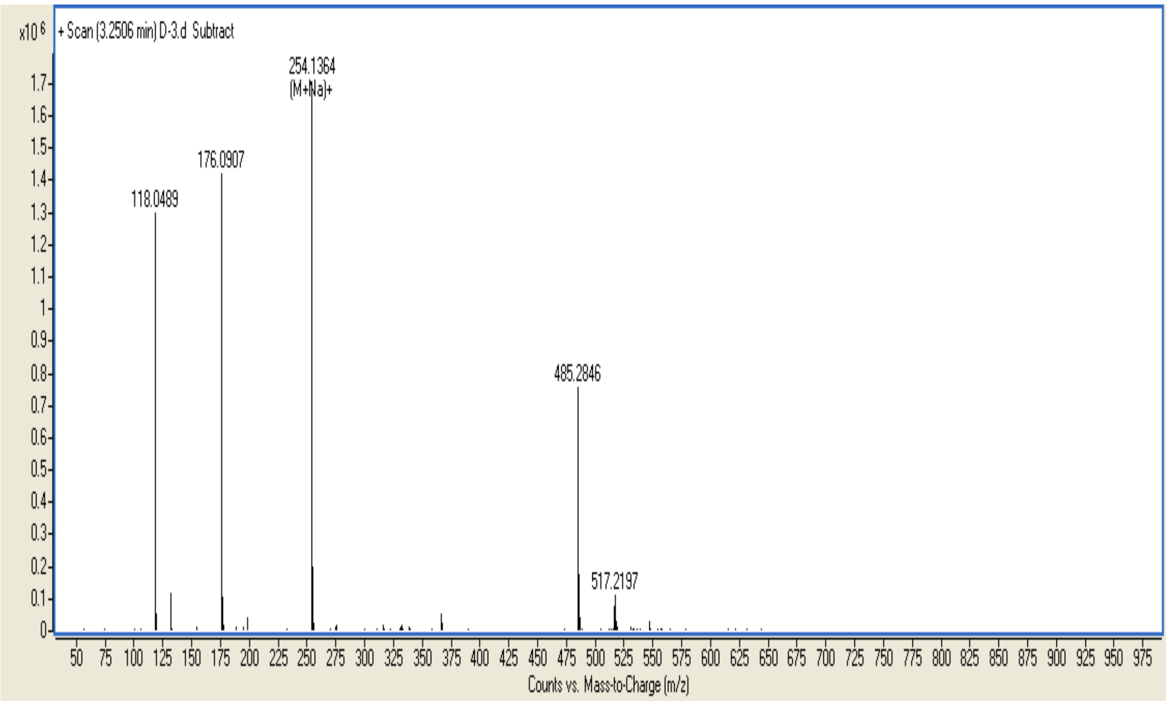
x10 6
+ Scan (3.2506 min) D-3.d Subtract
254.1364
(M+Na)+
176.0907
118.0489
485.2846
517.2197
Counts vs. Mass-to-Charge (m/z)

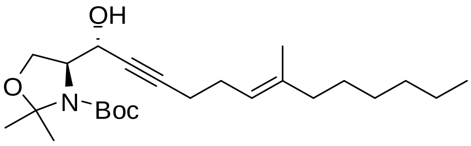
OH
O
N
Boc

16

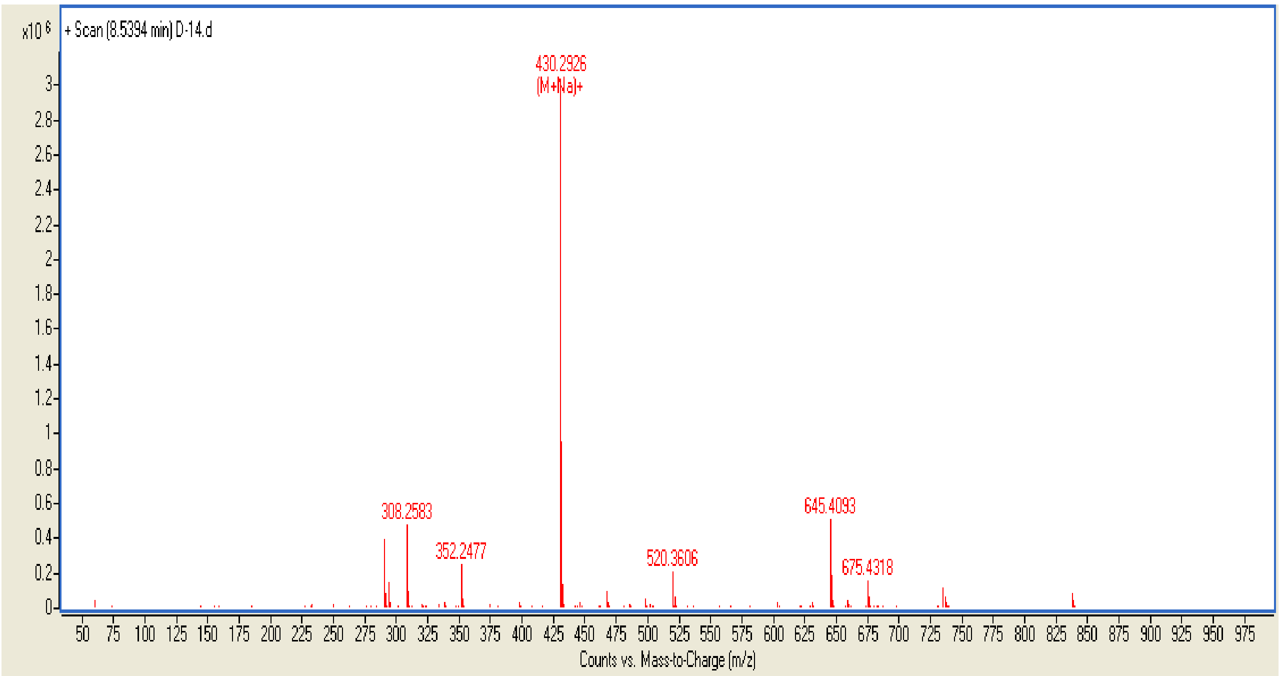
x10 6
+ Scan (8.5394 min) D-14.d
430.2926
(M+Na)+
308.2583
352.2477
520.3606
645.4093
675.4318
Counts vs. Mass-to-Charge (m/z)

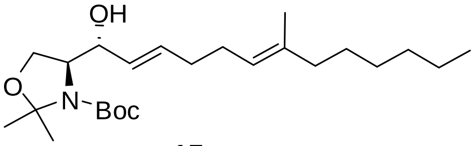
OH
O
N
Boc

17

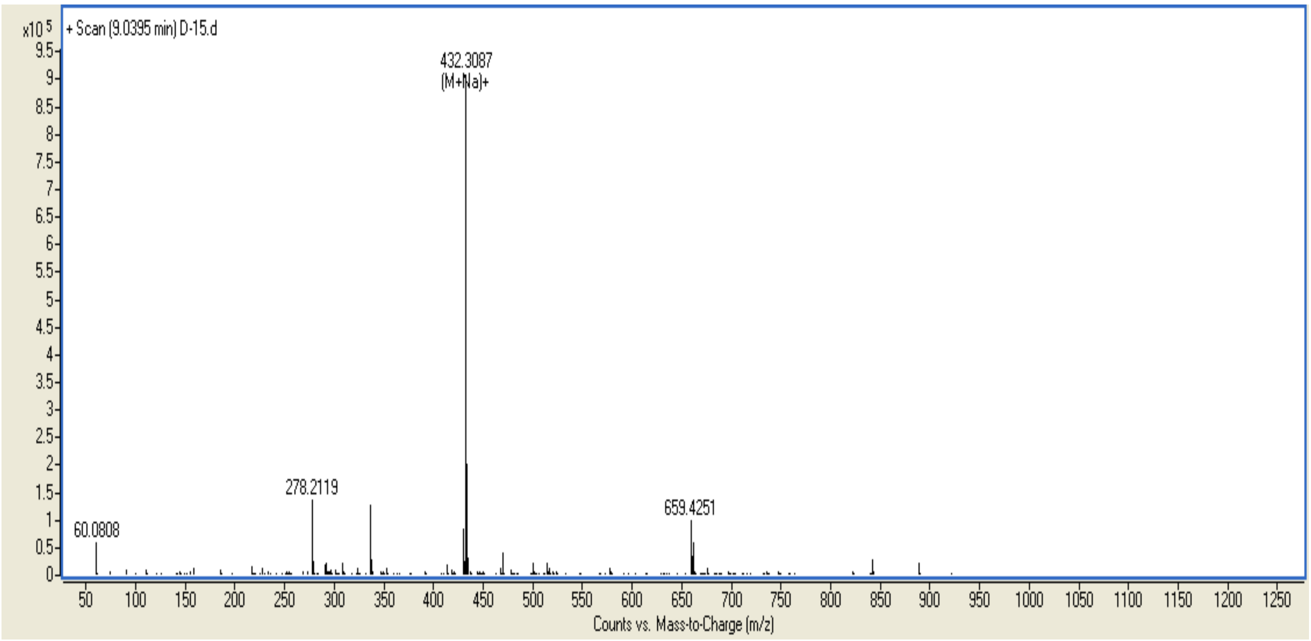
x10 5
+ Scan (9.0395 min) D-15.d
432.3087
(M+Na)+
278.2119
659.4251
60.0808
Counts vs. Mass-to-Charge (m/z)

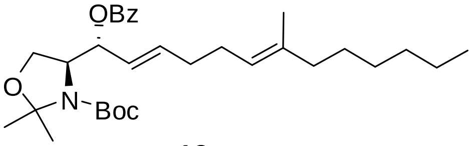
OBz
O
N
Boc

18

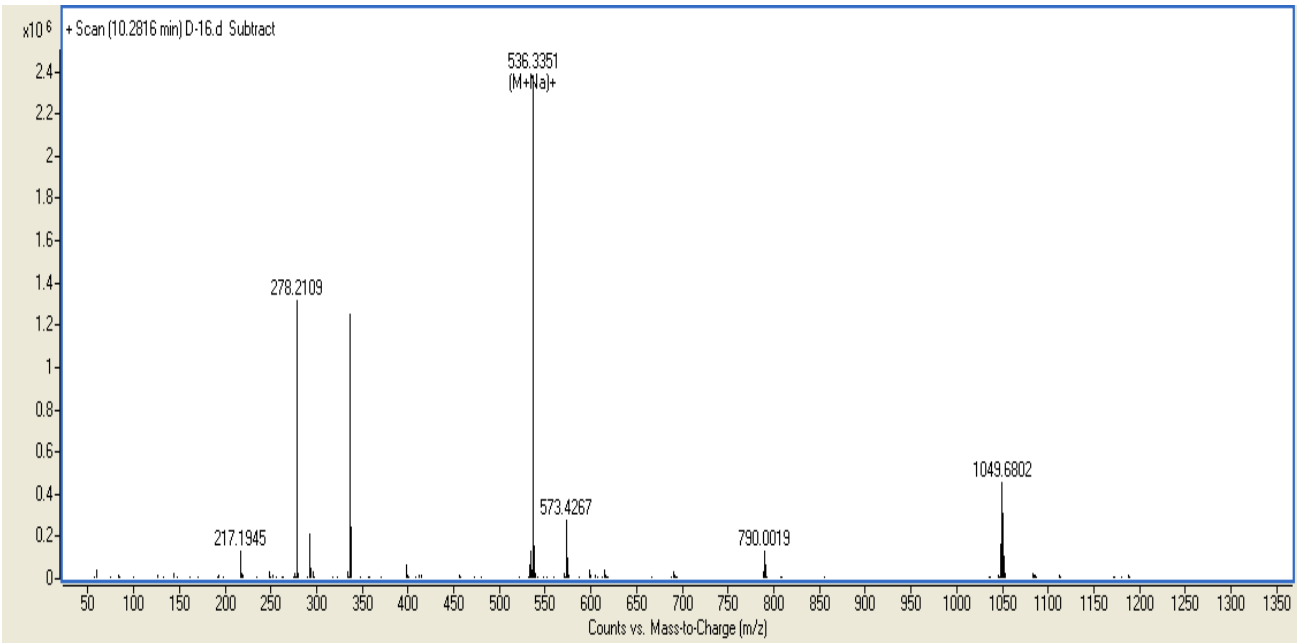
x10 6
+ Scan (10.2816 min) D-16.d Subtract
536.3351
(M+Na)+
278.2109
217.1945
573.4267
790.0019
1049.6802
Counts vs. Mass-to-Charge (m/z)

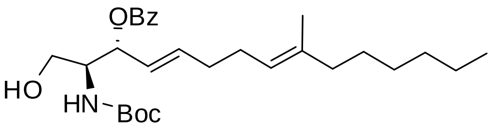
OBz
HO
HN
Boc

19

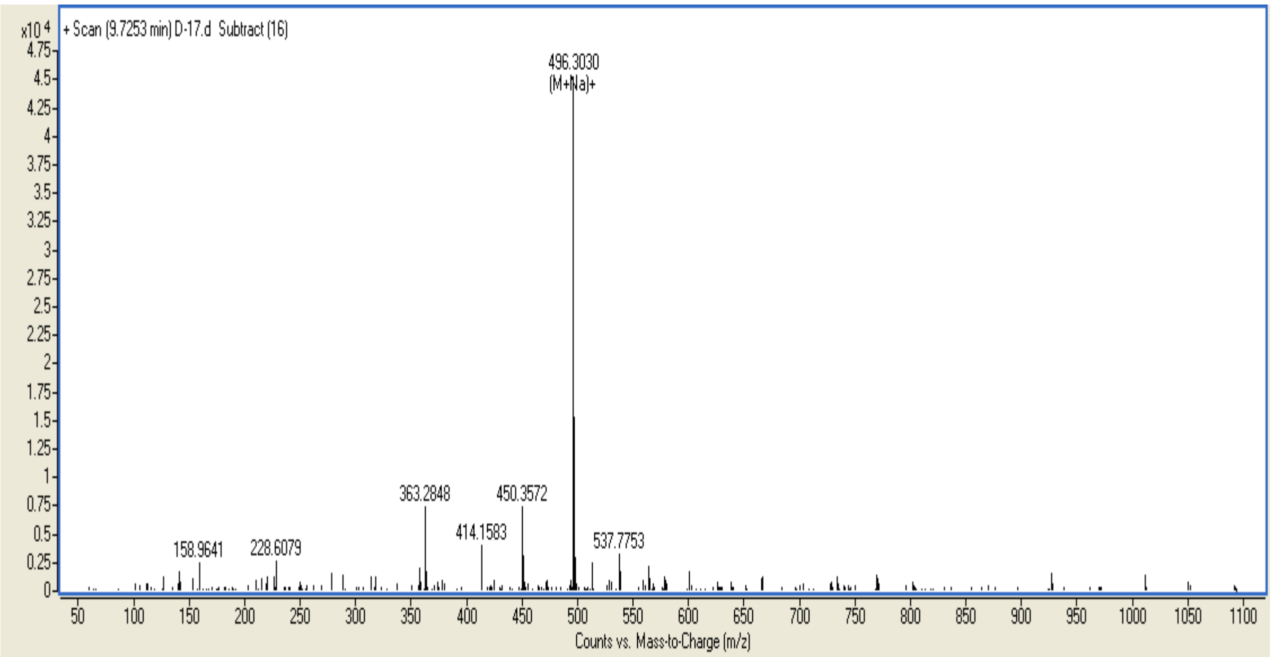
x10 4
+ Scan (9.7253 min) D-17.d Subtract (16)
496.3030
[M+Na]+
363.2848
450.3572
414.1583
537.7753
158.9641
228.6079
Counts vs. Mass-to-Charge (m/z)

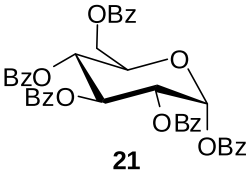
OBz
BzO
BzO
O
OBz
OBz

21

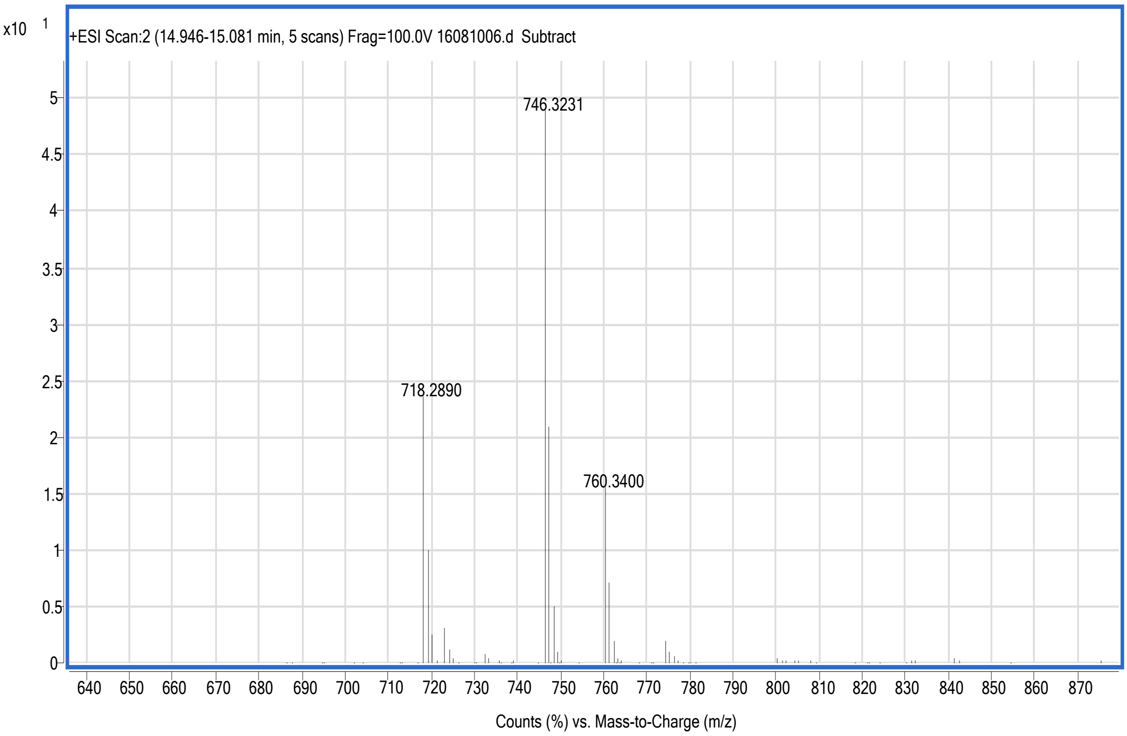
x10 1
+ESI Scan:2 (14.946-15.081 min, 5 scans) Frag=100.0V 16081006.d Subtract
746.3231
718.2890
760.3400
Counts (%) vs. Mass-to-Charge (m/z)

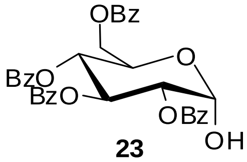
OBz
BzO
BzO
O
OBz
OH

23

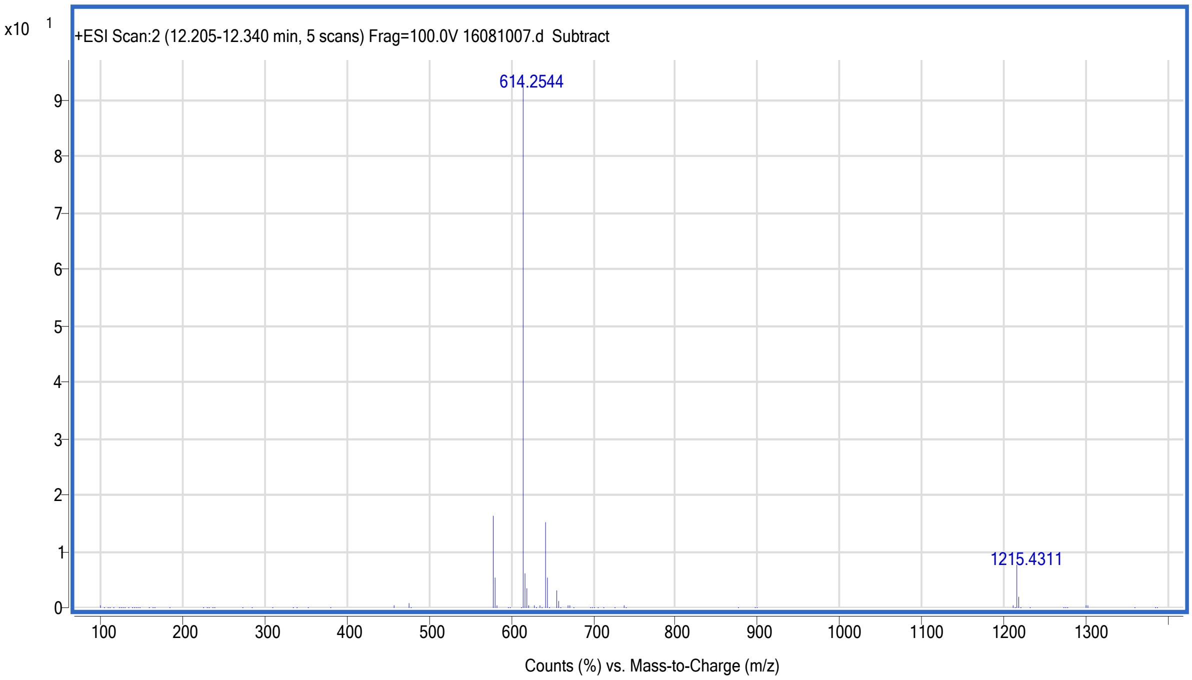
x10 1
+ESI Scan:2 (12.205-12.340 min, 5 scans) Frag=100.0V 16081007.d Subtract
614.2544
1215.4311
Counts (%) vs. Mass-to-Charge (m/z)

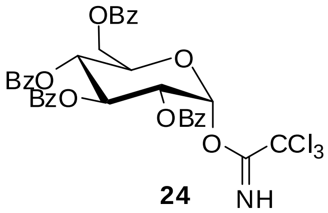
OBz
BzO
BzO
O
OBz
O
CCl3
NH

24

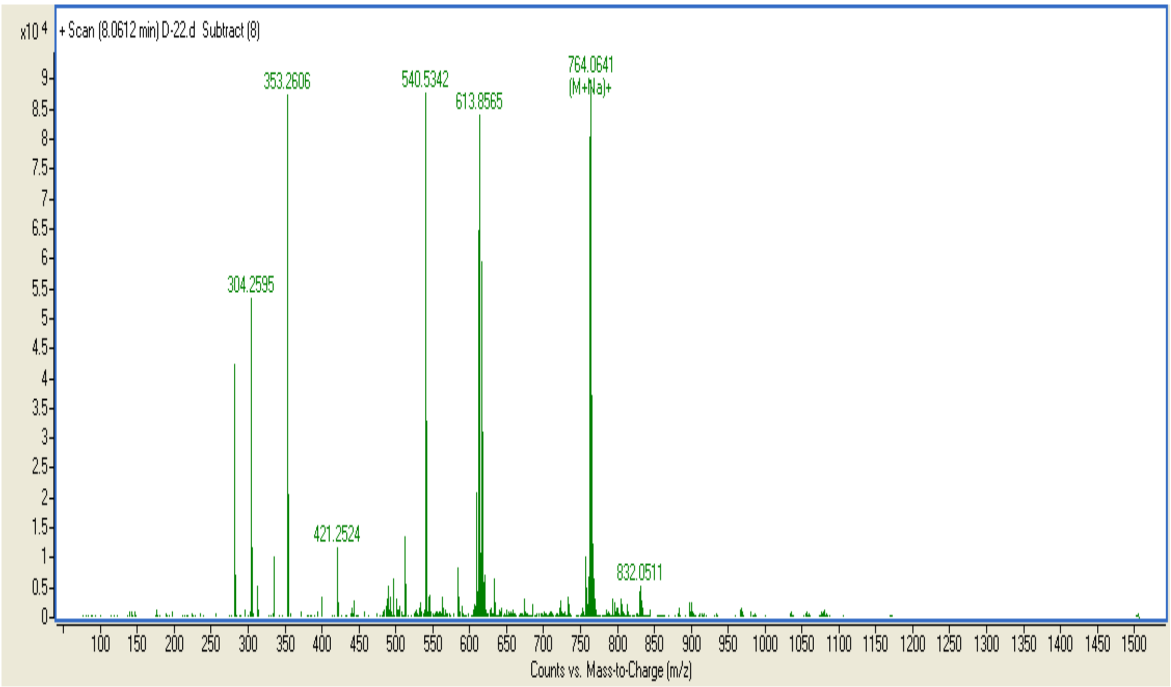
x10 4
+ Scan (8.0612 min) D-22.d Subtract (8)
353.2606
540.5342
613.8565
764.0641
(M+Na)+
304.2595
421.2524
832.0511
Counts vs. Mass-to-Charge (m/z)

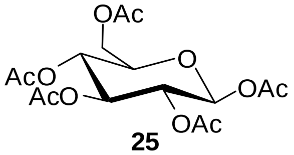
OAc
AcO
AcO
O
OAc
OAc

25

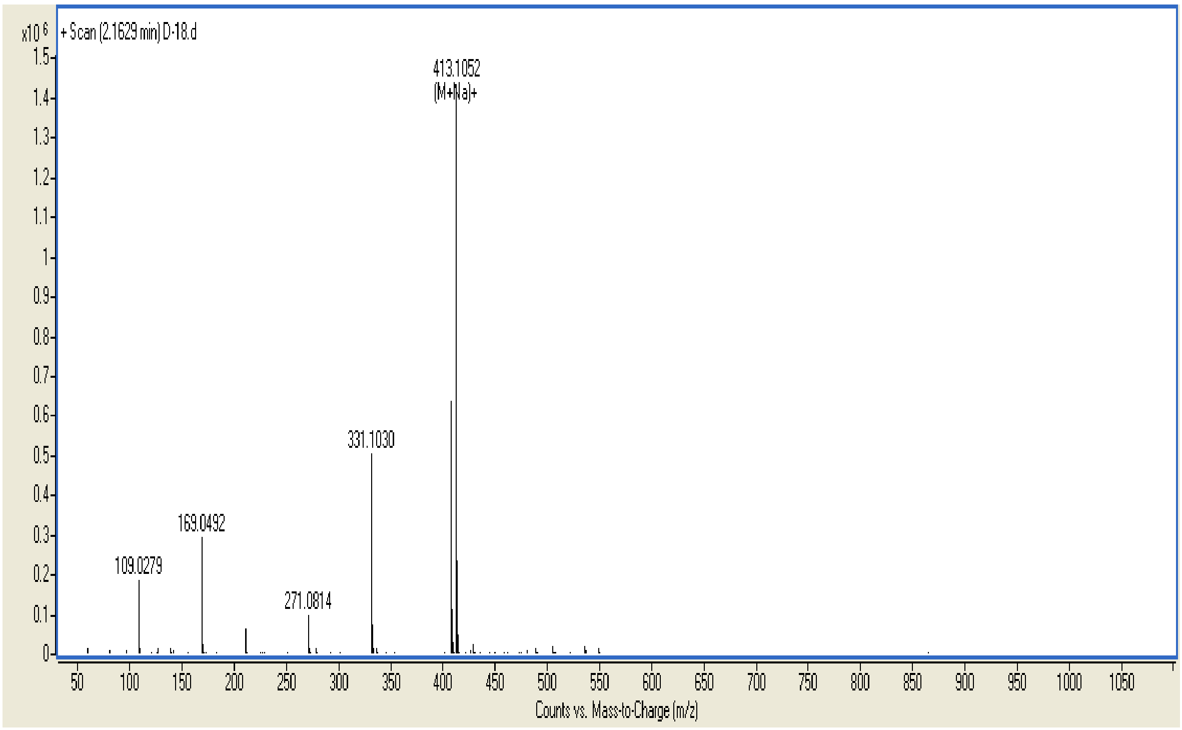
x10 6
+ Scan (2.1629 min) D-18.d
413.1052
(M+Na)+
331.1030
169.0492
109.0279
271.0814
Counts vs. Mass-to-Charge (m/z)

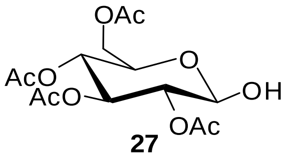
OAc
AcO
AcO
O
OH
OAc

27

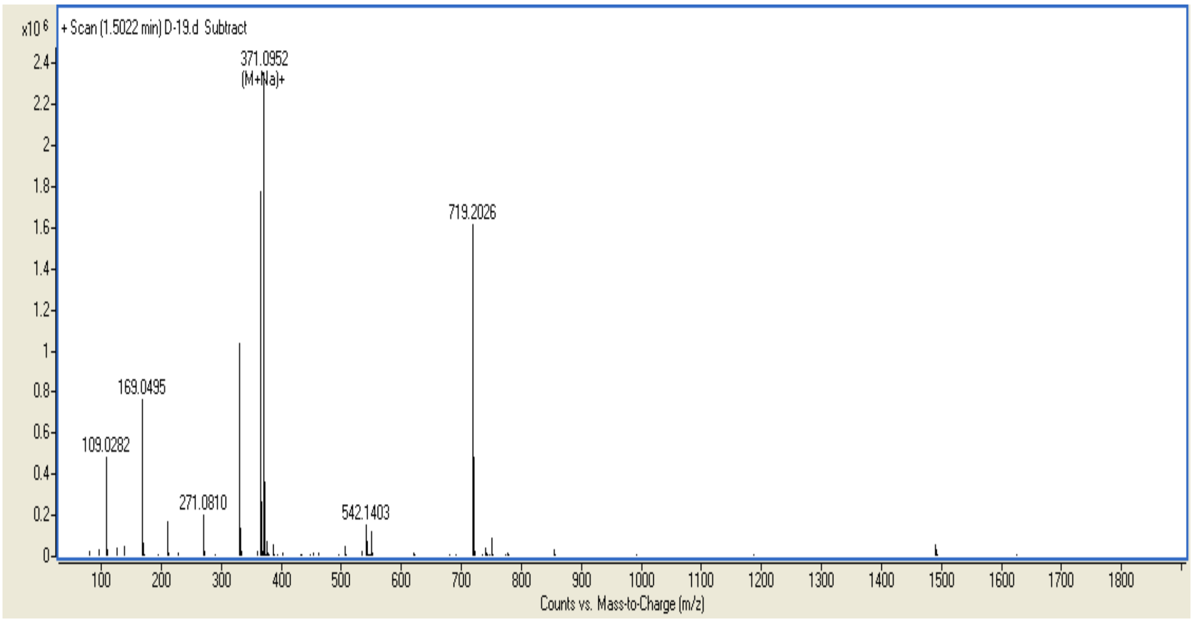
x10 6
+ Scan (1.5022 min) D-19.d Subtract
371.0952
(M+Na)+
719.2026
169.0495
109.0282
271.0810
542.1403
Counts vs. Mass-to-Charge (m/z)

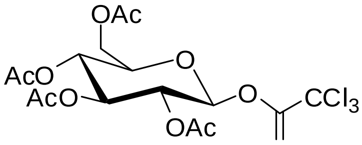

28

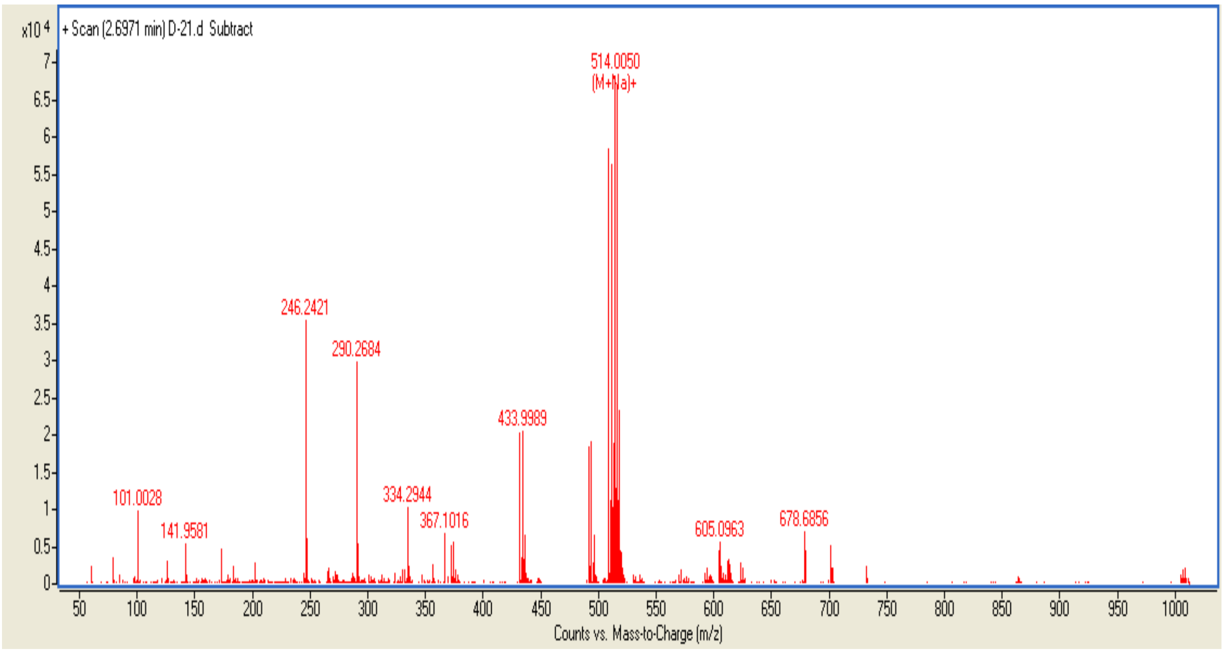

I—$C_{15}H_{31}$

29

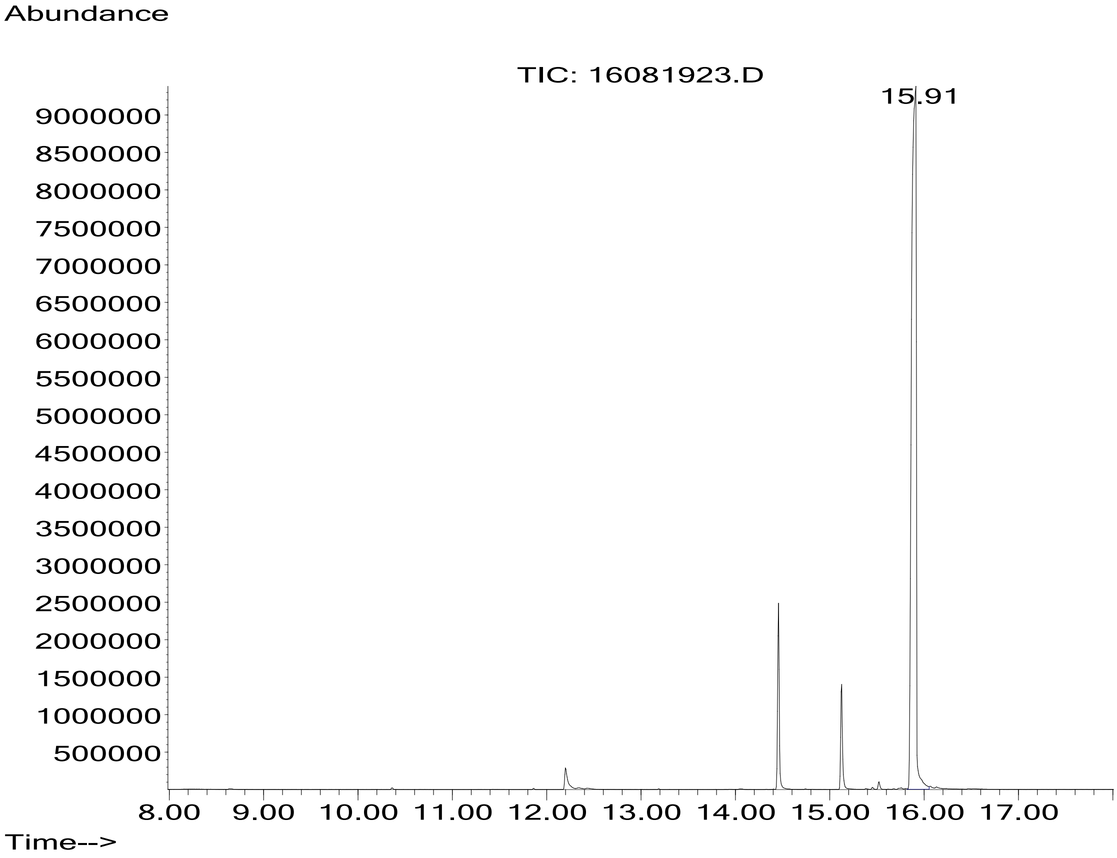

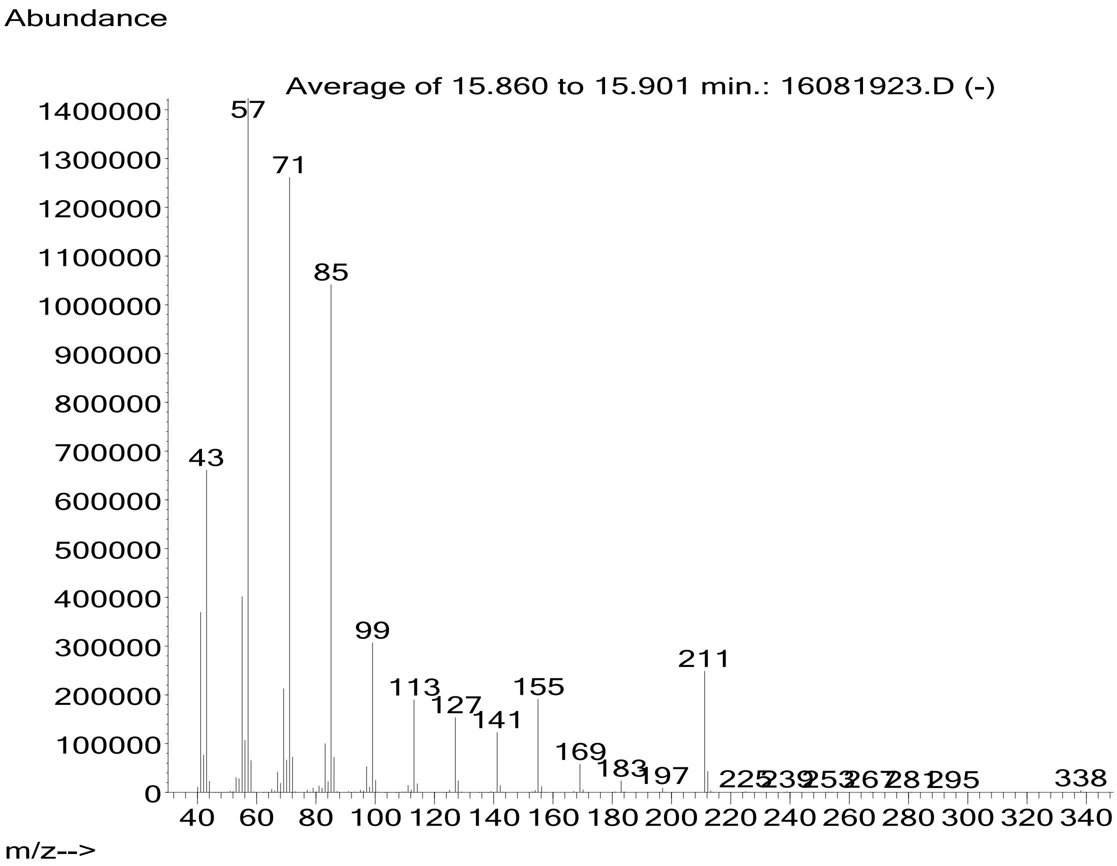
Abundance
Average of 15.860 to 15.901 min.: 16081923.D (-)
1400000
1300000
1200000
1100000
1000000
900000
800000
700000
600000
500000
400000
300000
200000
100000
0
57
71
85
43
99
113
127
141
155
169
183
197
211
225
239
253
267
281
295
338
40 60 80 100 120 140 160 180 200 220 240 260 280 300 320 340
m/z-->

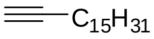
$C_{15}H_{31}$

30

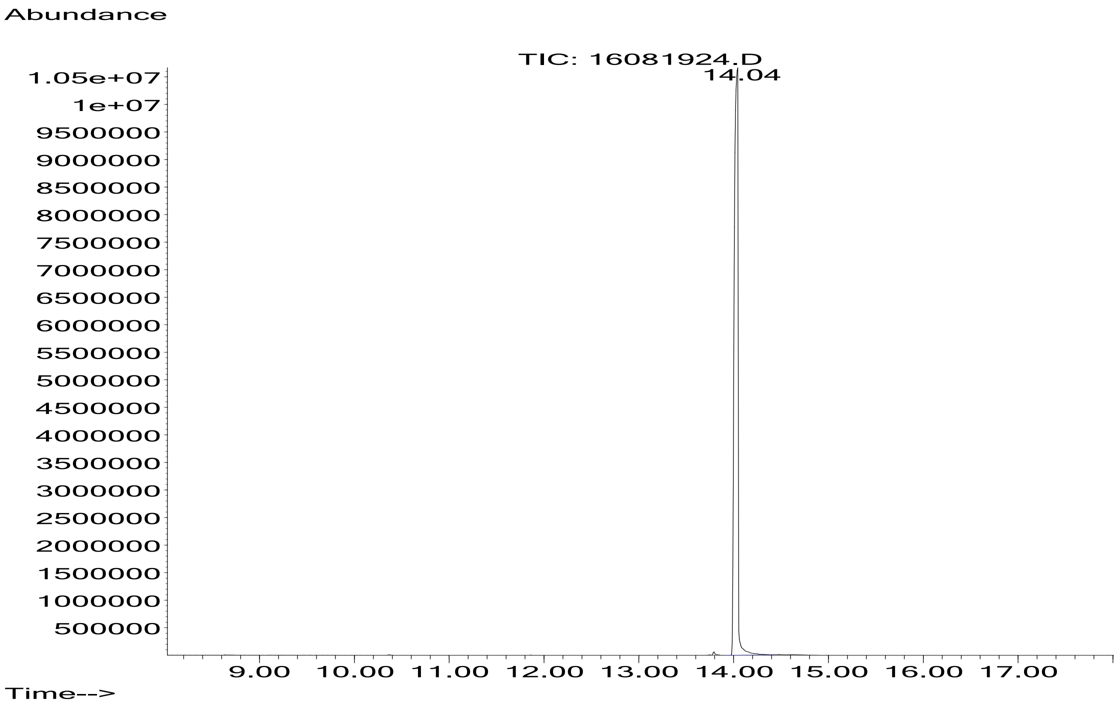
Abundance
TIC: 16081924.D
14.04
1.05e+07
1e+07
9500000
9000000
8500000
8000000
7500000
7000000
6500000
6000000
5500000
5000000
4500000
4000000
3500000
3000000
2500000
2000000
1500000
1000000
500000
9.00 10.00 11.00 12.00 13.00 14.00 15.00 16.00 17.00
Time-->

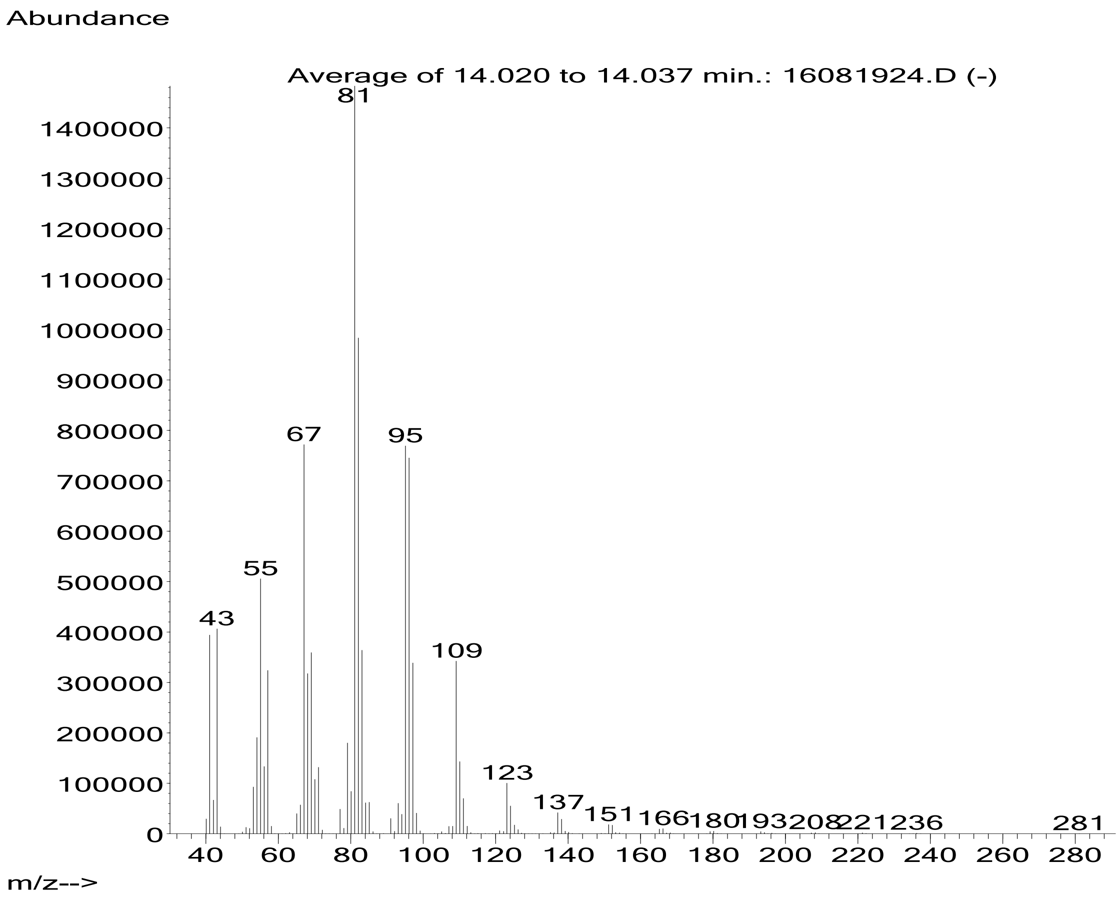
Abundance
Average of 14.020 to 14.037 min.: 16081924.D (-)
81
67
95
55
43
109
123
137
151
166
180
193
208
221
236
281
m/z-->

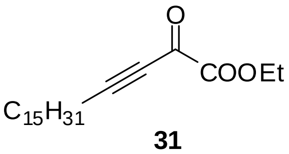
O
COOEt
$C_{15}H_{31}$

31

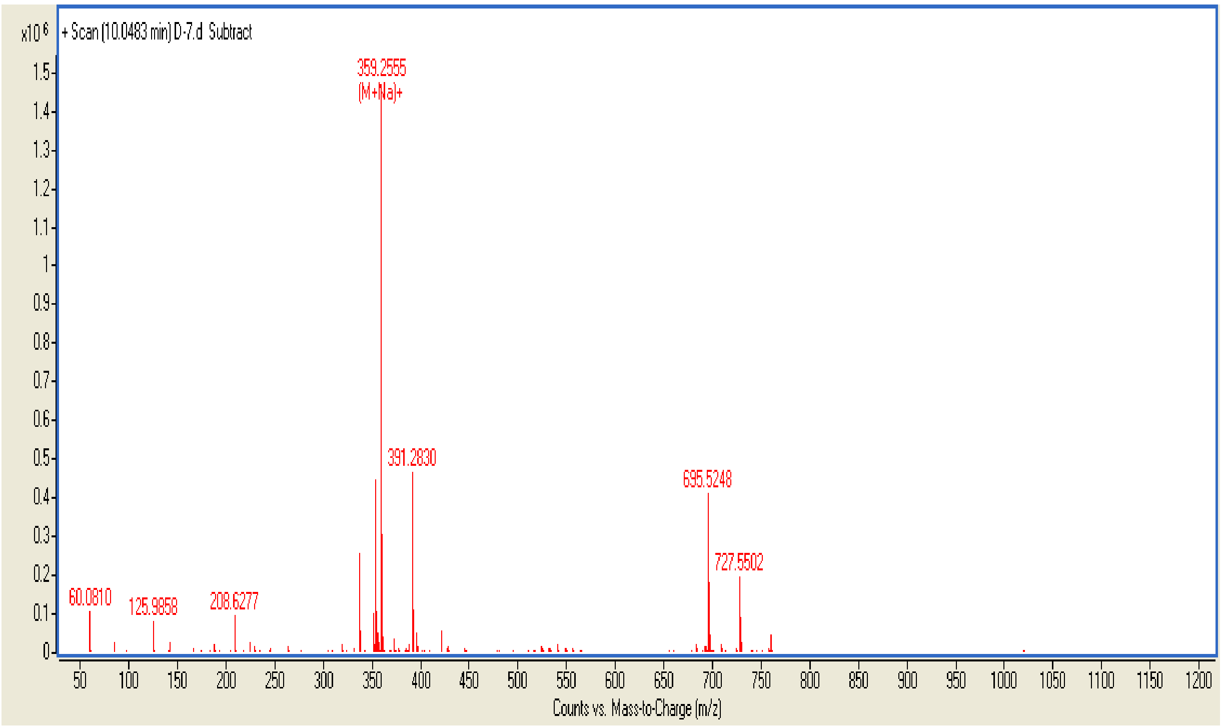
x10 6
+ Scan (10.0483 min) D-7.d Subtract
359.2555
(M+Na)+
391.2830
695.5248
727.5502
60.0810
125.9858
208.6277
Counts vs. Mass-to-Charge (m/z)

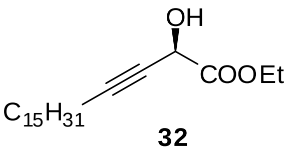
OH
COOEt
C15H31

32

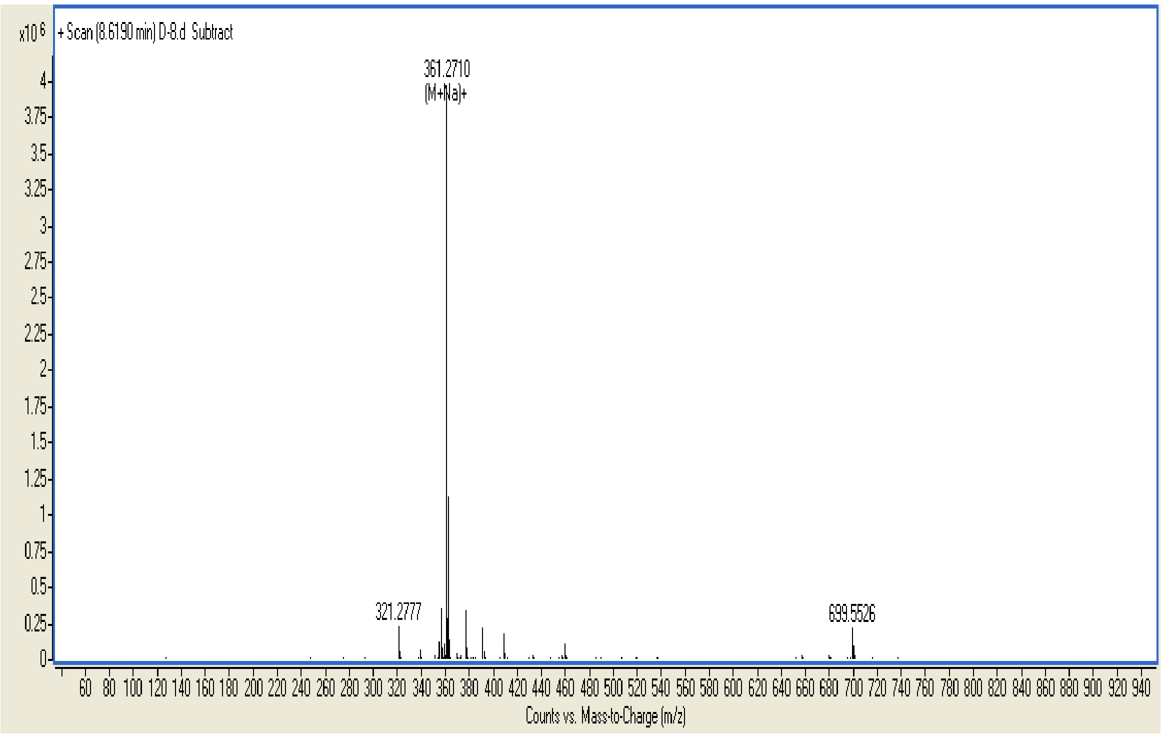
x10 6
+ Scan (8.6190 min) D-8.d Subtract
361.2710
(M+Na)+
321.2777
699.5526
Counts vs. Mass-to-Charge (m/z)

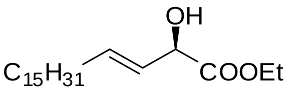
OH
C15H31
COOEt

33

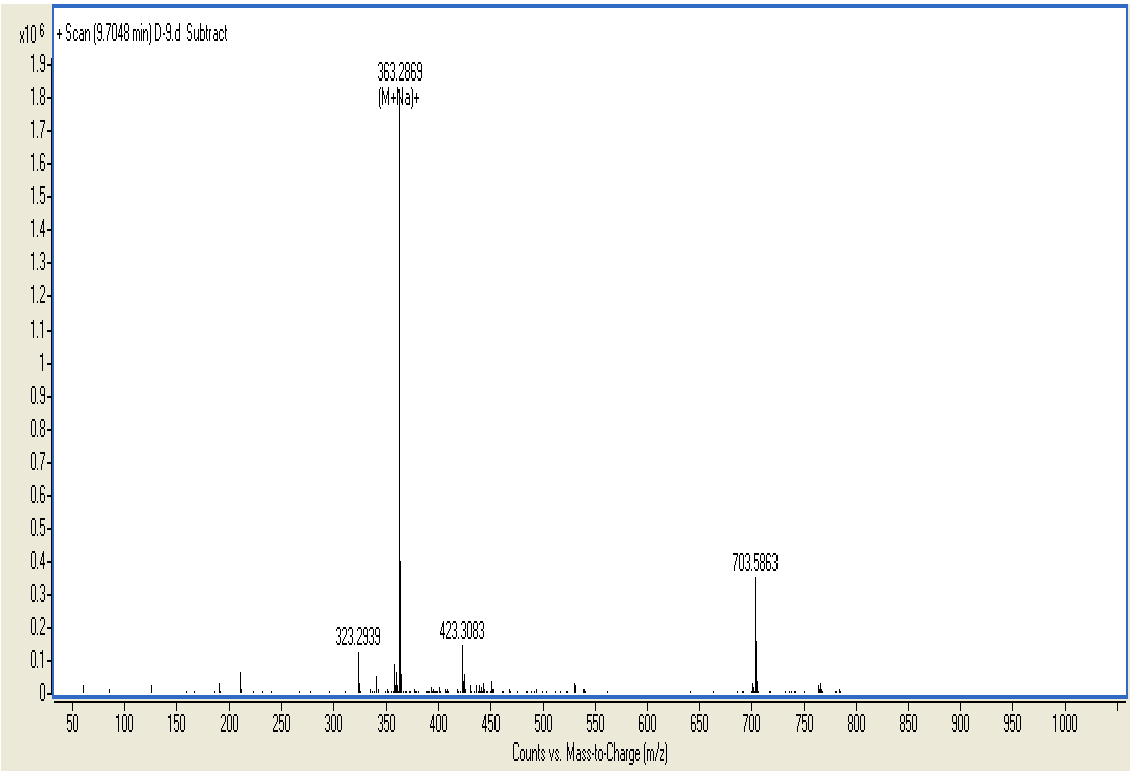
x10 6
+ Scan (9.7048 min) D-9.d Subtract
363.2869
(M+Na)+
323.2939
423.3083
703.5863
Counts vs. Mass-to-Charge (m/z)

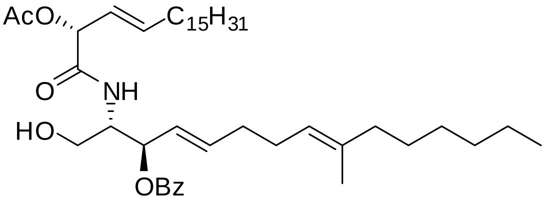
AcO
C15H31
O
NH
HO
OBz

1

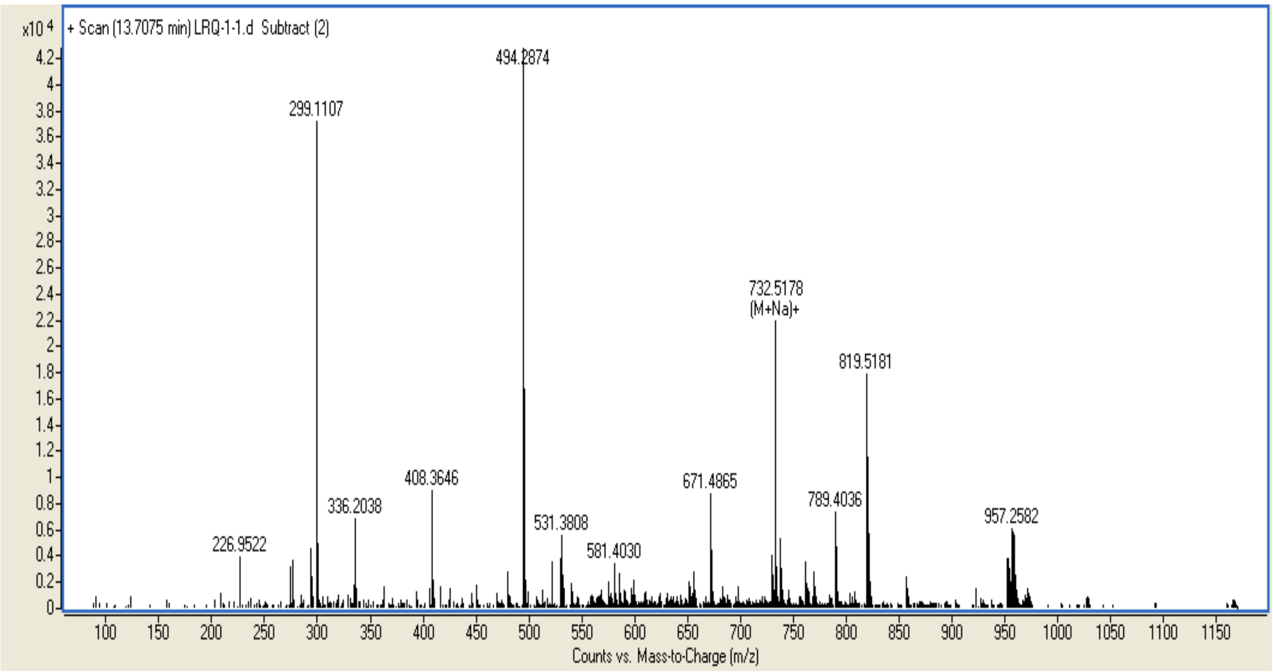
x10 4
+ Scan (13.7075 min) LRQ-1-1.d Subtract (2)
494.2874
299.1107
732.5178
(M+Na)+
819.5181
408.3646
671.4865
789.4036
336.2038
957.2582
531.3808
226.9522
581.4030
Counts vs. Mass-to-Charge (m/z)

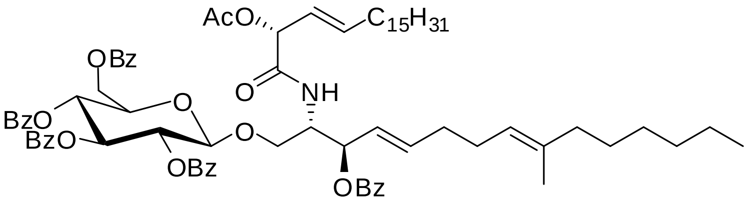
AcO
C15H31
OBz
O
NH
BzO
BzO
O
OBz
OBz

2

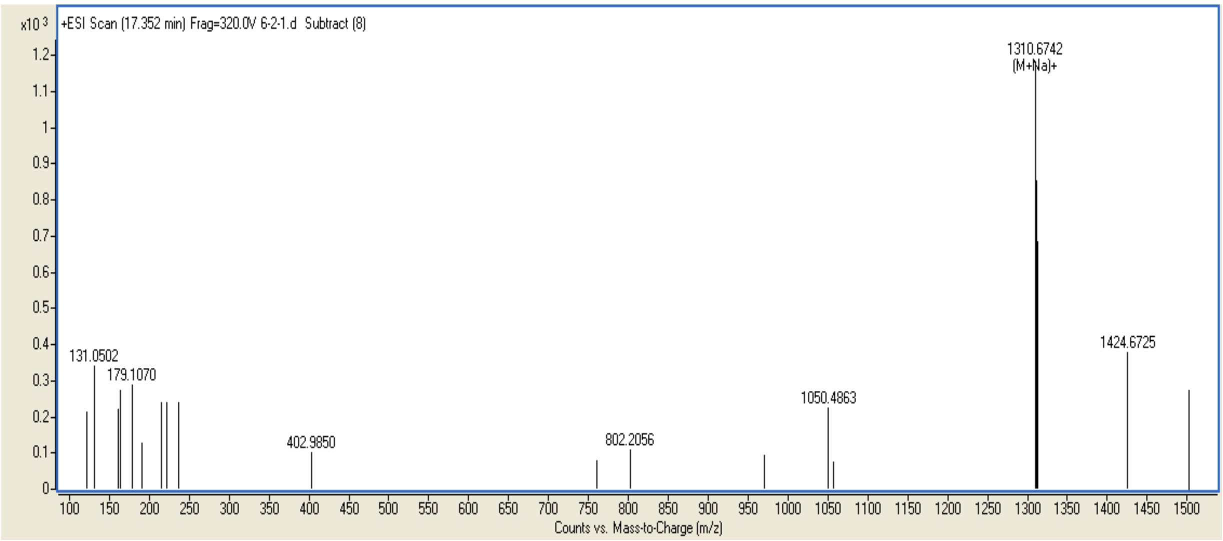
x10 3
+ESI Scan (17.352 min) Frag=320.0V 6-2-1.d Subtract (8)
1310.6742
(M+Na)+
1424.6725
131.0502
179.1070
1050.4863
402.9850
802.2056
Counts vs. Mass-to-Charge (m/z)

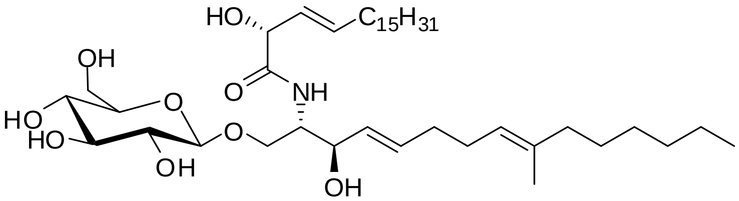
HO
C15H31
OH
O
NH
HO
HO
O
OH
OH

3

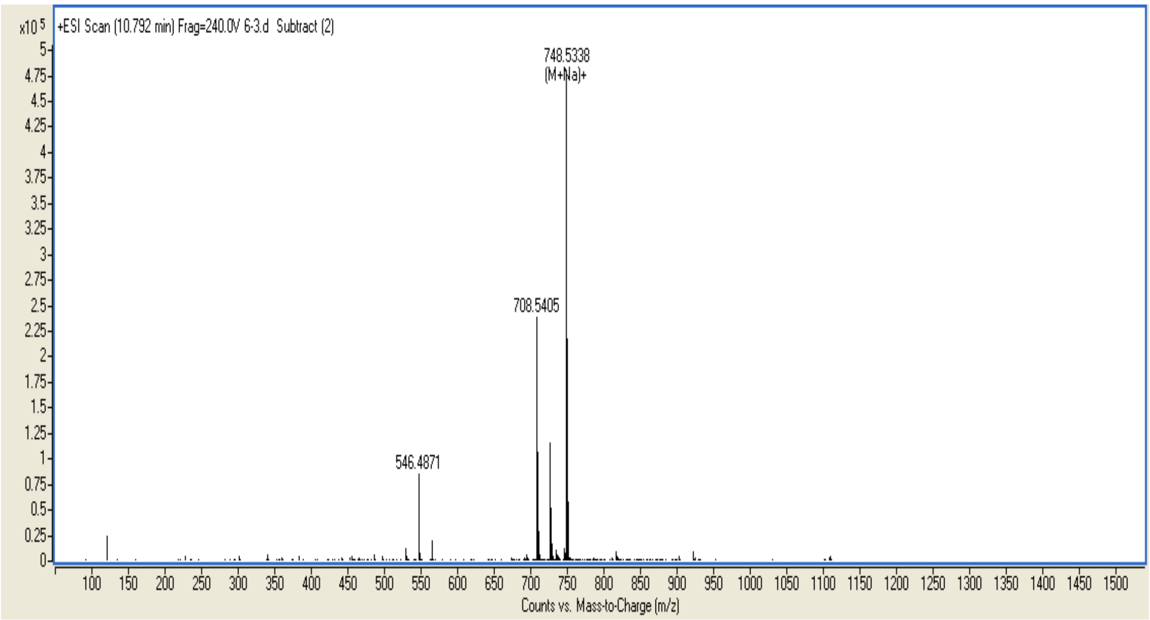
x10 5
+ESI Scan (10.792 min) Frag=240.0V 6-3.d Subtract (2)
748.5338
(M+Na)+
708.5405
546.4871
Counts vs. Mass-to-Charge (m/z)

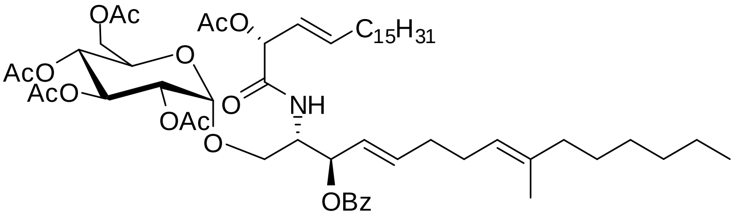
OAc
AcO
C15H31
AcO
AcO
O
NH
OAc
O
OBz

4

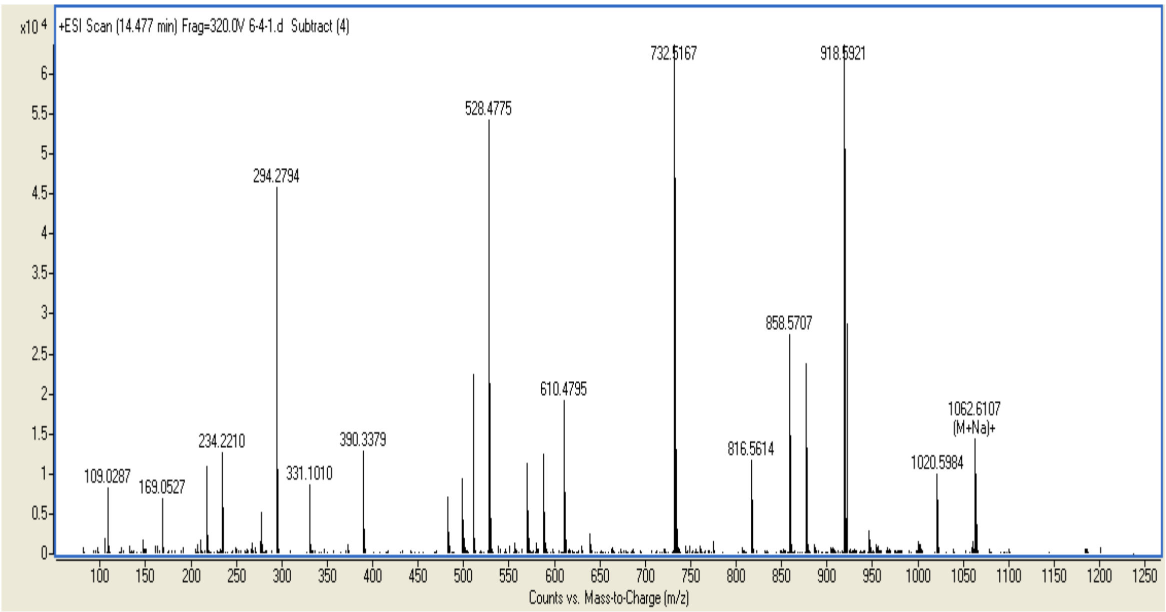
x10 4
+ESI Scan (14.477 min) Frag=320.0V 6-4-1.d Subtract (4)
732.5167
918.5921
528.4775
294.2794
858.5707
610.4795
1062.6107
(M+Na)+
234.2210
390.3379
816.5614
1020.5984
109.0287
169.0527
331.1010
Counts vs. Mass-to-Charge (m/z)

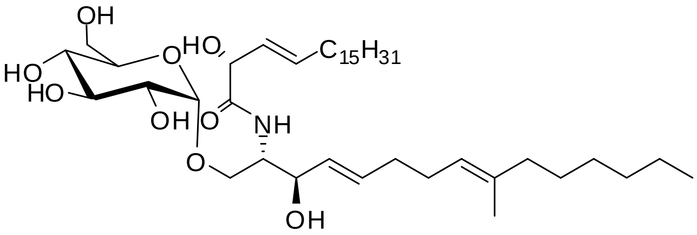
OH
HO
HO
O
HO
C15H31
OH
O
NH
O
OH

5

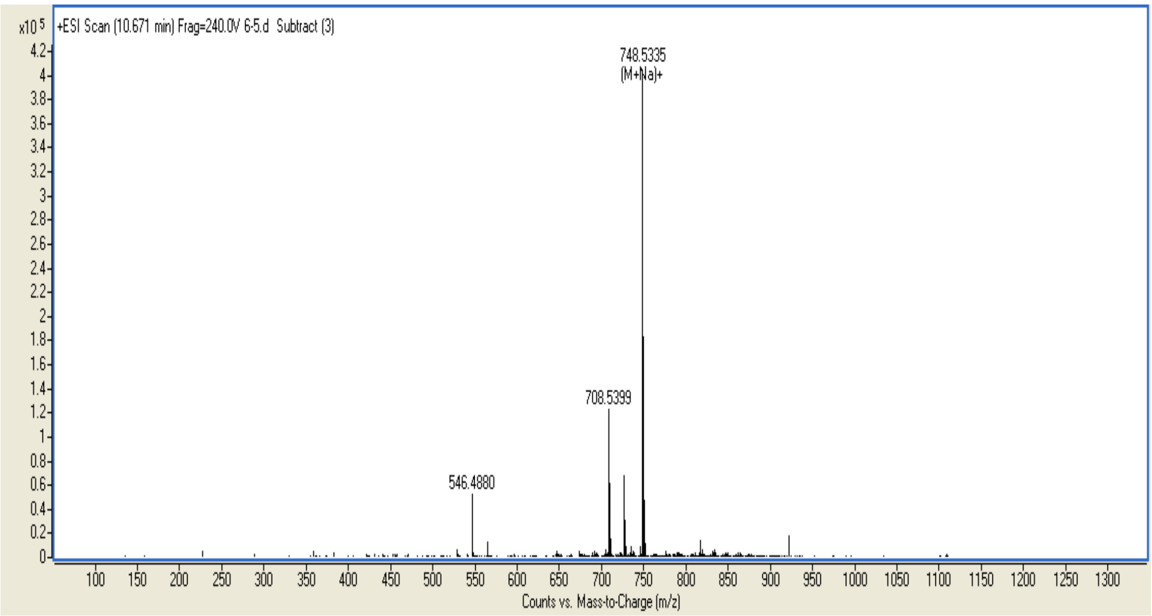
x10 5
+ESI Scan (10.671 min) Frag=240.0V 6-5.d Subtract (3)
748.5335
(M+Na)+
708.5399
546.4880
Counts vs. Mass-to-Charge (m/z)

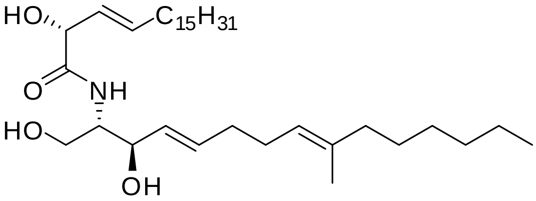
HO
C15H31
O
NH
HO
OH

6

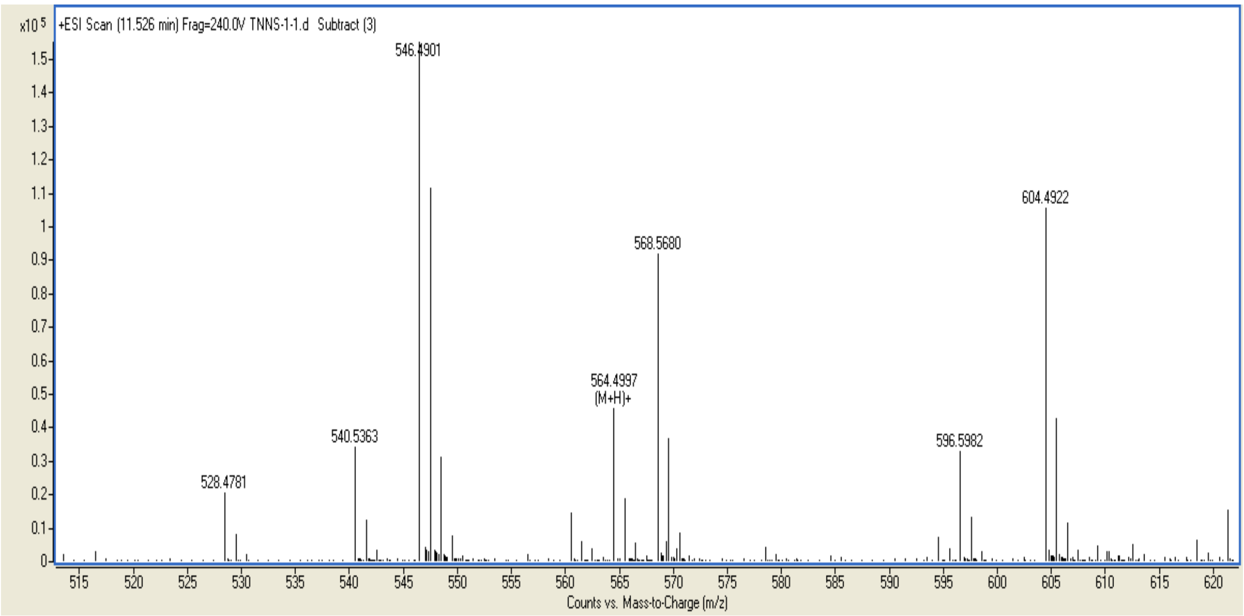
x10 5
+ESI Scan (11.526 min) Frag=240.0V TNNS-1-1.d Subtract (3)
546.4901
604.4922
568.5680
564.4997
(M+H)+
540.5363
596.5982
528.4781
Counts vs. Mass-to-Charge (m/z)

HPLC Spectra of compound 32

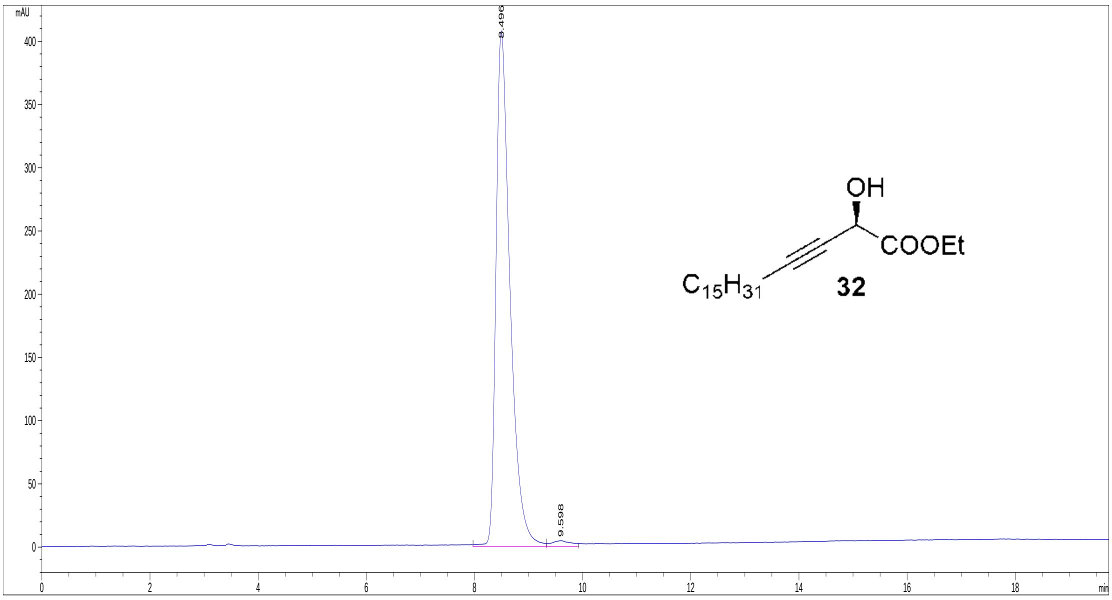

Peak #	RetTime [min]	Type	Width [min]	Area mAU	Height [mAU]	Area %
1	8.496	MF	0.2693	7381.4	407.9	98.412
2	9.598	FM	0.3272	119.1	4.6	1.588
Totals :				7500.4	412.5	

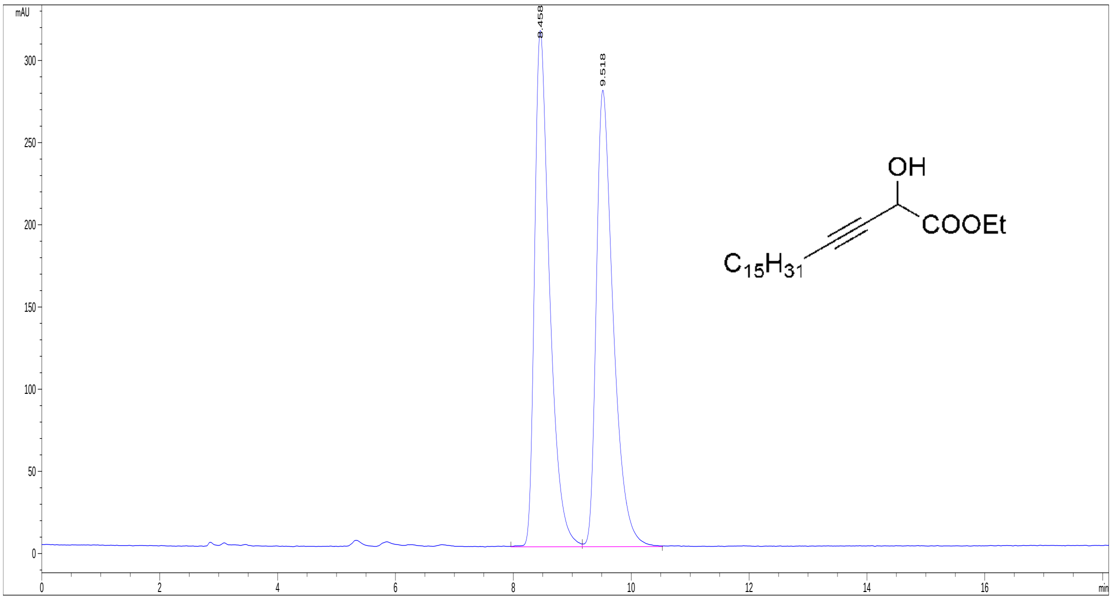

Peak #	RetTime [min]	Type	Width [min]	Area mAU	Height [mAU]	Area %
1	8.458	MF	0.2657	5584.6	314.1	50.185
2	9.518	FM	0.2984	5543.4	277.7	49.815
Totals :				11127.0	591.8	

Bioassay Protocols Data

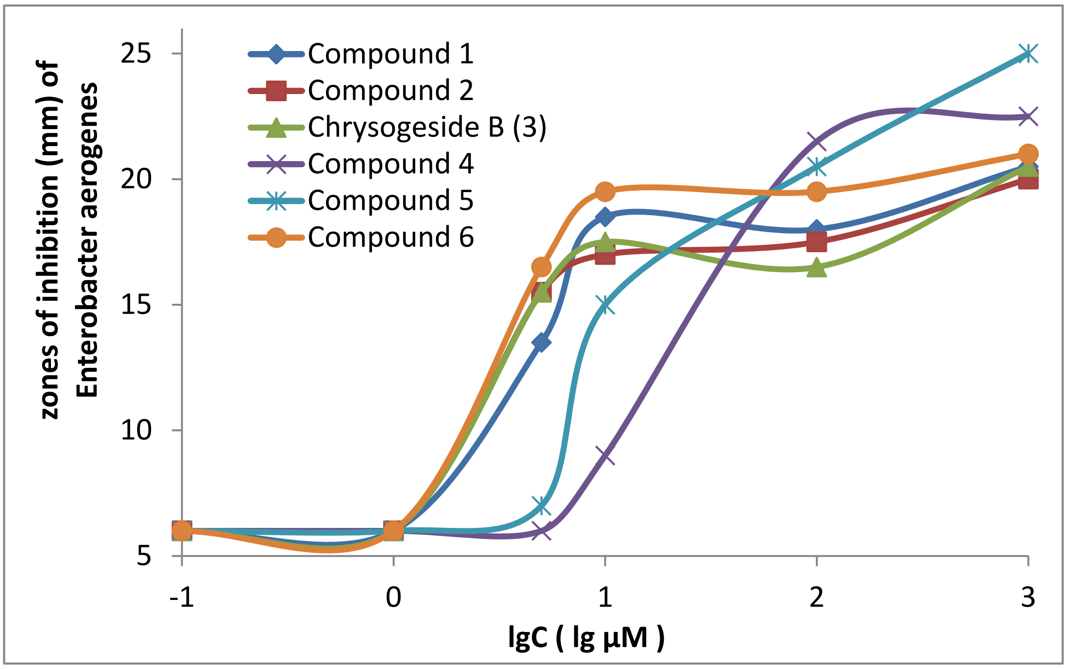

Figure 1. The antimicrobial activities against *Enterobacter aerogenes* with synthetic compounds **1** - **6** at different concentrations. Incubation after 24 h, and zones of inhibition (mm in diameter) were recorded.

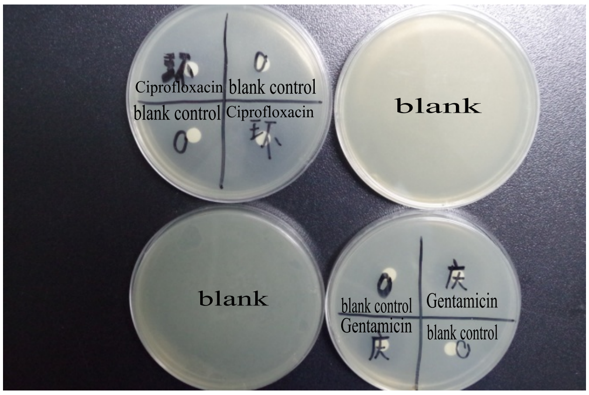

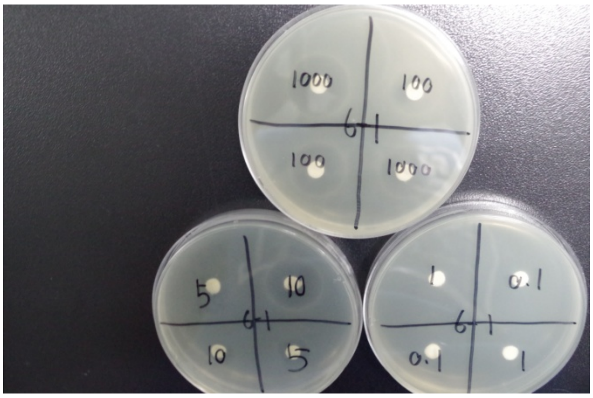

Ciprofloxacin (5 μg/disk), Gentamicin (10 μg/disk) and blank control (5 μL methanol/disk)

Compound 1

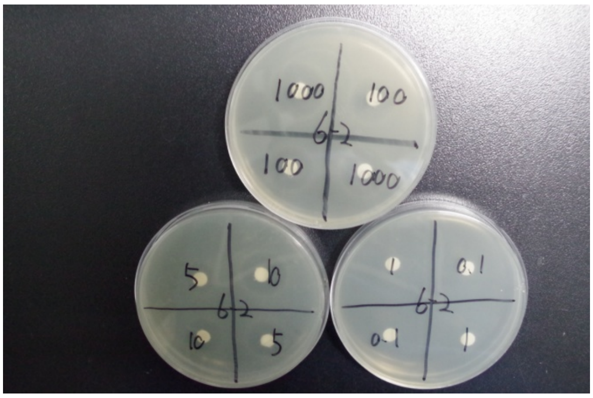

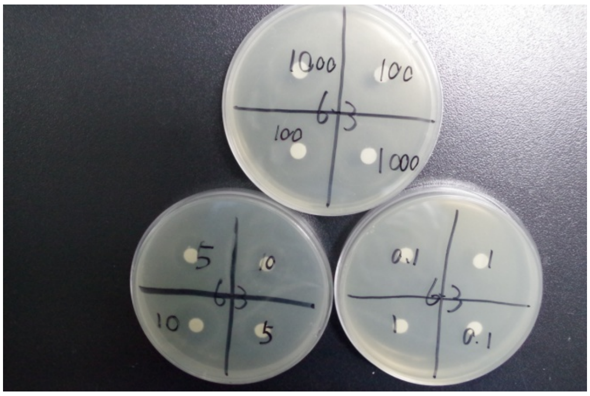

Compound 2

Chrysogeside B (Compound 3)

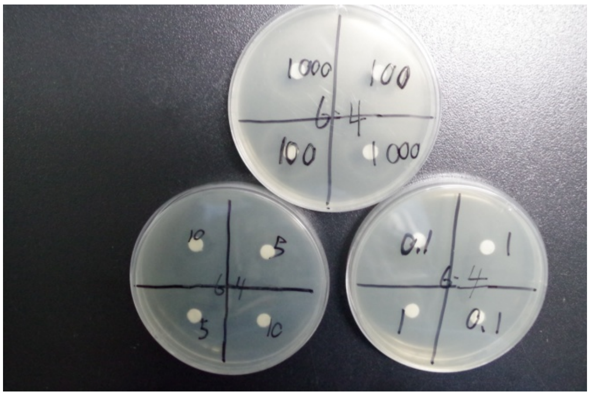

Compound 4

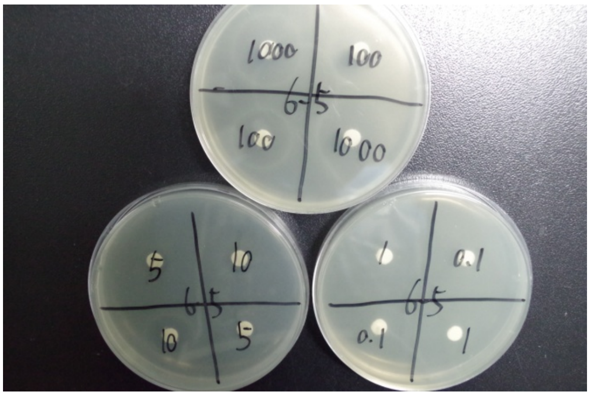

Compound 5

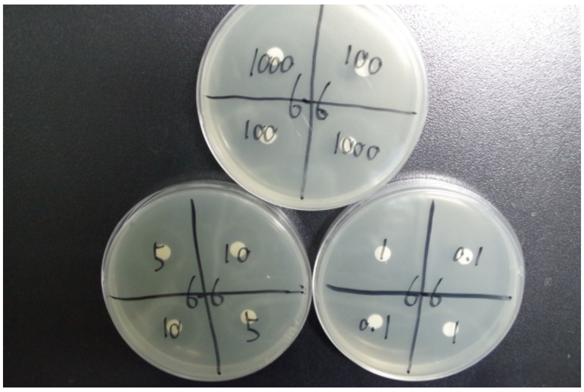

Compound 6

The antimicrobial activities against *Enterobacter aerogenes* with synthetic compounds **1** - **6** at different concentrations, Ciprofloxacin (5 µg/disk), Gentamicin (10 µg/disk) and blank control (5 µL methanol/disk). Incubation after 24 h, and zones of inhibition (mm in diameter) were recorded.

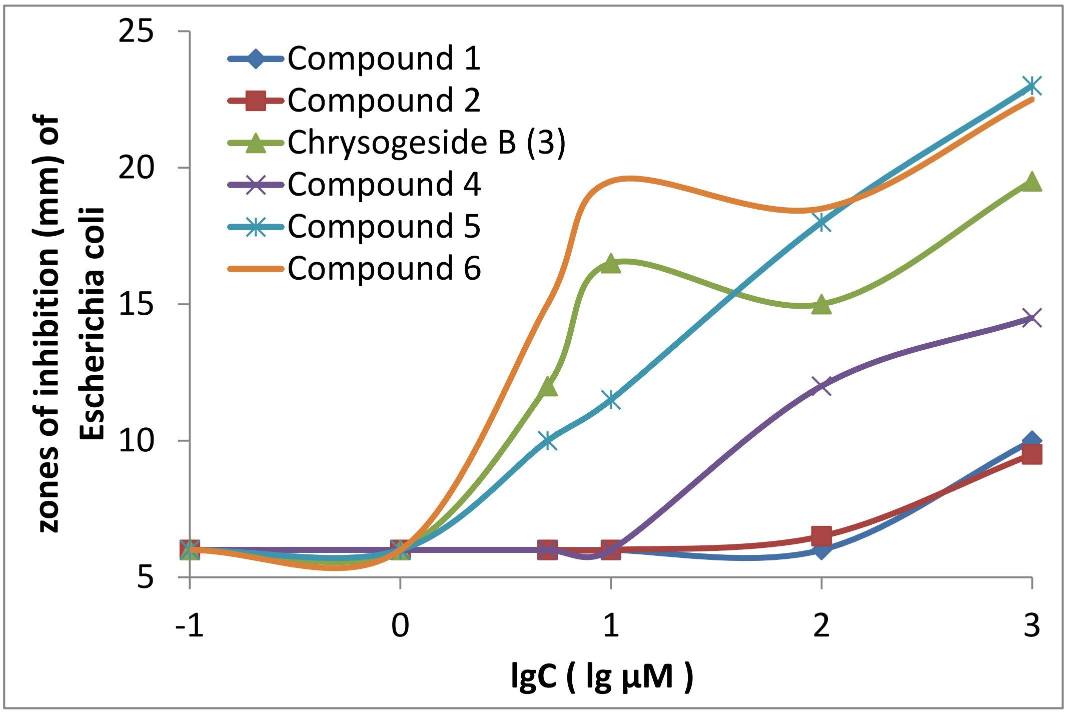

Figure 2. The antimicrobial activities against *Escherichia coli* with synthetic compounds **1** - **6** at different concentrations.

Incubation after 24 h, and zones of inhibition (mm in diameter) were recorded.

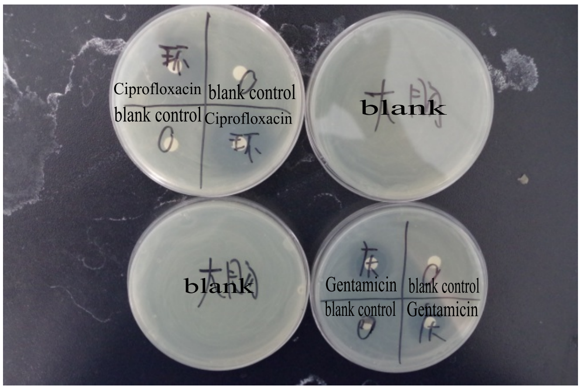

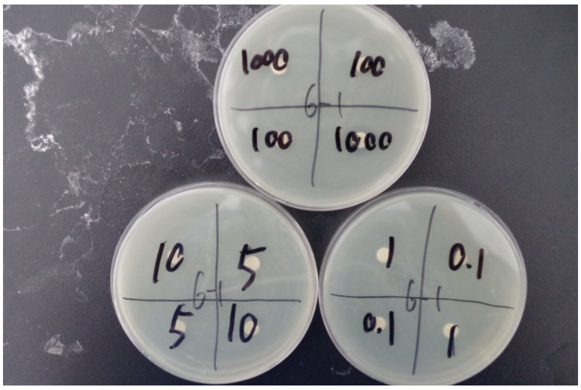

Ciprofloxacin (5 μg/disk), Gentamicin (10 μg/disk)

and blank control (5 μL methanol/disk)

Compound 1

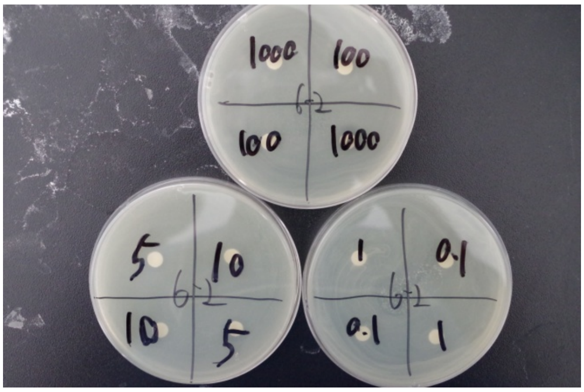

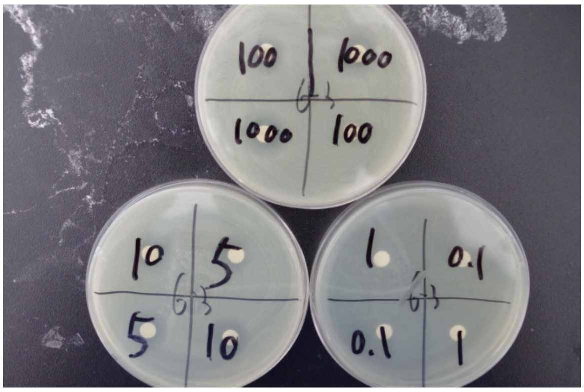

Compound 2

Chrysogeside B (Compound 3)

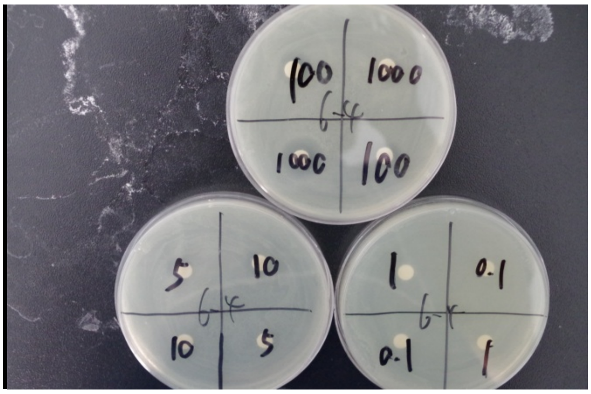

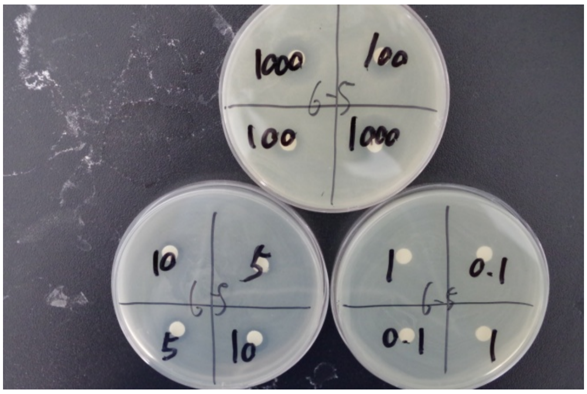

Compound 4 **Compound 5**

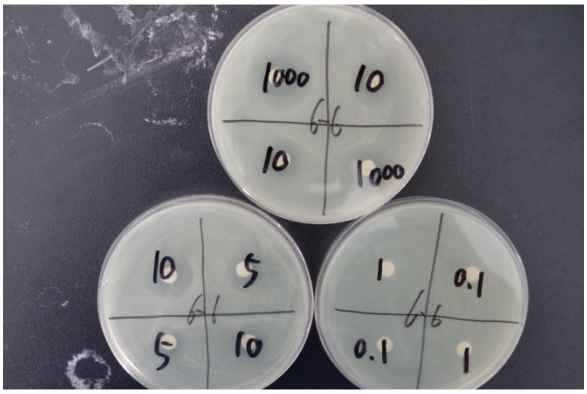

Compound 6

The antimicrobial activities against *Escherichia coli* with synthetic compounds **1** - **6** at different concentrations, Ciprofloxacin (5 μg/disk), Gentamicin (10 μg/disk) and blank control (5 μL methanol/disk). Incubation after 24 h, and zones of inhibition (mm in diameter) were recorded.

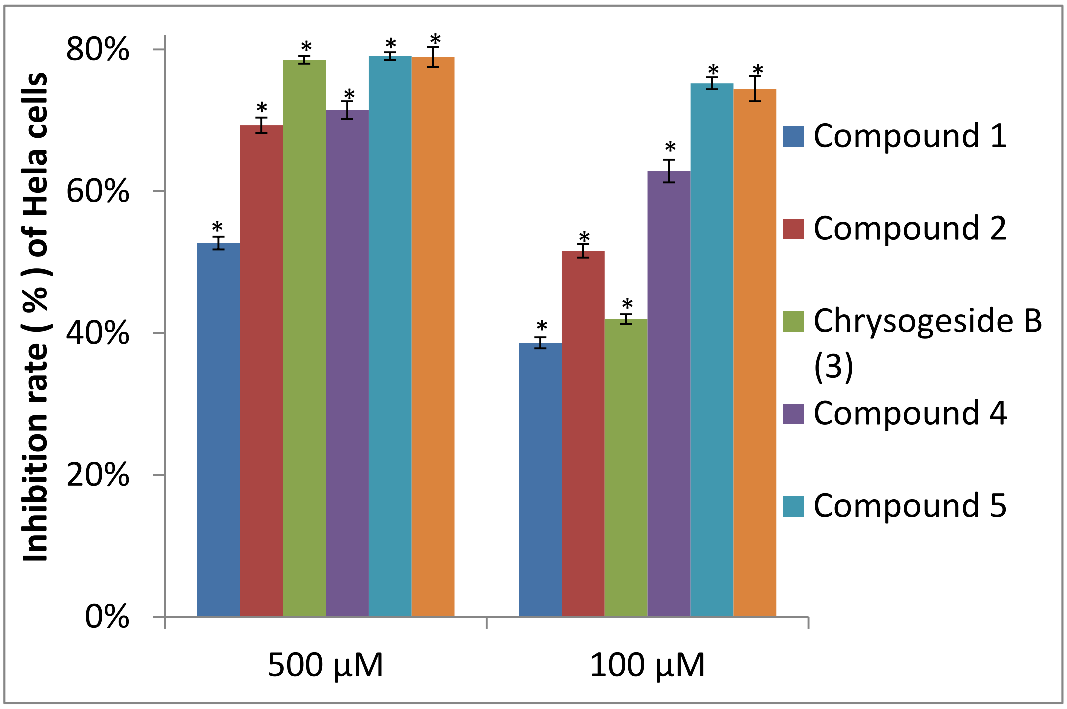

Figure 3. The cytotoxicity against Hela cells with synthetic compounds **1** - **6** at different concentrations by the MTT method. Data are expressed as means ± SD of the inhibition rate of Hela cells from synthetic compounds **1**-**6** at 100, 500 uM. *P < 0.01 vs control.

References

1 Black, F. J.; Kocienski, P. J. Synthesis of phalluside-1 and Sch II using 1,2-metallate rearrangements. *Org. Biomol. Chem.* **8(5)**, 1188-1193 (2010).

2 Mori, K.; Funaki, Y. Synthesis of sphingosine relatives. III. Synthesis of (4*E*,8*E*,2*S*,3*R*,2'*R*)-*N*-2'-hydroxyhexadecanoyl-1-*O*-β-D-glucopyranosyl-9-methyl-4,8-sphingadienine, the fruiting-inducing cerebroside in a basidiomycete Schizophyllum commune. *Tetrahedron.* **41(12)**, 2379-2386 (1985).

3 Abe, T.; Mori, K. V. Synthesis of sphingosine relatives. XV. Synthesis of (2*S*,2'*R*,3*R*,4*E*,8*E*)-*N*-2'-hydroxyoctadecanoyl-1-*O*-(β-D-glucopyranosyl)-9-methyl-4,8-sphingadiene (Pen III), a cerebroside isolated from Penicillium funiculosum as the fruiting inducer against Schizophyllum commune. *Biosci. Biotech. Bioch.* **58(9)**, 1671-1674 (1994).

4 Teiichi, M.; Reiko, H.; Kiyotaka, F. Efficient stereocontrolled synthesis of sphingadienine derivatives. *Tetrahedron.* **61(39)**, 9233-9241 (2005).

5 De Jonghe, S. *et al.* Synthesis of fluorinated sphinganine and dihydroceramide analogues. *Eur. J. Org. Chem.* **18**, 3177-3183 (2000).

6 Wayne, P.; Paul, V. M. $SnCl_4$- and $TiCl_4$-catalyzed anomerization of acylated *O*- and *S*-glycosides: analysis of factors that lead to higher α:β anomer ratios and reaction rates. *J. Org. Chem.* **75(20)**, 6747-6755 (2010).

7 Prévost Sébastien; Ayad Tahar; Phansavath Phannarath; Ratovelomanana-Vidal; Virginie. Total synthesis of symbioramide: a flexible approach for the efficient preparation of structural isomers. *Adv. Synth. Catal.* **353(17)**, 3213-3226 (2011).

8 Wang, L. *et al.* Catalytic enantioselective synthesis of optically active α-hydroxyl-β,γ-unsaturated acid esters as novel side chains of cerebrosides. *Tetrahedron: Asymmetry.* **24**, 173-177 (2013).

9 Peng, X. P. *et al.* Cerebrosides and 2-pyridone alkaloids from the halotolerant fungus *Penicillium chrysogenum* grown in a hypersaline medium. *J. Nat. Prod.* **74(5)**, 1298-1302 (2011).
